# Enzymatic tailoring of anionic glycans for characterizing lectin and antibody specificities in a microarray format

**DOI:** 10.1016/j.jbc.2025.110915

**Published:** 2025-11-05

**Authors:** Lena Nuschy, Iain B.H. Wilson

**Affiliations:** Institut für Biochemie, Universität für Bodenkultur, Vienna, Austria

**Keywords:** lectin, antibody, microarray, glycobiology, glycosyltransferase, sialic acid, anionic glycan

## Abstract

Despite many studies on binding patterns of commercial lectins and other glycan binding proteins, unexpected recognition motifs are still revealed, highlighting the necessity to (re-)investigate these specificities deeply, as they are used for rapid screening of glycosylation patterns or diagnostic histochemistry. The current work is an extension of previous defined glycan microarrays, displaying a library of natural anionic structures not only including *N*-acetylneuraminic acid but also *N*-glycolylneuraminic acid as well as sulfated galactose, *N*-acetylglucosamine or glucuronic acid based on using recombinant glycosyl- and sulfo-transferases. Moreover, anionic modifications on linear tetrasaccharides *versus* on typical biantennary N-glycan core structures were compared regarding binding pattern and intensities. As in our previous studies, 2-amino-*N*-(2-amino-ethyl)-benzamide (AEAB)-labeled glycans were probed with various plant lectins, C-type lectins, sialic-acid specific lectins, different antibodies, *e.g.* anti-NeuGc and recombinant prokaryotic lectins (RPLs). Indeed, expected binding patterns were observed; however, some proteins revealed more narrow specificities. The L2 anti-HNK-1 elicits its specificity for GlcAβ1-3Galβ1-4GlcNAc without the necessity of sulfation also for linear glycans. For anti-Le^X^ (clone L5) it is known that sialic acid masking of the epitope is not tolerated, while here we demonstrate that anti-Le^A^ antibody (clone T174) can interact with α2,3 Neu5Ac and Neu5Gc capped epitopes. HECA-452 recognizes sialyl Le^X^ and sialyl Le^A^, but only with Neu5Ac attached. This anionic glycan array contains most common anionic glycan modifications, can be flexibly modified and reveals commonly-overlooked specificities providing insights into sialic acid-specific interactions, including the often neglected *N*-glycolylneuraminic acid, thereby applying these findings also to murine or other deuterostome models.

Glycan microarrays have become a state-of-the art tool to study the interaction of glycan motifs with lectins, but also components of the mammalian innate and adaptive immune system including pentraxins and antibodies aiming for better understanding of the function of glycans and identification of (novel) epitopes. Major advances were made by the Consortium for Functional Glycomics (www.functionalglycomics.org), now the National Centre for Functional Glycomics, which has a large number of glycan array datasets from the past 20 years (1), complemented by the often more tailored data from other laboratories (2). Due to a limitation of defined oligosaccharides isolated from natural sources and sample complexity impeding separation of single structures, (chemo)-enzymatic synthesis of glycan libraries including common anionic and neutral glycan features of N- and O-glycans (3, 4) has become a favored approach. Overall, not only glycans alone but also other sugar harboring molecules like glycolipids or glycoproteins are probed with microarrays which are increasingly becoming a high-throughput method for glycomic analyses (reviewed in (5)). Reflecting the huge diversity of described glycan modifications in any format is a challenge, but anionic structures are of special interest. Undoubtedly sialic acids and their respective (not only viral) binding proteins play an important role in immunity and other cellular processes (6), also late-onset Alzheimer’s disease was associated with inhibitory sialic acid–binding immunoglobulin-like lectins (Siglecs) in the brain (7, 8).

Given the biological relevance, over the years many research groups have synthesized sialic acid containing N- and O-linked glycans and analyzed their interaction with several hemagglutinins or Siglecs (9, 10) while others studied the influence of additional sulfation using microarrays or even cell-based glycan arrays (7, 11, 12). It is well known that Neu5Gc does not naturally occur in humans, but traces might be incorporated from animal derived Neu5Gc-rich food (13); while it is the predominant sialic acid in mice except for the brain (as in general in vertebrates (14)), where it may only be found in cells originally from other organs infiltrating tumor tissues (15). The manifold role of (sialylated) Lewis antigens in physiological and pathological states is of great interest and widely discussed (16) due to their close relation to the ABO blood group system. Lewis epitopes, for example, serve as ligands for E-selectin (17), are involved in hematopoiesis (18), expressed on cancer cells and linked to prognosis (19, 20); sLewis^A^ (known as cancer antigen CA 19–9) is one of the most frequent tumor markers for digestive cancers (21). Also, the cutaneous lymphocyte antigen (CLA) is a described carbohydrate ligand for E-selectin, involved in lymphocyte homing after skin damage and cutaneous T-cell lymphomas, recognized by a distinct mAb clone HECA-452 (22). Although anti-Lewis antibodies due to their low reactivity at body temperature are considered to play a minor pathological role, nevertheless, cases of reactive patient antibodies have been reported (23, 24). This highlights the importance of pre-blood transfusion testing as well as the precise characterization of (monoclonal) antibody binding patterns and the rare but existing clinical significance of antibodies against Lewis-epitopes.

Overall, the goal was to analyze biantennary and linear glycans in terms of their accessibility and minimal requirements for lectin binding as well as the re-examination of designated anionic epitope recognizing lectins and antibodies, also regarding different types of sialic acids (see workflow; Fig. 1). Finally, we delivered valuable additional insights into glycan-protein interactions while exactly defining lectin and antibody specificities. Using several glycosyl- and sulfotransferases, a library of 51 compounds including N-glycans and ganglioside-type glycans could be synthesized and probed with established but also new commercial and customized lectins and proteins. Herein, we report the verification of many “off the shelf”-lectins, the identification of new epitopes for anti-CLA (HECA-452) as well as the precise determination of selected anti-HNK-1, anti-NeuGc, or anti-Le^A^ monoclonal antibodies and distinct Siglecs amongst others.

**Figure 1:**
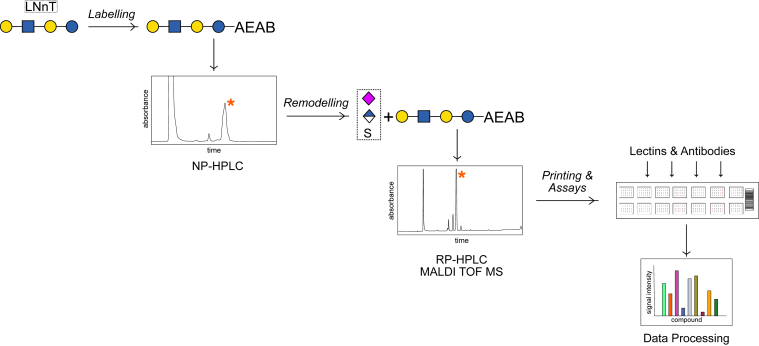
**Graphical overview of the applied workflow**. Labeled substrates were NP-HPLC purified, remodeled using several transferring enzymes and products purified by RP-HPLC and verified by mass spectrometry. In total, 51 defined glycans were printed onto a microarray and probed by a diversity of, *e.g.*, lectins or antibodies.

## Results

To offer insights into mono- and divalent ligands, the linear tetrasaccharides lacto-*N*-neotetraose, lacto-*N*-tetraose, and lacto-series ganglioside GA1-type glycans were labeled with the fluorescent linker AEAB by established procedures (25) and purified by NP-HPLC, while AEAB-labeled biantennary Gal-terminated N-glycans were purchased. Using a set of glycosyl- and sulfotransferases (Supplementary Methods), these substrates were modified with varying efficiencies (see Methods Section). The majority of previously reported enzymatic specificities could be confirmed, such as the preference of α2,6 sialyltransferases for the α3 Man antenna (26) (more pronounced in case of substrate limitation); however, for some sulfotransferases, we did not notice any difference in antennal preference or rather made contradictory observations (for further details see the Supporting Information). Bacterial *Neisseria meningitidis* and *Pasteurella multocida* α2,3 sialyltransferases elicited low conversion rates of LNnT, LNT, and N-type glycans, while lactose was still a good substrate for PmST3. Therefore, to generate enough product for printing or further enzymatic reactions, they were replaced by human ST3GAL4. This enzyme can only use non-fucosylated and non-sulfated substrates, preferring LNT over LNnT but also accepts asialoGM1 as a substrate (for a summary see Supplementary Methods, Table S2). Similarly, CHST1 did not accept Lewis-type structures (CHST2 and CHST10 only accepted terminal GlcNAc and GlcA, respectively). Unexpectedly, we detected several peaks in the chromatogram after Lewis-epitope synthesizing FUT3 treatment of sialylated LNnT or LNT which could be revealed as fucosylated glucose, either alone or in addition to fucosylated GlcNAc. Especially, when the substrate contained sulfated galactose, we report a tendency of FUT3 to transfer fucose to glucose instead of GlcNAc (Supp. page 49). This observation indicates that FUT3 can not only use GlcNAc but also reduced Glc as a substrate for modification, a rather uncommon observation. It is worth noting, that bacterial α-1,3/4 fucosyltransferase or *C. elegans* FUT6, on the other hand, accepted neither sialylated nor sulfated substrates. After modification, products were separated and purified by RP-HPLC, and finally verified by MALDI-10.13039/100001780TOF MS and MS/MS, partly in conjunction with diagnostic glycosidase digestions (Supporting Information; overview Fig. 1).

**Figure 2:**
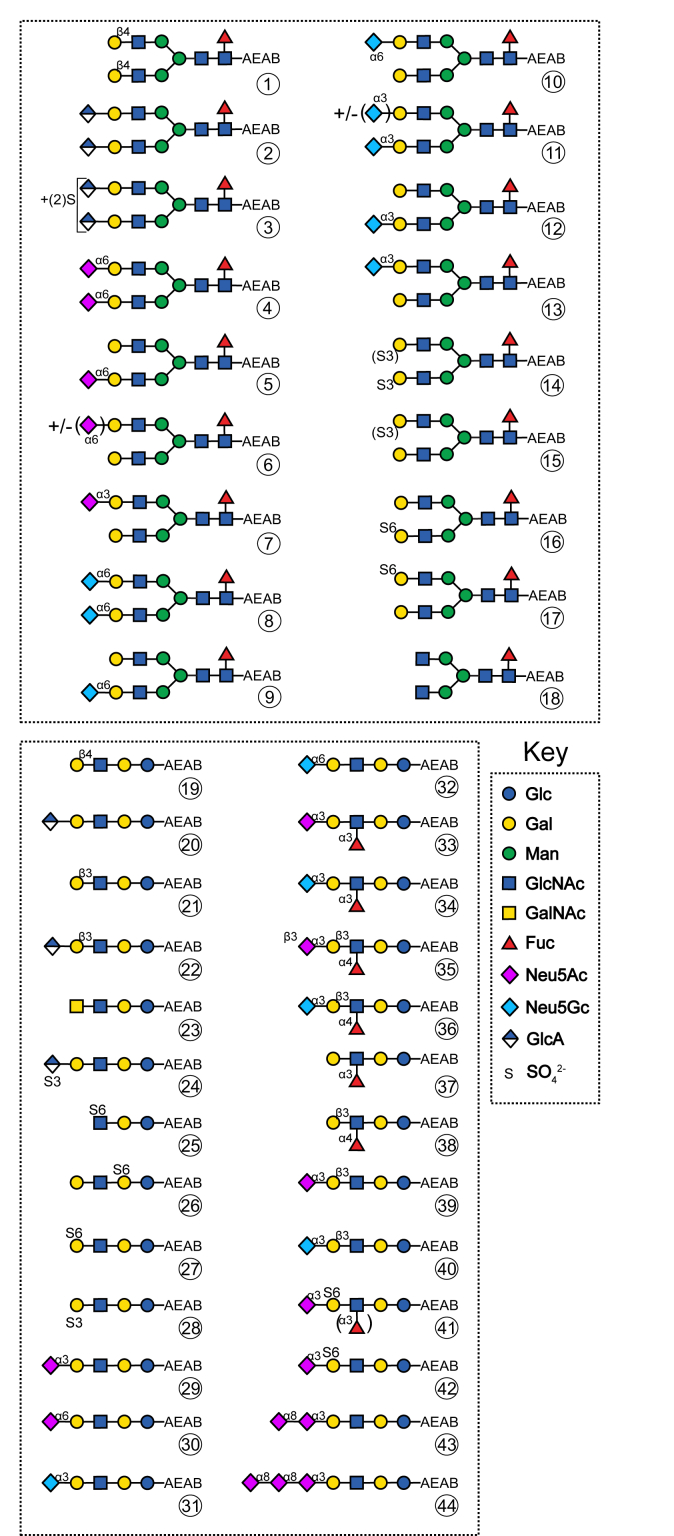
**Composition of the main 44 enzymatically modified glycans**. 18 biantennary N-glycans, 19 linear saccharides and 7 Lewis-type glycans were used in the microarray (**1**, **18**, **19**, **21**, **37** and **38** are biosynthetic precursor controls). For an overview of ganglioside-type glycans (**45–51**) please refer to Fig. S4*A*. The symbolic nomenclature for glycans is shown (65). Galactose is β4-linked unless otherwise stated, glucuronic acid is β3-linked.

In total, a library of 51 linear, Lewis-type and biantennary anionic glycans could be enzymatically synthesized (Fig. 2, Supporting Information). Aiming for identification of binding partners and testing (already known) specificities of various lectins, a microarray was constructed. A variable amount of 20 to 100 nmol of substrate for the enzymatic reactions, dissolved in 10 μl, allowed printing of multiple slides, sufficient for most practical applications. The employed substrate concentration was adjusted depending on the conversion rate of the enzymatic reaction to synthesize products with the same HPLC peak area. To validate the functionality of the established array, firstly common plant lectins *e.g.* ConA and LCA were applied (Fig. S1). Complementary to this, several lectins known to bind neutral (especially GalNAc/LacdiNAc) glycans, as well as selected recombinant prokaryotic lectins (RPLs), were tested (Figs. S2, and S3).

At first, all compounds were printed at the same amount. Binding of plant lectins and some antibodies could be detected even at minimal product amount (important for reactions with low conversion, *e.g.* sulfation reactions, to save costly donor substrate and enzymes); however, most Siglecs, for example, elicited no binding signal. Therefore, the printing concentration of glycans containing modifications suspected as binding partners for those lectins was doubled or even tripled and it was necessary to use the most efficient sialyltransferase (see further in Methods section) to obtain sufficient amounts of product. On the other hand, any further increase in printed glycan or probing lectin concentration only resulted in stronger background or unspecific binding.

We demonstrate that this glycan microarray is easily expandable in a targeted manner, while results are still comparable (no significant difference in binding data from different slides and printing runs, see Supporting Information), since binding signals increased mostly linear with respect to the amount of printed substance. During the development of the array, the library of structures was extended. All linear LNnT-based compounds were printed and probed at first, before biantennary glycans were included, further (branched) LNT/LNnT-structures added and finally ganglioside-type glycans synthesized, to assess particular specificities such as those of Siglecs and anti-Lewis antibodies. As a further proof of concept, two glycosaminoglycan tetrasaccharides, disulfated chondroitin and heparan units, were purchased, printed and probed as well, in order to represent the broad variety of glycan containing structures in Nature (Supporting Information). Due to low yields of some reactions or no suspected ligand recognition, not all combinations of remodeled compounds and lectins/antibodies were tested.

### Sialic acid-specific lectins and proteins

**Figure 3:**
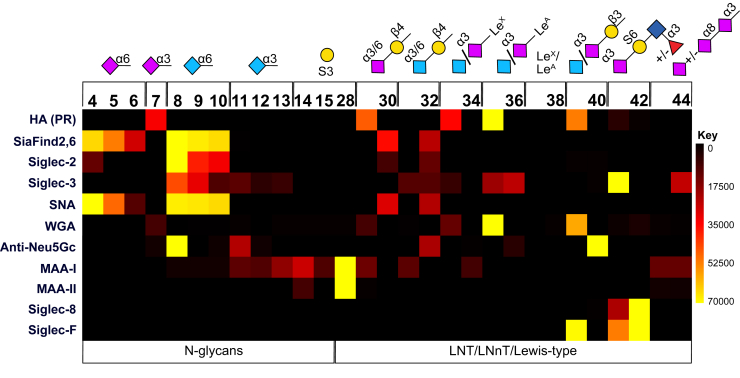
**Heat map displaying lectin binding to selected sialylated and sulfated structures**. The heat map was generated using the GLAD tool and represents the relative fluorescent units (RFU) normalized to 70,000 as the maximum value. Relevant epitopes are shown, structures including either Neu5Ac or Neu5Gc as terminal sialic acid or different linkages are indicated with a slash. For complete structures, please refer to Figure 2.

Specificities for different sialic acids, *N*-acetylneuraminic acid and *N*-glycolylneuraminic acid of designated α2,3 or α2,6 (also α2,8) sialic acid-binding lectins were compared. SNA, a plant α2,6 Sia-specific lectin bound to both Neu5Ac and Neu5Gc equally as previously reported (27). Binding strength increased with the number of sialic acid residues attached, thus signal intensities were weaker for the Neu5Ac (**30**) or Neu5Gc (**32**) terminated linear LNnT as compared to biantennary N-glycans (Fig. 3, Supporting Information). WGA on the other hand (in this array only tested for its ability to bind α2,3 linked sialic acid not GlcNAc), could only interact with α2,3 linked Neu5Ac but not Neu5Gc (28). Interestingly, binding signals were much higher for Neu5Acα2-3Galβ1-3GlcNAc/GalNAc (**35**, **39**, **46**) when compared to structures with an underlying β4-Gal linkage. Binding was diminished or abolished when either a further α2,8 sialic acid was attached (**43**, **44**, **47**), or the galactose was sulfated.

MAA-I, commonly considered as a α2,3-specific lectin, showed less binding towards α2,3 Neu5Ac than Neu5Gc in our array, regardless of fucosylation, while previous studies were not consistent (some observed the opposite (27), whereas Song and colleagues reported higher binding of KDN or acetylated/methylated sialic acids linked to β4-Gal (29)). In contrast to WGA, MAA-I only recognizes α2,3 sialic acid on β4-linked galactose, except for sialyllactose, which was not bound at all, but tolerates further α2,8 sialic acid chain elongation (**43** and **44**). However, interaction with SO_4_^-^ 3-Gal was much stronger and MAA-II bound 3-O-sulfated compounds (**14** and **28**) exclusively but generally elicited lower signals (Fig. 3). Furthermore, no binding to Galβ1-4GlcNAc could be measured although described in the binding patterns listed by the manufacturer (VectorLabs, Table S1).

Selected human (or mouse) Fc- or His-tagged sialic acid-binding immunoglobulin superfamily lectins, Siglec-2, -3, -5 (only on compounds **45–51**), −7, −8, and -F were tested on the array. In contrast to other studies (11), there was no need to pre-complex the lectin with antibody to achieve proper ligand binding. Although human Siglec-2 has been reported to have slightly higher affinity for Neu5Ac than Neu5Gc (30); interestingly, we observed stronger binding to Neu5Gc, even though the human version of the protein was applied. Similar to SNA and as known for Siglecs (3), binding signals decreased with fewer sialic acid modifications of the biantennary glycans, while being very low for α2,6 sialylated LNnT. Binding of Siglec-3 was in general weaker and less specific than Siglec-2 except for Neu5Acα2-3(6S)Galβ1-4(α3Fuc)GlcNAc (**41**) which was reported as an epitope implicated in Alzheimers’ disease (7), although without the fucose attached, while we were not able to measure binding to the non-fucosylated compound (Fig. 3). It was suggested that the inhibitory Siglec-3 (CD33) and Siglec-8 as well as the microglia-derived Siglec-F share the same ligand in the brain potentially contributing to Alzheimer’s disease (8). Indeed, we identified **41** and **42** as ligands for Siglec-8 and Siglec-F (Fig. 3); however, in contrast to Siglec-3, the non-fucosylated Neu5Acα2-3(6S)Galβ1-4GlcNAc structure was preferred by Siglec-F (**42**), while binding of Siglec-3 was only detectable with a fucose attached. Moreover, Siglec-F bound to Neu5Acα2-3Galβ1-3GlcNAc/GalNAc (**39** and **46**, respectively, Fig. S4*B*) but neither to the Neu5Gc- (which could be explained by the absence of Neu5Gc in the murine brain), underlying β4-Gal linkage- nor the Lewis-A type-version. It further recognized Neu5Acα2-3 modified lactose (**49**); however, binding was weaker than for the GM1b-type glycan. Interestingly Siglec-5 elicited a similar binding pattern as Siglec-F, however with opposite ratios, preferring the GM1b-type glycan over GM3 (Fig. S4*B*). Siglec-7 was obtained from two sources and tested for its ability to recognize structures resembling ganglioside GD3 which has been described as a ligand before (31). However, it bound neither compounds **43** and **44** with a similar structure as GD3 (but with a GlcNAc instead of a Glc) nor **50**, resembling the ganglioside-type glycan (data not shown). Noteworthily, in a previous array setting (32), no Siglec-7 binding to GD3 was detected either.

Among more recently developed commercial sialic acid detecting proteins (SiaFind α-2,3-specific; α-2,6-specific; Pan-specific and Pan-specific 2.0), only SiaFind2,6 (a recombinant protein engineered from *Polyporus squamosus* lectin (PSL); structure and binding mechanism resolved in (33)) was found truly specific for α2,6 linked sialic acid (Fig. 3). Binding of relevant SiaFind proteins to ganglioside-like compounds **45** to **51** is shown in Fig. S4*C*. While SiaFind2,3 bound sulfated structures similarly, and interestingly Neu5Gc in both linkages, binding was absent or at least very low for structures without sialic acid. Both Pan-specific SiaFind proteins elicited very low signals with all tested compounds regardless of presence of sialic acid (except for Lewis-antigens) but with significant backgrounds (supplementary graphs, Fig. S9). Similarly to SNA and Siglec-2, α-2,6-specific SiaFind recognized Neu5Ac as well as Neu5Gc. Binding of all α2,6-linked Sia specific proteins decreased with lower sialic acid content but to a different extent; while SNA and SiaFind2,6 signals for linear Sia terminated pentasaccharides were still quite strong, Siglec-2 binding was hardly detectable.

Hemagglutinin from Puerto Rico Strain Influenza A virus (passaged in SPF chicken eggs, H1N1) (34) showed a similar binding pattern as WGA, only recognizing α2,3 Neu5Ac not Neu5Gc, interestingly and similar to WGA, with higher affinity for sialic acid bound to β3-Gal (**39**) even increased by the presence of fucose (sLe^A^ epitope, **35**), suggesting an impact of the underlying Gal-linkage (Fig. 3). For specific detection of *N*-glycolylneuraminic acid, we applied a chicken anti-Neu5Gc antibody (35). Surprisingly, binding was only measured for selected structures: Either α2,6 linked Neu5Gc on β4-Gal (biantennary 8 and linear **32**) or LNT based compounds terminated with α2,3 linked Neu5Gc (**36**, **40**) suggesting a role of spatial saccharide arrangement. Slightly decreased binding was also detected for biantennary, but only fully α2,3 Neu5Gc capped N-glycan (**11**).

### Galactose-binding lectins

As a counterpart and in order to complete testing of the anionic microarray, the glycans were probed with galactose-binding lectins to measure the influence of diverse anionic modifications and how they impair interactions (Fig. 3). ECL is known to preferably bind Galβ1-4GlcNAc and GalNAc, rather than Galβ1-3GlcNAc (36). As expected, no signal was obtained when both antennae were modified (either glucuronylation, sialylation or sulfation), but only when one or both arms were free; the effect was similar when the terminal Gal of the linear saccharides was occupied (**26**). Sulfation of the internal Gal had no impact. Furthermore, as reported, no binding to β3-linked Gal was detectable for ECL nor RCA. While ECL binding is completely blocked by any modification that masks terminal β4-Gal, RCA can tolerate substitution (37) of the C6 by both sulfation (**27**) and sialylation – Neu5Ac and Neu5Gc - (**4**, **8**, **30**, **32**) but not the C3. Lectin-ligand interactions of all tested galactose-specific lectins were, however, reduced in the presence of an anionic modification. Contrary to ECL, no binding to GalNAc was detected with RCA; however, 4/6-O-sulfated GalNAc in a CS tetrasaccharide was recognized by this lectin (Fig. S5). The recombinant prokaryotic lectin recognizing galactose 1 (RPL-Gal1) requires at least one free antenna with terminal Gal (*e.g.* **7**, **9**, **12**, **13**), and bound best with one free arm and one sialylated, whereas binding affinities were interestingly lower when there was no terminal modification on both antennae and signals were mostly below detection limit for all linear saccharides (**19–36**). Interestingly, we noticed binding to **41**, a 6-O-sulfated Lewis X-type structure. Binding patterns of RPL-Gal4 were highly similar to RPL-Gal1; however, intensities significantly decreased and, in some cases, were hardly detectable (supplementary graphs, Fig. S8). No binding of RPL-Gal1 or -Gal4 to the ganglioside type glycans was observed. Galectin-1 was initially intended to serve as a positive control. Unexpectedly, specific binding was in total very weak, but the background signal was rather high, possibly a sign of denatured protein, impurities or poor quality of the lectin batch. Therefore, these data were finally excluded from the study. The GalNAc-containing analog of LNnT (**23**) was recognized by various lectins, including CLEC-10A, GSL-I and -II, PNA, SBA, VVA and WGA (Fig. S3), whereas CLEC-10A also bound to a CS tetrasaccharide (Fig. S5).

### Selected specific epitopes (CLA, Lewis antigens, and L2/HNK-1)

Finally, we decided to select relevant epitopes including an anionic modification for detailed analysis (Fig. 5). It was suggested before that the anti-Le^X^ antibody (clone L5) only binds to the non-sialylated epitope as detection of α1-acid glycoprotein on Western blot was observed only after sialidase treatment (38), and indeed, this was confirmed by our microarray study, whereby we could additionally verify that also Neu5Gc inhibits interaction (Fig. 5). Using the non-sialylated precursor (**37**) as a positive control, the functionality of the antibody was confirmed. In contrast, the deployed anti-Lewis A antibody (clone T174), previously little studied regarding the impact of sialylation, tolerates sialylated Le^A^ epitopes, moreover modification seems to even slightly increase the response signal and remarkably the antibody did not distinguish between Neu5Ac and Neu5Gc, but both elicited equal signal intensities; similar data was observed in a CFG screen (CFG Glycan Array ID: primscreen_5450). The HECA-452 antibody directed against the cutaneous lymphocyte-associated antigen was reported to recognize sialyl Lewis X (SSEA1), tolerating 6-O-sulfation of either the external Gal, GlcNAc or both (39, 40, 41, 42). Surprisingly, we observed binding of HECA-452 not only to sLe^X^ (**33**) but also to sLe^A^ (**35**); however, this was only when *N*-acetylneuraminic acid was attached but not Neu5Gc (Fig. 5). On the other hand, fucose was essential for proper binding and no signal was detected without it (**29**, **31**, **39**, **49**, data not shown). In contrast, no binding to 6-O-sulfated sLe^X^ (**41**) was observed, however, it cannot be excluded that this effect originates from the ratio of fucosylated GlcNAc to Glc as more Glc than GlcNAc in the compound mixture that was printed actually carried the fucose.

The HNK-1 epitope has always been described as HSO_3_−3GlcAβ1–3Galβ1–4GlcNAc (43, 44) from corresponding antibody interaction; therefore, we tested the anti-L2/HNK-1 antibody (clone L2-412) on selected GlcA and sulfate containing structures with two different underlying Gal linkages and the respective controls. Indeed, we observed the highest affinities with the intended epitope; however, interactions were not drastically hampered in the absence of sulfate (45), unlike the case for other monoclonal or natural HNK-1 antibodies (46). On the other hand, no signals were obtained for glucuronylated linear LNT-type structures (β3-linked Gal) (**22**, Fig. 5). Therefore, we conclude that sulfate is not required for efficient antibody binding on a microarray, but rather the β4-linkage of the Gal to which the glucuronic acid is attached is important. On the other hand, previous data indicated low binding to royal jelly N-glycans (25), as well as to insect glycoproteins and glycolipids (47), although the underlying backbones are based on a different motif, *i.e.*, Galβ1-3GalNAcβ1-4GlcNAc. In terms of other GlcA-containing compounds we also assessed binding of selected GBPs to chondroitin and heparan sulfate tetrasaccharides and could detect signals with BDNF (Fig. S5).

## Discussion

In summary, for most lectins and glycan binding proteins, no severe differences between biantennary N-glycans, Lewis-type glycans or linear saccharides could be observed. If a certain epitope is recognized, the length and complexity of the remaining sugar is not important, though, often the intensity of the binding signal depends on the number of occurring modifications (*e.g.* for all α2,6 sialic acid-specific lectins). Remarkably, RPL-Gal1 even required biantennary N-type structures for recognition. On the other hand, stronger anti-L2/HNK-1 binding was observed for the linear compounds rather than the biantennary, however the reasons remain elusive. More often there are other factors determining the intensity of the binding signals elicited, like a certain underlying Gal linkage or the presence or absence of additional modifications. The main focus of this study was to use a flexible enzymatic modification strategy to examine the designated binding patterns of commercial lectins and antibodies that are in the repertoire of most glycobiology labs, as well as testing some controversial specificities. Thereby this modular array applying easy-to-express or commercial enzymes should prove a useful tool for routine glycan analyses that apply these lectins for identification of epitopes, complementing several previous microarray studies (a.o. (3, 29, 48)). A potential application may be in clinics, where certain antibodies are used in histochemistry for typing tumors based on their binding patterns. This approach could be used to easily build an array with binding and non-binding partners of these antibodies to validate their functionality and specificity.

An extensive determination of lectin specificities tested on more than 600 compounds was previously performed using machine learning (49), while the current study is able to extend this collection by a range of GlcA- and Neu5Gc-containing structures. Interestingly, machine learning led to the assumption that fucosylation of the proximal GlcNAc may inhibit binding of MAA-I to sialylated epitopes in Lewis-type glycans; however, we detected binding to sLe^X^, but only for *N*-glycolylneuraminic acid (compound **34**, Fig. 3). On the other hand, we hereby report inhibition of MAA-I by 6′-O sulfated galactose to which the sialic acid is attached (**41** and **42**) even without a fucose or fucosylation of the more distant Glc. As many researchers do refer to vendor-defined specificities we have compared our data to those provided by VectorLabs (Table S1).

Indeed, we could confirm the specificities of SNA, WGA, MAA-I (other than machine learning results) and -II, ECL, all GalNAc binding plant lectins and CLEC-10A as well as the anti-Le^X^ antibody (for a summary see Fig. S6). For WGA (also PR8 H1N1 HA) and MAA-I (regarding sialic acid binding) we could define preferences for different Gal-linkages more precisely. WGA (and HA) for example showed higher binding for Siaα2-3Gal-β1-3GlcNAc than the corresponding LNnT-based compounds, while MAA-I does not bind to structures including β3-Gal at all. Interestingly, we found a higher affinity of Siglec-2 to Neu5Gc than to Neu5Ac, even though the human Fc-tagged version of the protein was applied. While RCA was reported to preferably bind to 6-sulfated β4-galactose (50), we did not observe sulfation-dependent enhanced signal intensities for Gal but for GalNAc, which was not recognized by RCA without modification, only with sulfate attached (binding to CS tetrasaccharide – Fig. S5). Furthermore, we were able to define the epitope of the monoclonal anti-L2/HNK-1 antibody (clone 412) more precisely, identify ligands of Siglec-8 and Siglec-F, characterize the binding patterns of chicken anti-Neu5Gc and anti-Le^A^ antibodies as well as provide additional new binding data of the HECA-452 antibody directed against CLA. Previous suggestions of broader, however distinct, recognition of the anti-L2/HNK-1 (412) antibody, which rather relies on the underlying galactose linkage than sulfate presence, opposed to other anti-HNK-1 mAbs, could be confirmed with our glycan microarray. Here, we emphasize that sulfate is not required for the binding of this particular mAb clone (412), which should be taken into consideration when the antibody is used for HNK-1 epitope identification.

All sialic acid binding proteins preferring α2,6 linkage recognized Neu5Ac and Neu5Gc equally, whereas for α2,3 specific lectins and other proteins like the tested haemagglutinin large differences were observed. Considering these results, some lectins are applicable for mouse or other mammalian tissue and sample analyses, while others are restricted to Neu5Ac containing human glycans only. Although it is known for more than 20 years now that *Maackia* lectins prefer 3-O-sulfation of Gal over α2,3 sialic acid (51) and research groups have recently advised against using MAA-I and MAA-II as histochemical reagents (48, 52), the scientific community still considers *Maackia* lectins as specific for α2,3 sialoglycans. The results obtained during the current microarray study once again highlight the necessity to include at least a neuraminidase step and compare prior and post treatment when MAA-I and -II are used as diagnostic tools. HA (PR8 strain) and WGA elicit the same binding pattern (although WGA with lower binding) but on the other hand never tolerate 3-O-sulfation. These findings suggest different types of α2,3 sialic acid binding lectins: one (including MAA or SiaFind2,3) that rather recognizes the linkage of the Gal (β4) and Gal-modification (on C3) and does not differentiate between Neu5Ac, Neu5Gc or sulfate, while others (HA PR8 or WGA) specifically interact with α2,3 *N*-acetylneuraminic acid regardless of the underlying Gal linkage. Additional α2,8-sialic acid did not inhibit MAA-I. Generally, for certain Sia-binding lectins and antibodies like the anti-NeuGc antibody, a role of the distinct conformation is suggested.

For fucosylated epitopes anti-Le^X^ (clone L5) antibody was already described and here confirmed to exclusively recognize the non-sialylated Le^X^ epitope, no conclusive reports were found for anti-Le^A^ (clone T147). Researchers should be cautious when applying it for Lewis A antigen identification as we found that it could also interact with both α2,3 Neu5Ac and α2,3 Neu5Gc masked epitopes. For commercial RPL-Gal1 and RPL-Fuc1 we could identify their interactions with glycans and reveal preferences; for example, even though RPL-Fuc1 specifically reacted with fucose, affinities for different linkages could be ordered as follows: α6>α4>α3.

From a qualitative perspective, all obtained results are comparable while a certain limitation in quantitative data comparison needs to be pointed out. On the one hand, each lectin or antibody was individually applied at a suitable concentration at which epitope recognition was observed while keeping background or unspecific binding low. On the other hand, substrate conversion varied, and products must be normalized based on HPLC peak areas; therefore, we conclude that binding signal intensities of glycans to several lectins should be compared in the light of exact methodology.

Although glycan array analyses on glass slides were introduced 20 years ago (53), they have only recently become a tool that is frequently in use in many glycobiology research labs for interaction studies. Users benefit from the high-throughput workflow and the relatively short time of 2 days, it takes a glycan to be printed and probed once it is derivatized. The approach of enzymatic epitope synthesis described here circumvents the need for often laborious, complicated, or expensive chemical synthesis and offers the possibility to readily synthesize a set of tailored epitopes beyond of those which are commercially available. This applies especially for N-glycans, as starting materials can be easily purchased, but for O-glycans there are more limitations in terms of suitable substrates, and chemical synthesis may still be needed. In general, the applied approach was successful; however, for sulfated compounds, a 2D-HPLC approach would increase purity. A further limitation is that each laboratory has slightly different glycan array methodology, *e.g.* linker, surface chemistry, detection mode and scanner type, which does complicate inter-laboratory comparisons. However, our data are in-line with most previous studies on any particular glycan-protein interaction and binding signals increased linearly with the amount of printed glycan; but also show that easily obtainable detection reagents are not available for every anionic glycomotif.

Certainly, analysis of lectin binding has been the topic of much past research, but is an ongoing endeavor. We would like to highlight that the use of relevant controls is of high importance when examining the specificities and “un”-specificities of distinct lectins or of glycan-binding proteins when applied for epitope identification or as diagnostic tools. Our modular approach is designed to be extended to validate lectins as tools in biochemical research and facilitates wider access to arrays based on enzymatically modified glycans.

## Methods

### Glycoenzymes and substrates

Lacto-*N*-neotetraose (LNnT) and lacto-*N*-tetraose (LNT) were purchased from Dextra; asialo-GM1 oligosaccharide (aGM1) from Elicityl, Crolles, France; chondroitin sulfate tetrasaccharide (unsaturated) from Biosynth Ltd; heparan sulfate tetrasaccharide from Sussex Research Laboratories Inc.; AEAB-labeled biantennary glycans with or without terminal Gal were synthesized by NatGlycan. Recombinant β1;(3)/4 galactosidase from *Aspergillus nidulans* (4.4 mg/ml) was used, β-*N*-acetylhexosaminidase from *Canavalia ensiformis* (jack bean) was obtained from Sigma-Aldrich, St Louis, MO, US; α-mannosidase from jack bean from ProZyme. β1,3-glucuronyltransferase (B3GAT1), β1,3-galactosyltransferase (B3GalT5), carbohydrate sulfotransferases 1 (CHST1), 2 (CHST2) and 10 (CHST10), galactose-3-O-sulfotransferase 2 (GAL3ST2), β-galactoside α2,3-sialyltransferase 4 (ST3GAL4) and fucosyltransferase 3 (FUT3); all human, were purchased from R&D Systems; α2,8-sialyltransferase from *Campylobacter jejuni* (CstII) from ChemilyBio; mutant β1,4-galactosyltransferase (Gal-T1 (Y289L)) for *N*-acetylgalactosamine transfer was part of the Click-IT O-GlcNAc Enzymatic Labeling System from ThermoFisher, and *Pasteurella multocida* α2,3-sialyltransferase (PmST3) purchased also from Sigma-Aldrich. *Neisseria meningitidis* α2,3-sialyltransferase (NmST3) (54) and *Photobacterium damsela* α2,6-sialyltransferase (PdST6) (55) genes were expressed in house. Donor substrates 3′-phosphoadenosine-5′-phosphosulfate (PAPS), cytidine 5′-monophosphate-*N*-acetyl-β-neuraminic acid (CMP-Neu5Ac), cytidine 5′-monophosphate-*N-*glycolyl-β-neuraminic acid disodium salt (CMP-Neu5Gc), guanosine 5′-diphospho-β-L-fucose disodium salt (GDP-Fuc), uridine-5-diphospho-*N*-acetyl-α-galactosamine sodium salt (UDP-GalNAc), uridine-5-diphospho-α-glucuronic acid trisodium salt (UDP-GlcA), uridine-5-diphospho-α-galactose (UDP-Gal) were purchased from R&D Systems (PAPS), Jennewein Biotechnologie GmbH (CMP-Neu5Ac and GDP-Fuc); ChemilyBio (CMP-Neu5Gc) and Sigma-Aldrich (all others) respectively.

### Lectins and Primary antibodies

Biotinylated lectins concanavalin A (ConA, 5 mg/ml), *Erythrina cristagalli* lectin (ECL, 5 mg/ml), *Griffonia simplicifolia* isolectin B4 (GSL-I, 0.5 mg/ml) and II (GSL-II, 2 mg/ml), *Lens culinaris* lectin (LCA, 5 mg/ml), *Maackia amurensis* lectin I and II (MAA-I, 2 mg/ml; MAA-II, 1 mg/ml), peanut lectin (PNA 5 mg/ml), *Ricinus communis* lectin I (RCA, 5 mg/ml), *Sambucus nigra*/Elderberry Bark lectin (SNA, 2 mg/ml), *Vicia villosa* lectin (VVA, 2 mg/ml), *Wisteria floribunda* agglutinin (WFA, 2 mg/ml) and wheat germ agglutinin (WGA, 5 mg/ml), as well as fluorescein-labeled soybean lectin (SBA, 2 mg/ml) were purchased from Vector Laboratories. His-tagged hemagglutinin (HA) of A/Puerto Rico/8/1934 (H1N1) was kindly provided by Prof. Reingard Grabherr (34). Fc-tagged human Siglec-2, Siglec-3, Siglec-7 and Siglec-8 were purchased from ACROBiosystems, Fc-tagged murine Siglec-F and human Siglec-5 and -7 from R&D Systems. Biotinylated SiaFind α-2,3-specific; α-2,6-specific; Pan-specific 1.0 and Pan-specific 2.0 were obtained as kind gifts from Lectenz Bio; Hexa-His-tagged RPL-Gal1, RPL-Gal4 and RPL-Fuc1 from GlycoSeLect Ltd, Dublin, Ireland. Anti-L2/HNK-1 antibody (clone 412) was kindly provided by Dr Hans Bakker (Medizinische Hochschule Hannover). His-tagged recombinant human C-type lectin domain family 10 member A (CLEC-10A) and recombinant human brain-derived neurotrophic factor (BDNF) were purchased from R&D Systems, His-tagged C-type lectin domain family 14 member A (CLEC-14A) was kindly provided by Prof. Christoph Rademacher (Universität Wien). Anti-Le^A^ antibody (clone T174) from mouse was obtained from Calbiochem; rat anti-Le^x^ antibody (L5) was a kind gift from Prof. Melitta Schachner and Dr Gabriele Loers (Universität Hamburg). His-tagged human galectin-1, anti-Neu5Gc antibody and AlexaFluor 647 conjugated anti-human/mouse cutaneous lymphocyte antigen CLA (clone HECA-452) were purchased from BioLegend, Inc.

### Secondary antibodies

Anti-His antibody clone HIS-1 IgG from mouse and anti-chicken IgY from rabbit were obtained from Sigma-Aldrich; anti-human-galectin-1 from rat from BioLegend; anti-human-BDNF from mouse (R&D Systems); AlexaFluor 647 conjugated streptavidin, anti-rat IgG and IgM, anti-rabbit IgG and anti-mouse IgG were purchased from Invitrogen ThermoFisher; AlexaFluor 647 conjugated anti-human IgG (H + L) from JacksonImmuno.

## Experimental procedures

### Labeling of LNnT

Procedure of free LNnT (as well as LNT, aGM1, CS and HS tetrasaccharides) modification with 2-amino-*N*-(2-amino-ethyl)-benzamide (AEAB; excitation/emission of 330/420 nm) was similar to Hykollari *et al.*, 2018 (25). In brief, the sample was dried after labeling and washing was achieved by four times precipitation of AEAB-labeled LNnT in cold 10:1 acetonitrile:H2O at −20 °C for 1h. In order to remove excess AEAB-label, the sample was purified by normal-phase HPLC (Zorbax NH2 Column 4.6 × 250 mm, 5-micron with guard column 4.6 × 12.5 mm 5-micron; Agilent Technologies, Inc) using a Shimadzu Prominence system (RF-20A XS Fluorescence Detector). Conditions were 0.8 ml/min flow rate, 80% acetonitrile in 125 mM ammonium acetate buffer pH 4.5, with fluorescence detection. Verification of the substance was ensured by MALDI-TOF MS and MS/MS (rapifleX TOF/TOF; Bruker Daltonics Corporation). Purification of CS and HS slightly differed from the other compounds and is described in more detail in the Supporting Information.

### Enzymatic synthesis of the glycan library

All incubation steps were carried out at 37°C for 16 h unless otherwise stated and amounts of commercial transferases per 50 μl reaction indicated (homemade enzyme concentrations were batch-dependent). Progress of the reactions was tracked by MALDI-TOF MS, in combination with β4-galactosidase digestion where appropriate. For ST3GAL4 and FUT3, LNT was observed as a preferred substrate, while B3GAT1 was more efficient on β4-Gal based glycans (Table S2). Protein concentrations suggested by the provider were usually well applicable for these enzymes, as well as for B3GalT5 and Gal-T1. On the other hand, product amounts formed by sulfotransferases within one reaction assay were rarely sufficient and increasing the enzyme concentration lacked the desired outcome; therefore, assays were repeated several times and product fractions added.

#### Sialylation (compounds 4–13, 29–36, 39–44, 46–47, 49–51)

AEAB-LNnT, AEAB-LNT or biantennary AEAB-GalGal were incubated with purified NmST3 (**7**, **29**) or PdST6 (**4–6**, **8–10**, **30**, **32**), 1 mM CMP-Neu5Ac or CMP-Neu5Gc and 5 mM MnCl_2_ in 25 mM TrisHCl buffer pH 7.5, respectively. For synthesis of Lewis-type, Neu5Gc-modified, ganglioside GA1-based glycans and those serving as substrates for further α2,8 Sia or 6-sulfate addition (**11–13**, **31**, **33–36**, **39–44**, **46–47**), ST3GAL4 (0.4 μg, R&D Systems) instead of NmST3 (homemade recombinant) was used to increase efficiency, since native bacterial 2,3-sialyltransferases were reported to have substantial donor hydrolysis activity (56). (*P. multicida* sialyltransferase was not able to convert sufficient amounts of those substrates despite repeated short incubations). ST3GAL4 assays were incubated in Tris buffer at pH 7 for 6 h. On the other hand, PmST3 (0.015 U) was sufficiently active on lactose (**48**) after previous digestion of LNnT with β4-galactosidase and jack bean hexosaminidase, forming **49** and further **50 to 51** by incubation at pH 8 for 30 min. Compounds **43** to **44**, **47** and **50** to **51** were synthesized incubating the α2,3-sialylated substrates with *CstII* (0.03 U) for 20 min with the same conditions as all other Sia-assays. Although for *CstII* sialidase activity was also reported (57), under the applied conditions we obtained full substrate conversion.

#### Fucosylation (compounds 33–38, 41)

Sialylated (and optionally sulfated, **41**) and non-sialylated (**37–38**) AEAB-LNnT or -LNT (intermediates purified by HPLC) was incubated with 1.5 mM GDP-Fuc, FUT3 (0.8 μg, R&D Systems) and 10 mM MnCl_2_ in 25 mM TrisHCl buffer pH 7.5. (Bacterial α-1,3/4 fucosyltransferase from ChemilyBio and in-house *C. elegans* FUT6 did not accept sialylated substrates, while ST3GAL4 inefficiently sialylates Lewis-type structures; Table S2).

#### Removal of terminal β4-Gal and neutral terminal modification (compounds 21–23, 25, 48)

*Asp. nidulans* galactosidase was added to labeled LNnT in 0.1 M ammonium acetate buffer pH 6 and incubated for 4 to 16 h. To synthesize **48** (LacCer imitating), the enzyme was inactivated after conversion was verified and hexosaminidase from *C. ensiformis* (jack bean) added for further 4h. Reaction mixtures to achieve β3-galactose addition (**21** and **22**) included further 10 mM MgCl_2_, 10 mM MnCl_2_, 0.2 mM UDP-Gal, B3GalT5 (0.9 μg, R&D Systems) and 100 mM TrisHCl buffer pH 8.5 and were again incubated, following previous heat-inactivation at 95°C for 3 min. For terminal *N*-acetylgalactosaminylation (**23**), after heat-inactivation, samples were incubated with 10 mM MnCl_2_, 0.1 mM UDP-GalNAc and Gal-T1 (Y289L, 2 μl – concentration not stated, ThermoFisher) in 110 mM HEPES buffer pH 8.0 at 4 °C.

#### Glucuronylation (compounds 2, 3, 20, 22, 24)

Biantennary AEAB-GalGal and AEAB-LNnT with terminal β3 or β4 Gal, respectively, were incubated with B3GAT1 (0.3 μg, R&D Systems), 0.625 mM UDP-GlcA, 5 mM MnCl_2_, 5 mM CaCl_2_ in 25 mM TrisHCl buffer pH 7.5 for 5 h (**3**, **24**) or 16 h (**2**, **20**, **22**).

#### Sulfation (compounds 3, 14–17, 24–28, 41–42)

After 5 h of incubation, 15 mM MgCl_2_, 0.2 mM PAPS and CHST10 (0.7 μg, R&D Systems) were added to preceding substrate glucuronylation for sulfate transfer onto GlcA (**3**, **24**). For 6-sulfation of galactose (**16**, **17**, **26**, **27**) reaction mixtures contained biantennary AEAB-GalGal or labeled LNnT, 15 mM MgCl_2_, 0.2 mM PAPS and CHST1 (1 μg, R&D Systems) in 25 mM TrisHCl buffer pH 7.5. Compounds **41** and **42** were synthesized using sialylated LNnT as substrate for sulfation (and further fucosylation, **41**). For 3-sulfation of galactose (**14**, **15**, **28**) mixtures contained 0.13 mM PAPS, GAL3ST2 (0.3 μg, R&D Systems), 15 mM MgCl_2_, and 50 mM MES buffer pH 5.5. 6-Sulfation assays of GlcNAc following previous β4-Gal removal of the labeled LNnT substrate included further 15 mM MgCl_2_, CHST2 (0.8 μg, R&D Systems), 0.2 mM PAPS and 100 mM TrisHCl buffer pH 8.5 (**25**). All enzymatic reactions were incubated for 16 h. Reactions aimed at the addition of 6-sulfate to terminal GlcNAc residues in biantennary AEAB-GnGn were inefficient, therefore these products were excluded from the library. Preliminary trials using CHST5 as an alternative indicated low efficiency with the commercial enzyme.

### HPLC purification and verification by MALDI-TOF MS

After efficient substrate conversion was confirmed by MALDI-TOF MS, products were separated and purified by reversed-phase HPLC (Kinetex LC column 5 μm XB-C18 100 Å, 250 x 4.6 mm with SecurityGuard ULTRA Holder; Phenomenex Inc) using a Shimadzu Prominence system (RF-20A XS Fluorescence Detector). A two solvent gradient with buffer A (0.1 M ammonium acetate pH 4) and buffer B (30% MeOH) was applied at a flow rate of 0.8 ml/min (for detailed gradients refer to Supporting Information). Fractions were analyzed by MALDI-TOF MS and MS/MS. Sulfate position on **25** to **28** was determined by adding *Asp. nidulans* β4-galactosidase and/or JBHex as appropriate in 0.1 M ammonium acetate buffer pH 5. For the biantennary compounds **5** to **7**, **9** to **17** a combination of *Asp. nidulans* β4-galactosidase, β-*N*-acetylglucosaminidase from jack bean and α-mannosidase from jack bean preferring α-1,3 linkage (+2 mM zinc), to define the antennae where sulfate and sialic acid are positioned, was applied and incubated in 0.1 M ammonium acetate buffer at 37°C for 16 h. Galactosidase digestion of compounds **41** and **42** (Fig. 2) was omitted, since only one peak containing sulfated and sialylated LNnT was found during HPLC purification post CHST1 treatment and in our previous assays the enzyme has been most efficient in sulfate addition to the terminal galactose. Furthermore, we suggest that the 6-sulfate is primarily subterminal and not internal due to FUT3 preferring the more distant glucose instead of GlcNAc; finally, a mixture of fucosylated Glc and GlcNAc was obtained. Resulting products were verified by MALDI-TOF MS (Supporting Information). Amounts were normalized before printing by the respective fluorescent intensities of peaks. In our setting, using high detector sensitivity (Gain x4, RF-20A XS Fluorescence Detector), an intensity of 1 x 10^8^ was required for adequate printing and detection by most plant lectins and antibodies, while for recognition by Siglecs the intensity was aimed at 3 × 10^8^.

### Microarray printing, analysis, and data processing

The synthesized glycan library was printed in two different concentrations, five replicates each, onto NHS-derivatised Nexterion H glass slides (Schott) and prepared for assays as described (25, 58). Glycans were rehydrated in TSMBB (1x TSM, 1% BSA, 5 mM CaCl_2_ and 0.05% Tween-20) before incubation steps were carried out with the respective lectin or antibody diluted in TSMBB for 1 h with shaking. Slides were washed between the steps as well as at the end with TSMWB (1x TSM + 0.05% Tween-20) followed by water, four times each, before drying them completely. All biotinylated lectins were applied in 1:100 dilution; biotinylated SiaFind reagents were applied at 5 μg/ml except for SiaFind α2,6 (0.5 μg/ml); and AlexaFluor 647 conjugated streptavidin (2 μg/ml) was used for detection. Fluorescein-labeled SBA as well as AlexaFluor 647 labeled HECA-452 (1:100 diluted) was detected directly. Incubation with HA (200 μg/ml), with CLEC-10A (10 μg/ml), or with CLEC-14A (200 μg/ml); RPL-Fuc1, RPL-Gal1 and RPL-Gal4 (20 μg/ml; pre-incubated for 45 min with 1 mM CaCl_2_, MgCl_2_ and MnCl_2_) was followed by anti-His HIS-1 IgG from mouse (1:1000 diluted) and AlexaFluor 647 conjugated anti-mouse IgG (2 μg/ml). Anti-L2/HNK-1 antibody (clone 412) was used 1:1000 diluted, anti-Le^A^ 1:25, both detected with AlexaFluor 647 conjugated anti-mouse IgG (2 μg/ml) as well. Anti-Le^X^ (1:400) incubation was followed by AlexaFluor 647 anti-rat IgM (2 μg/ml). Fc-tagged human Siglecs −2, −3 and −8 (all 50 μg/ml), Siglec-5 (2.5 μg/ml) as well as murine Siglec-F (2.5 μg/ml) were detected with AlexaFluor 647 conjugated anti-human IgG (7.5 μg/ml). Incubation with human galectin-1 (2 μg/ml) was followed by anti-human-galectin-1 from rat (5 μg/ml) and AlexaFluor 647 anti-rat IgG (2 μg/ml). Anti-Neu5Gc (1:200) binding was detected by anti-chicken IgY (1:1000 diluted) and AlexaFluor 647 anti-rabbit IgG (2 μg/ml). Negative controls for each applied lectin or antibody using solely the detection reagents (second and third incubation step where appropriate) were performed in parallel.

**Figure 4:**
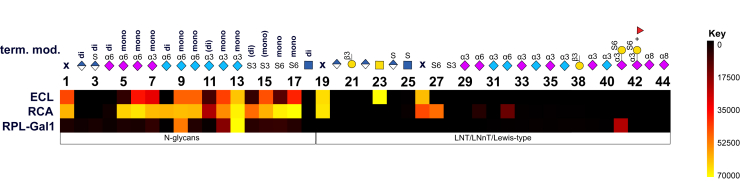
**Heat map displaying Gal-specific lectin binding to all structures**. The heat map was generated using the GLAD tool and represents the relative fluorescent units (RFU) normalized to 70,000 as the maximum value. Terminal modifications (of either one or both antennae) are shown. Unmodified β4Gal is indicated as x (no modification) while free terminal β3Gal is marked (compounds **21** and **38**). For complete structures please refer to Figure 2.

**Figure 5:**
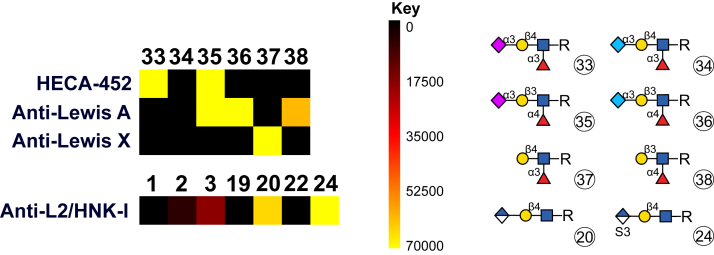
**Heat map displaying binding of Lewis- and HNK-1 epitope recognizing antibodies to selected specific structures**. The heat map was generated using the GLAD tool and represents the relative fluorescent units (RFU) normalized to 70,000 as the maximum value.

Slides were scanned with GenePix 4400A (Molecular Devices, LLC) in green (for fluorescein-conjugated SBA) and red light (AlexaFluor 647) using maximum gain and analyzed with the GenePix Version 7 software. Total fluorescence intensity values were extracted and evaluated (Microsoft Excel), subtracting the respective negative controls. Main statistical parameters including mean, standard deviation and coefficient of variation were calculated. Reproducibility in the sense of similar intensities on different fields or slides was verified in test cases (no significant difference in results even between different printing runs, Fig. S8). Data are depicted as heatmaps (Figure 3, Figure 4, Figure 5, summarized in Fig. S6) and graphs (total fluorescence intensity values shown as bar charts in Figs. S7–S9); the former generated using the GLAD tool (59) representing the relative fluorescent units (RFU) normalized to 70,000 as the maximum value.

## Data availability

All data are contained within the manuscript.

## Supporting information

This article contains supporting information with references (26, 32, 34, 46, 49, 54, 55, 60, 61, 62, 63, 64, 65, 66, 67, 68, 69, 70, 71, 72, 73, 74, 75, 76, 77, 78, 79, 80, 81, 82, 83, 84, 85).

## Conflict of interest

The authors declare that they have no conflicts of interest with the contents of this article.

## Supporting information

Supplement-results-methods-revised

DataTable-RFU

DataTable-qualitative
