# Supplementary material for: Enzymatic tailoring of anionic glycans for characterizing lectin and antibody specificities in a microarray format
Source: J Biol Chem. 2025 Nov 5;301(12):110915. doi: 10.1016/j.jbc.2025.110915 (PMC12720101; doi:10.1016/j.jbc.2025.110915)
Supplement: Supplement-results-methods-revised [file mmc1.pdf]

Enzymatic tailoring of anionic glycans for characterising lectin and antibody specificities in a microarray format

Lena Nuschy[1], Iain B.H. Wilson[1]

[1] Institut für Biochemie, Universität für Bodenkultur, Muthgasse 18, 1190, Vienna, Austria

Table of contents

Supplementary Results **S-2**

Validation of Microarray Functionality *S-2*

Recombinant Prokaryotic Lectin – Fuc1 .... *S-2*

GalNAc binding Lectins *S-2*

Ganglioside-type Glycans *S-4*

GAG Tetrasaccharides *S-5*

Summarizing Heat Map including Compounds 1-44 and GBPs *S-6*

Complementary Graphs with Total Fluorescence Intensities *S-7*

Supplementary Methods **S-11**

Summary of Glycosyl- and Sulphotransferases *S-11*

Expression of Neisseria meningitidis α2,3-Sialyltransferase (NmST3) and Photobacterium damsela α2,6-Sialyltransferase (PdST6) *S-12*

LNnT Substrate Purification *S-13*

Library Synthesis: RP-HPLC and MALDI TOF MS *S-14*

Further Information regarding the Glycan Array Analyses *S-57*

Supplementary Results

Validation of Microarray Functionality

All printed structures were probed with ConA (Man but also GlcNAc or Glc) as well as the biantennary N-type glycans (**1-18**) also with LCA (fucose) (Figure S1). Proper glycan immobilization to the NHS-modified glass slide could be verified. Although there was no binding of ConA to the Lewis-type saccharides, the presence of these was shown by binding to other proteins. Compounds **38-44** were at least bound by one designated ligand therefore correct printing was verified.

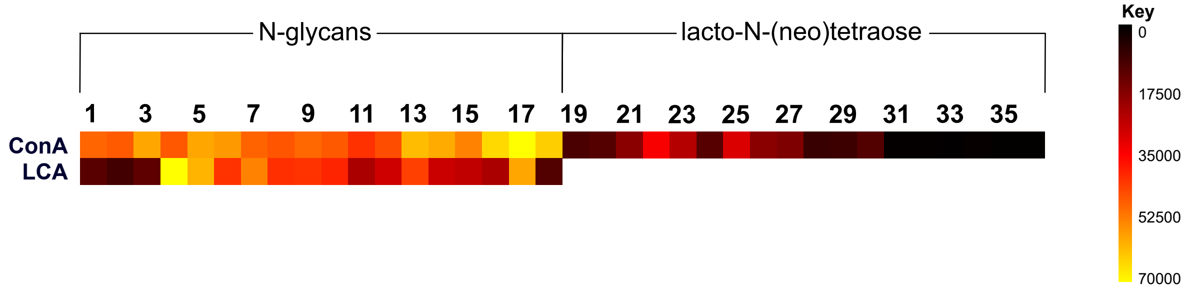

Figure S1: Heat map displaying binding of ConA and LCA to the library of printed structures. The heat map was generated using the GLAD tool and represents the relative fluorescent units (RFU) normalized to 70 000 as the maximum value.

Recombinant Prokaryotic Lectin – Fuc1

Commercial RPL-Fuc1 was applied to the library of enzymatically modified compounds. As expected, the protein was specific, and no signal was obtained in the absence of fucose (**19-32, 39-40, 42-44**) while all fucose containing structures were recognized (Figure S2). Amongst these however, subtle differences were noted. While α6 fucose was clearly preferred, signal intensities were decreased for α4-linkage (**35, 36, 38**) and the lowest for α3 fucose (**33, 34, 41**).

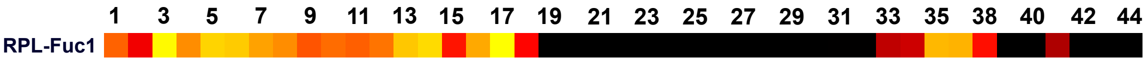

Figure S2: Heat map displaying binding of RPL-Fuc1 to the library of printed structures. The heat map was generated using the GLAD tool and represents the relative fluorescent units (RFU) normalized to 70 000 as the maximum value. For a color legend, please refer to Figure S1.

GalNAc binding Lectins

Not only selected anionic epitopes of medical interest, but also the LacdiNAc motif (Hirano *et al.*, 2022, *Biomolecules*, 12, 195) was analysed. Therefore, several GalNAc binding lectins (VVA, SBA, PNA, WGA, GSL-I and -II) were tested on compounds **19-30** accompanied with CLEC-10A and CLEC-14A (Figure S3). All plant lectins exclusively interacted with the LacdiNAc harboring compound **23** except SBA which further bound to, even though very weakly, 6-O-sulphated GlcNAc (**25**) and 6-O-sulphation of the internal Gal (**26**). CLEC-10A or macrophage galactose-type lectin (MGL), a type II transmembrane receptor playing a role in human T-cell immunity

and described to bind GalNAc (Hoober *et al.*, 2020, *Int. J. Mol. Sci.,* 21, 4818) was applied and interaction verified. Due to its reported recognition of heparin (Sandoval *et al.*, 2020*, J. Biol. Chem.*, 295, 2804-2821), CLEC-14A (also known as epidermal growth factor receptor 5), a type I transmembrane protein involved in angiogenesis, was suspected to recognize sulphated glycans. Indeed, we observed higher affinities for linear sulphate modified compounds (e.g. **24-26**, **28**), although not too intense. Nevertheless, it has to be noted that the background was high with definite unspecific binding.

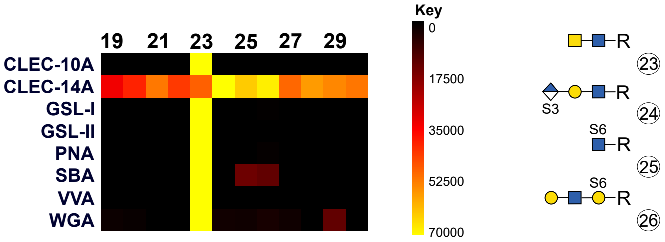

Figure S3: Heat map displaying binding of LacdiNAc or GalNAc specific lectins to linear enzymatically modified saccharides (**19-30**). The heat map was generated using the GLAD tool and represents the relative fluorescent units (RFU) normalized to 70 000 as the maximum value.

Ganglioside-type Glycans

In order to display a glycan library that comprises not only N-type and Lewis-type glycans, for example to further test Siglecs, we also included glycolipid oligosaccharides resembling ganglioside- and ceramide-type glycans (Figure S4A; in more detail: GA1, GM1b, GD1c, LacCer, GM3 and GD3, compounds **45-51**, respectively). Out of the applied Siglecs, only Siglec-5 and Siglec-F elicited detectable binding signals for α2,3Neu5Ac terminated compounds. Siglec-5 preferred GM3 while Siglec-F showed higher affinity towards GM1b (Figure S4B), a binding pattern which was also observed for the plant lectin WGA (consistent with binding data from Bunyatov *et al.*, 2025, *Angew. Chem. Int. Ed.* 64, e202415521). PNA on the other hand only recognized GA1 and tolerated no further sialic acid modification. Notably, α2,8Neu5Ac extensions were not bound by any of the tested human, mouse or plant lectins. As observed for the other relevant compounds, binding of SiaFind proteins was in general very weak with higher background noise and rather random binding pattern (Figure S4C); for example, SiaFind Pan2.0 binding to sialic acid modified lactose is even increased by one further α2,8Neu5Ac attached (**49** vs **50**), while it does not tolerate α2,8Neu5Ac extension of GM1b at all (**46** vs **47**).

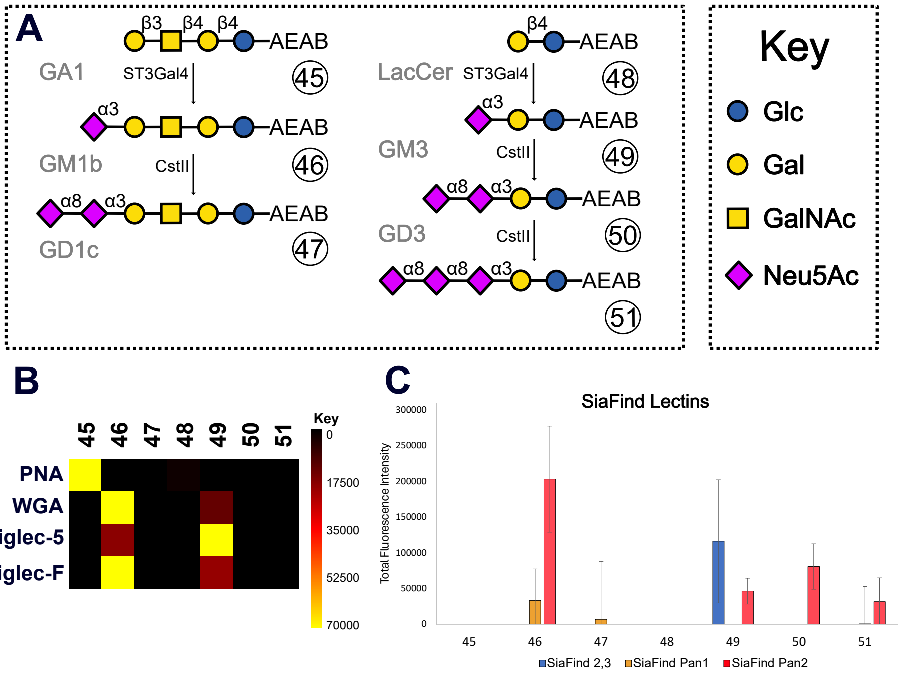

Figure S4: Glycan library extension by ganglioside- and ceramide-type oligosaccharides and corresponding lectin binding results. S4A: Composition of the 7 additional modified glycans (**45** is a precursor control, **46-51** have been enzymatically synthesized). The symbolic nomenclature for glycans is shown (Varki *et al.,* 2015, *Glycobiology*, 25, 1323-1324). S4B: Heatmap displaying detected binding using a set of relevant human, mouse and plant lectins. No binding signal was obtained for Siglec-3, -7, -8, -9 and -10, RPL-Gal1, -Gal4, -Sia1 as well as the plant lectins ConA, ECL, VVA, GSL-I B4, GSL-II, MAA-I and MAA-II, while very weak binding to 45 was detected with RCA. The heat map was generated using the GLAD tool and represents the relative fluorescent units (RFU) normalized to 70 000 as the maximum value. S4C: Total fluorescence intensities of the three sialic acid binding SiaFind proteins SiaFind α2,3, SiaFind Pan 1.0, SiaFind Pan2.0.

GAG Tetrasaccharides

Furthermore, two glycosaminoglycan (GAG) tetrasaccharides, chondroitin sulphate (CS) and heparan sulphate (HS) (structures are depicted in Figure S5A), were commercially available with a free reducing terminus. Compounds were AEAB-labelled, purified and finally printed and tested with relevant lectins and GBPs. In contrast to all other N-type and glycolipid-like glycans, after labelling and washing CS and HS were not subject to normal phase HPLC due to elution problems under the usual conditions, but instead purified on nonporous graphitized carbon (SupelClean ENVICarb; Sigma-Aldrich), a procedure previously used in the group (Hykollari *et al*., 2017, *Methods Mol. Biol.*, 1503, 167-184) for anionic glycans. Elution of GAGs was ensured with 40% (v/v) acetonitrile + 0.1% trifluoroacetic acid (v/v). Afterwards, HS was further purified via RP-HPLC using a Kinetex column, while CS was directly printed. Purity and compound composition was verified by MALDI-TOF MS and MS/MS as before.

While binding of heparan sulphate was solely detectable to brain-derived neurotrophic factor (BDNF), CS was recognized by RCA, BDNF, CLEC-10A and WFA (Figure S5B). Rogers and colleagues (Rogers *et al.,* 2011, *Proc. Natl. Acad. Sci.*, 108, 9747-9752) reported BDNF binding to CS-A (4-O-sulphated GalNAc) or CS-E (4- and 6-O-sulphated GalNAc) rather than CS-C (6-O-sulphated GalNAc), therefore we suggest at least a portion of our printed CS tetrasaccharide carries 4-O-sulphate on GalNAc (the exact composition is not defined by the supplier). Human MGL (CLEC-10A), confirmed to bind GalNAc (Figure S3), tolerates sulphate modification as well, while binding to GalNAc-specific plant lectins like VVA or PNA was diminished except for WFA, which is commonly used in neurological studies for visualization of perineuronal nets (PNNs), an extracellular matrix component containing chondroitin sulphate (Shinji *et al*., 2018, *Front. Integr. Neurosci.,* 12, 3). Due to the weak binding signal obtained for the CS tetrasaccharide used here, it is assumed that this chain of chondroitin sulphate is not the preferred binding partner for WFA, since the underlying glycan structures of CS harboring aggrecans in PNNs are not exactly defined and sulphate positions may be different or capping with unsaturated GlcA may inhibit binding. Interestingly, although no RCA binding to GalNAc terminated compound **23** (Figure 4 and Sphyris *et al*., 1995, *J. Biol. Chem.*, 270, 20292-20297) could be detected, binding signal was selectively observed for CS tetrasaccharide, correlating with previous studies reporting RCA binding also to 6-O-sulphated GalNAc terminating gangliosides (Liu *et al.,* 2024, *Int. J. Biol. Macromol.,* 272, 132624; Xu *et al.,* 2024, *Nat. Chem.*, 16, 881-892).

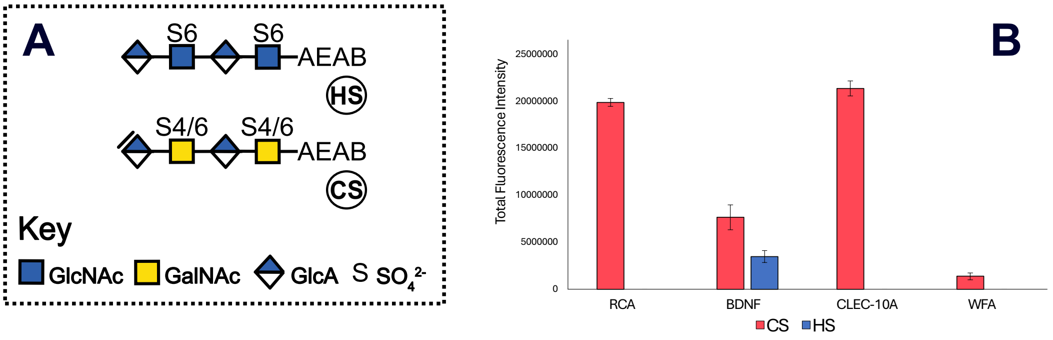

Figure S5: Structure of glycosaminoglycan tetrasaccharides and corresponding lectin binding results. S5A: Composition of chondroitin and heparan sulphate tetrasaccharides. The symbolic nomenclature for glycans is shown (Varki *et al.,* 2015, *Glycobiology*, 25, 1323-1324). Unsaturated terminal glucuronic acid is indicated by a double line. S5B: Total fluorescence intensities of the GBPs with detectable binding signals. No binding was observed for CLEC-14A, ConA, PNA, VVA, GSL-I and -II and WGA.

Summarizing Heat Map including Compounds 1-44 and GBPs

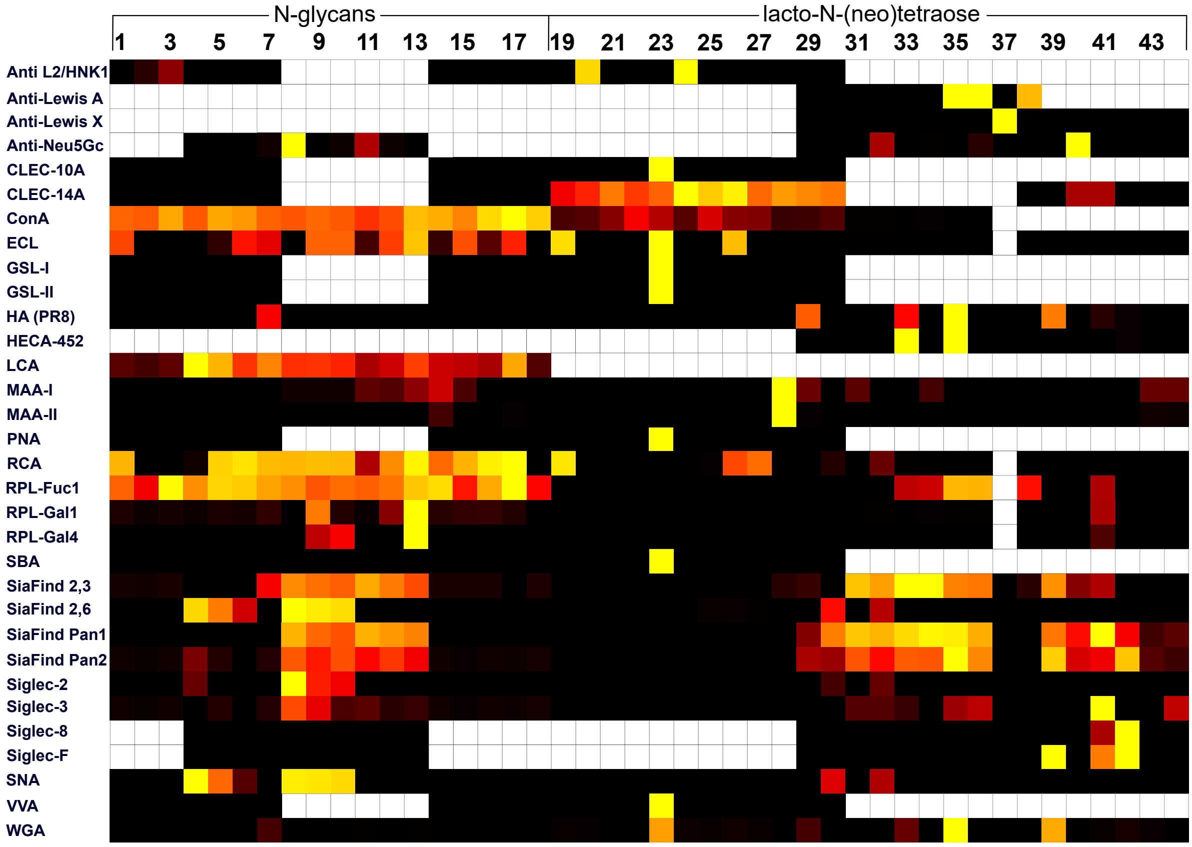

Figure S6: Complete heat map displaying binding of all available lectins and antibodies to the synthesized glycan library excluding ganglioside-like glycans. The heat map was generated using the GLAD tool and represents the relative fluorescent units (RFU) normalized to 70 000 as the maximum value. Glycan-GBP combinations that were not tested (e.g. due to no indication of epitope recognition) are depicted as white squares. For a color legend, please refer to Figure S1.

Complementary Graphs with Total Fluorescence Intensities

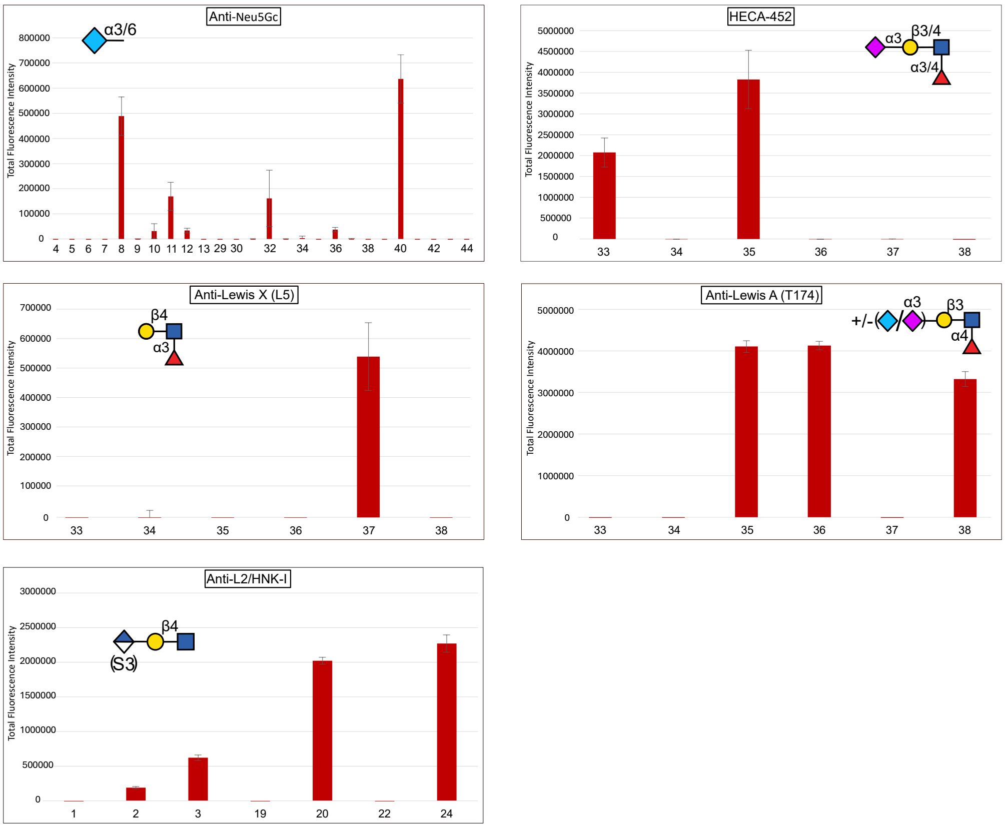

Figure S7: Individual graphs depicting total fluorescence intensities of all antibodies used in the microarray study. Relevant tested compounds are numbered.

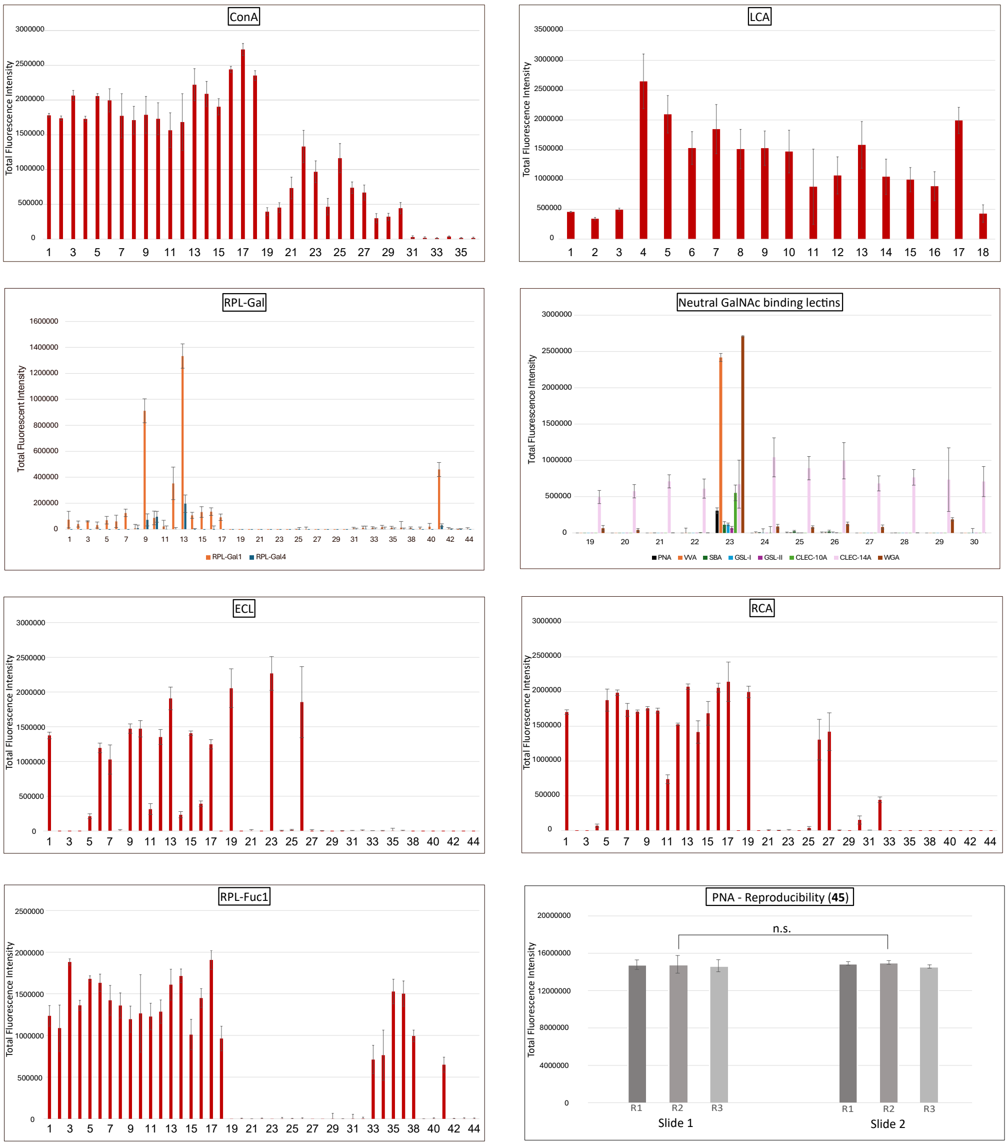

Figure S8: Individual graphs depicting total fluorescence intensities of all neutral glycan binding lectins used in the microarray study. Relevant tested compounds are numbered. Compound 37 was only used as a positive control for the anti-Le^{A} antibody and therefore excluded from most other assays. A reproducibility experiment was performed (lower right panel) with compound **45**. Two separate slides (three parallel printing runs R1-R3 including 5 replicates each) were probed with PNA and results compared (one-way ANOVA, α<0.05). A P-value of 0.897 was calculated, indicating no significant difference between measurements and fair reproducibility.

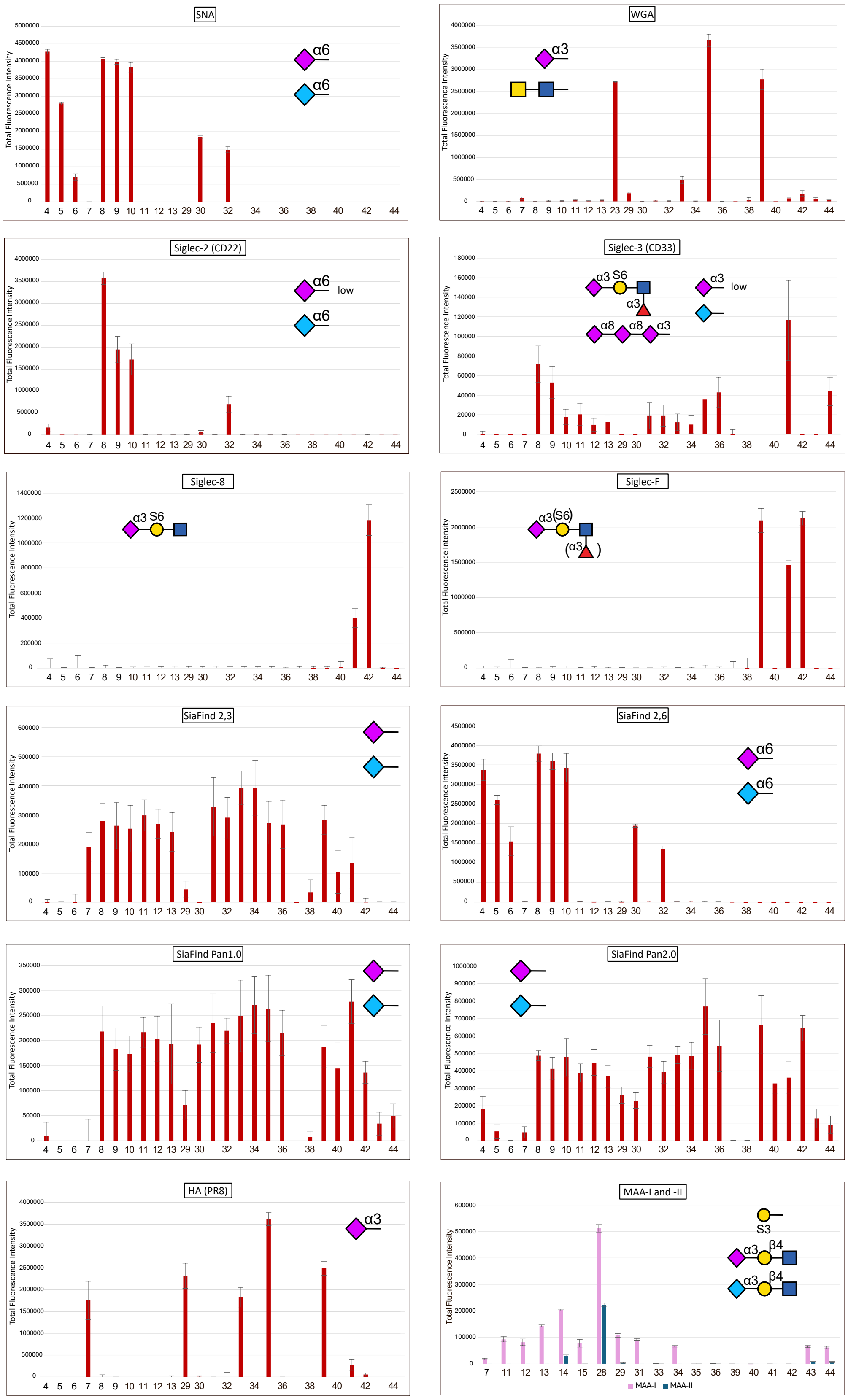

Figure S9: Individual graphs depicting total fluorescence intensities of all designated sialic acid binding lectins used in the microarray study. Relevant tested compounds are numbered and the most prominent epitopes depicted.

Table S1: Comparison of lectin specificities reported by the vendor (VectorLabs) and results obtained from the tested compounds. The table represents only specificities defined by the vendor and does not reflect lectin binding partners previously determined by other research groups; for more details regarding findings from the literature please refer to the main text. A similar comparison was done within a study using machine learning by Bojar and colleagues (Bojar *et al.,* 2022, *ACS Chem. Biol.*, 17, 2993-3012); however, certain structures (especially GlcA and Neu5Gc containing ones) and specificities from our current work were not included in that previous study. Where specificities reported by VectorLabs could be verified, letters are marked in bold. Glycoepitopes not depicted in our library are indicated with asterisk. Observations for oligomannosidic glycans were made during earlier glycan microarrays of the group.

Lectin	Specificity (Vectorlabs)	Specificity (observed)
ConA	**αMan, αGlc***	Oligomannose and biantennary
ECL	**Galβ4GlcNAc**	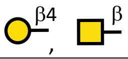
GSL-I-B_4	αGal*	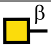
GSL-II	**α* or βGlcNAc**	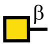
LCA	αMan, αGlc*	N-glycans with core Fuc
MAA-I	Galβ4GlcNAc	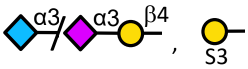
MAA-II	Neu5Acα3Galβ3GalNAc	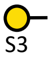
PNA	**Galβ3GalNAc**	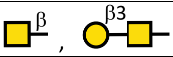
RCA	**Gal**	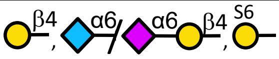
SNA	**Neu5Acα6Gal/GalNAc**	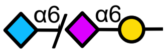
SBA	**α*>βGalNAc**	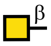
VVA	**GalNAc**	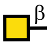
WGA	**GlcNAc**	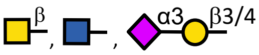

Supplementary Methods

Summary of Glycosyl- and Sulphotransferases

Table S2: Overview of enzymes used for glycan library synthesis and their substrate preferences. References of the first enzyme description are indicated and catalog numbers of purchased enzymes provided; prices are in a range of 300-650€ (350-760 USD). Typically, 20-100 nmol of substrate were used per reaction.

	Enzyme	Supplier Catalog#	Substrates*	Structures not accepted	Linkage formed	Notes	Ref
Glycosidases	**Anβ4Gal EC 3.2.1.23**	homemade	terminal β4-Gal			efficient in 4h	[72]
	**JBHex EC 3.2.1.52**	Sigma-Aldrich A2264	terminal GlcNAc			efficient in 4h	[73]
Sialyltransferases	**NmST3 EC 2.4.3.6**	homemade	LNnT biantennary N-glycans	Lewis-type glycans	α2,3	lower activity on biantennary N-glycans	[54]
	**PdST6 EC 2.4.3.1**	homemade	LNnT/LNT biantennary N-glycans		α2,6	high efficiency	[55]
	**ST3Gal4 EC 2.4.3.6**	R&D Systems 10496-GT	LNnT/LNT biantennary N-glycans	Lewis-type glycans sulphated Gal	α2,3	prefers LNT over LNnT	[75]
	**PmST3 EC 2.4.3.6**	Sigma-Aldrich S1951	Lactose	Lewis-type glycans sulphated Gal	α2,3	very low efficiency on LNnT & biantennary N-glycans	[76]
	**CstII EC 2.4.3.8**	ChemilyBio EN01003	sialylated Galβ1-3/4GlcNAc/GalNAc		α2,8	very efficient transferring one or two Sia	[74]
Fucosyltransferases	**FUT3 EC 2.4.1.65**	R&D Systems 4950-GT	(sulphated/sialylated) LNnT/LNT		α3/4	prefers LNT over LNnT fucosylates GlcNAc and Glc	[77] [85]
	**CeFUT6 EC 2.4.1.-**	homemade	LNnT	sialylated LNnT	α3		[78]
	**HpFUT3 EC 2.4.1.-**	ChemilyBio EN01020		sialylated LNnT	α3		
Galactosyltransferase	**B3GalT5 EC 2.4.1.-**	R&D Systems 10555-GT	terminal GlcNAc		β3	high efficiency	[79]
N-acetyl-galactosaminyl-transferase	**GalT1 (Y289L)**	ThermoFisher C33368	terminal GlcNAc		β4	high efficiency at 4°C	[80]
Glucuronyltransferase	**B3GAT1 EC 2.4.1.135**	R&D Systems 8560-GT	LNnT/LNT biantennary N-glycans		β3	prefers LNnT, low activity on LNT	[81]
Sulphotransferases	**CHST1 EC 2.8.2.-**	R&D Systems 5316-ST	(sialylated) LNnT biantennary N-glycans		6-O on Gal	medium effficiency	[82]
	**CHST2 EC 2.8.2.-**	R&D Systems 5107-ST	terminal GlcNAc	LNnT	6-O on GlcNAc	low efficiency	[83]
	**CHST10 EC 2.8.2.-**	R&D Systems 6140-ST	glucuronylated biantennary N-glycans		3-O on GlcA	high efficiency	[46]
	**GAL3ST2 EC 2.8.2.-**	R&D Systems 7719-ST	LNnT biantennary N-glycans		3-O on Gal	medium efficiency	[84]

*tested in this array study

Expression of Neisseria meningitidis α2,3-sialyltransferase (NmST3) and Photobacterium damsela α2,6-sialyltransferase (PdST6)

Glycerol stocks of BL21 *E. coli* transformed with plasmids containing His-tag conjugated NmST3 and PdST6, respectively, were used for inoculation of 2 mL half-salt Luria-Bertani (LB) medium supplemented with 50 µg/mL kanamycin and cultured at 37°C for 16 h. 50 mL (NmST3) and 30 mL (PdST6) cultures were inoculated and grown until OD_{600} of 0.6 before induction with 0.5 mM IPTG. NmST3 was expressed at 16°C for 16 h, PdST6 at 25°C for 5 h. Cells were harvested and resuspended in lysis buffer (50 mM potassium phosphate pH 7.8, 400 mM NaCl, 100 mM KCl, 10% glycerol, 0.5% Triton X-100 and 100 mM imidazole) complemented with three freeze-thaw cycles in liquid nitrogen and 42°C respectively. Enzymes were purified via a HisTrap HP 1 mL column (Cytiva, Marlborough, MA, US) using an ÄKTA Go purification system. Fractions were analyzed by Western Blot and those containing the desired proteins pooled.

LNnT Substrate Purification

Normal-phase HPLC of AEAB-labeled LNnT (also applying to LNT and aGM1) as a first purification step (**compound 19**). Verification was achieved by MALDI-TOF MS. Samples were spotted using 6-aza-2-thiothymine (ATT) as matrix and analyzed in positive ion mode MS.

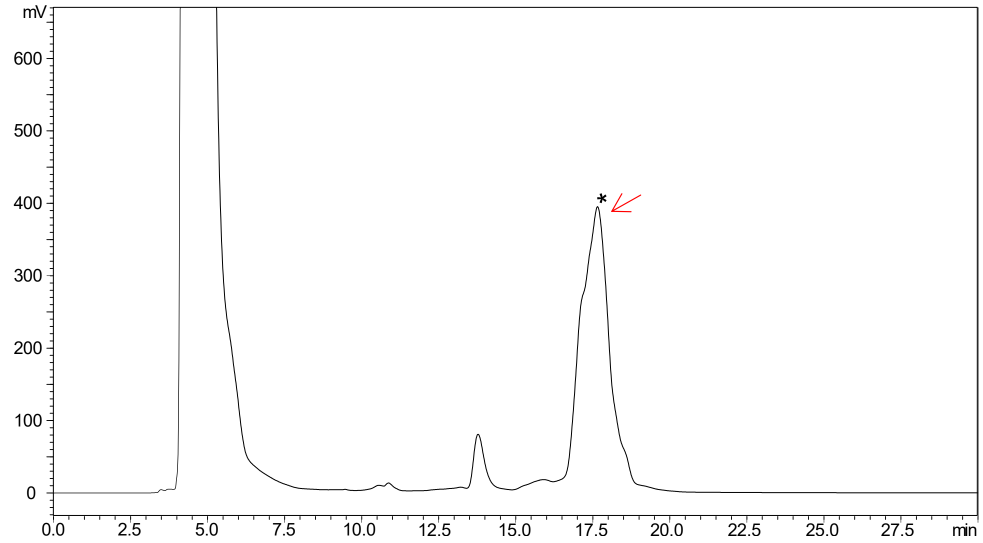

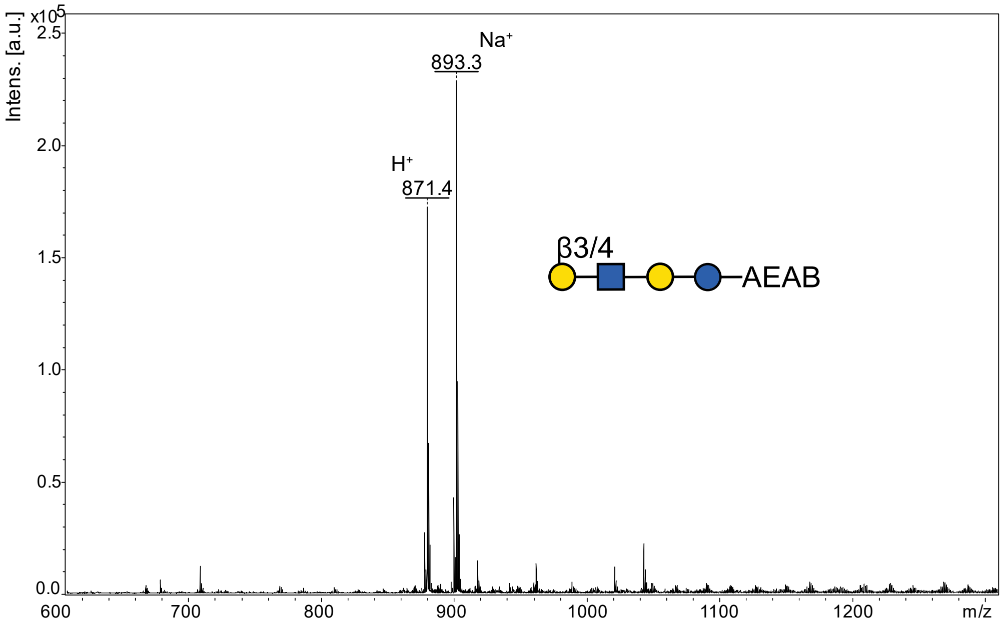

Library Synthesis: RP-HPLC and MALDI TOF MS

All compounds except **19** were purified in one or even two RP-HPLC runs using a Kinetex column and a two solvent gradient with buffer A (0.1 M ammonium acetate buffer pH 4) and buffer B (30% MeOH). Conditions for **1-18**: 0-2 min, 15% B; 2-5 min, 25% B; 5-10 min, 30% B; 10-25 min 50% B; 25-35 min, 95% B; 35-40 min, 95% B; 40-46 min, 0% B; 46-50 min, 0% B.
Conditions for **20-30**, **32-33**, **35-36, 38-51 and HS**: 0-2 min, 15% B; 2-5 min, 25% B; 5-10 min, 30% B; 10-20 min 50% B; 20-35 min, 95% B; 35-40 min 95% B; 40-46 min, 0% B; 46-50 min, 0% B.
Conditions for **31**, **34**, **37**: 0-2 min, 15% B; 2-5 min, 25% B; 5-10 min, 37% B; 10-20 min 47% B; 20-35 min, 95% B; 35-40 min 95% B; 40-46 min, 0% B; 46-50 min, 0% B.

Samples were spotted using 6-aza-2-thiothymine (ATT) as matrix and analyzed in positive and negative ion mode MALDI-TOF MS and MS/MS (spectra shown where appropriate). The product peak is indicated by a red arrow with asterisk, the elution position of the respective substrate by a light blue arrow.

Compound 1: Biantennary core-fucosylated GalGal (commercial compound), purified by RP-HPLC.

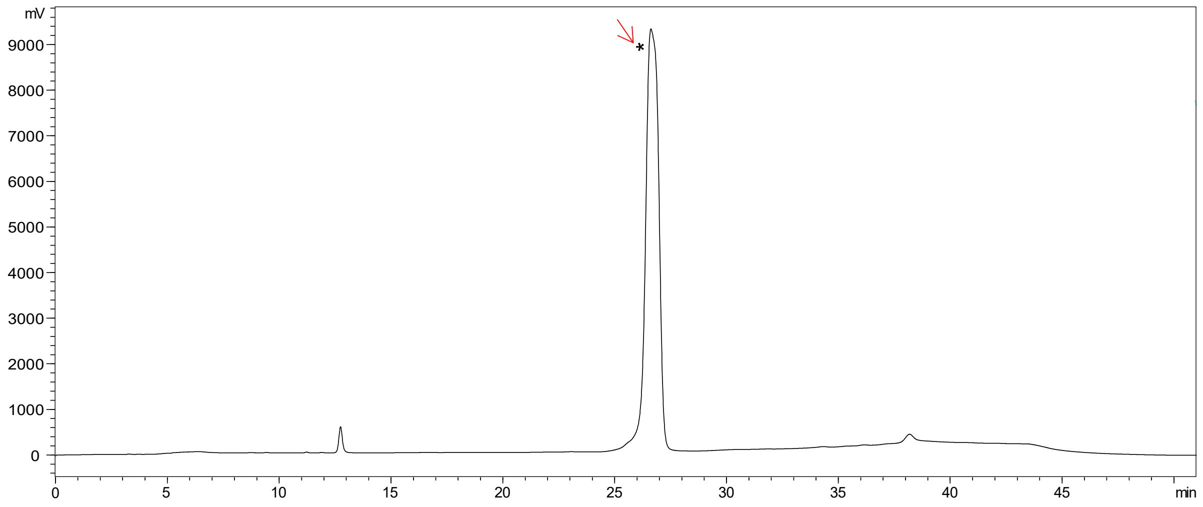

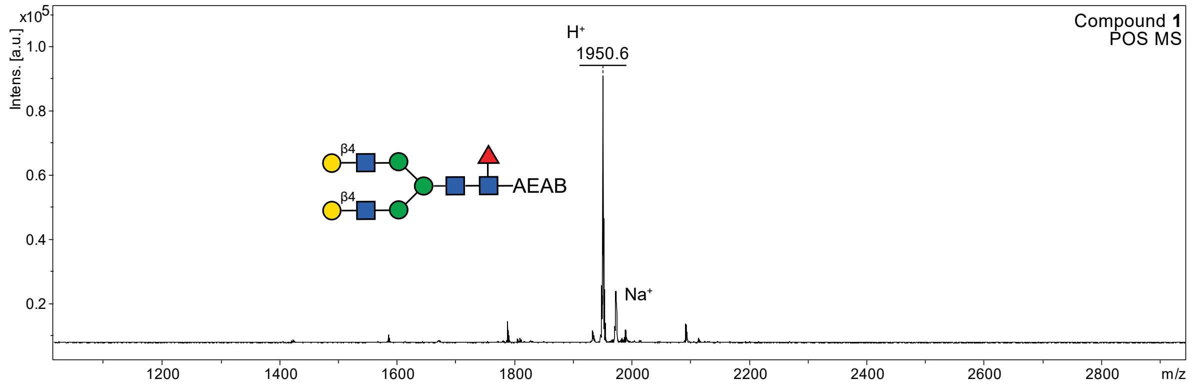

Compound 2: Biantennary core-fucosylated GalGal, modified with terminal GlcA (+B3GAT1 glucuronyltransferase). Substrate conversion was complete.

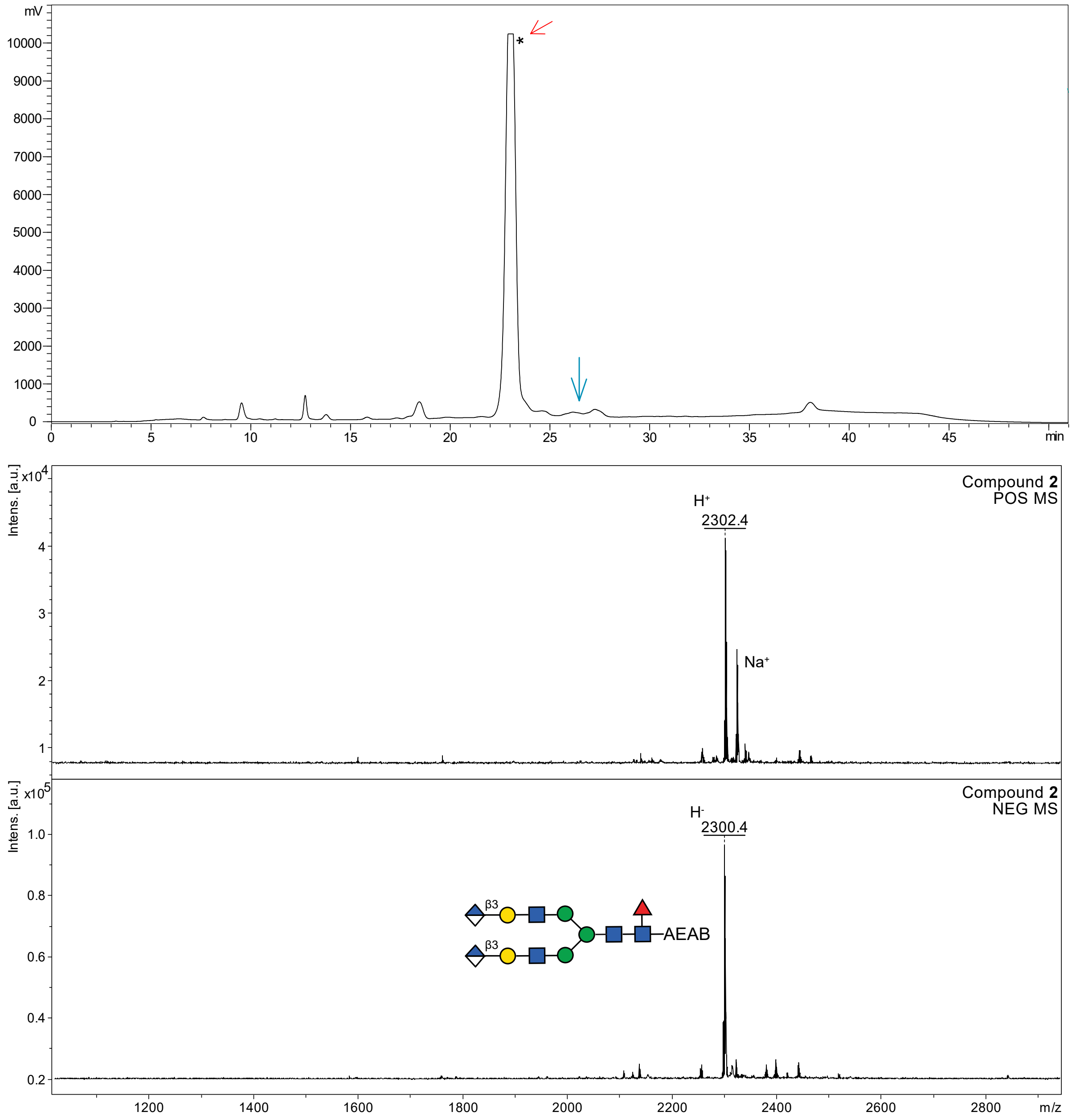

Compound 3: Biantennary core-fucosylated GalGal, modified with sulphated terminal GlcA (+CHST10 sulphotransferase). MS-Data from determination of the sulphate position on either of the glucuronic acid residues using a glucuronidase combined with β4-galactosidase and JBHex however, were inconclusive. Around two third of the compound seem to be mono-sulphated, although in-source fragmentation cannot be fully excluded.

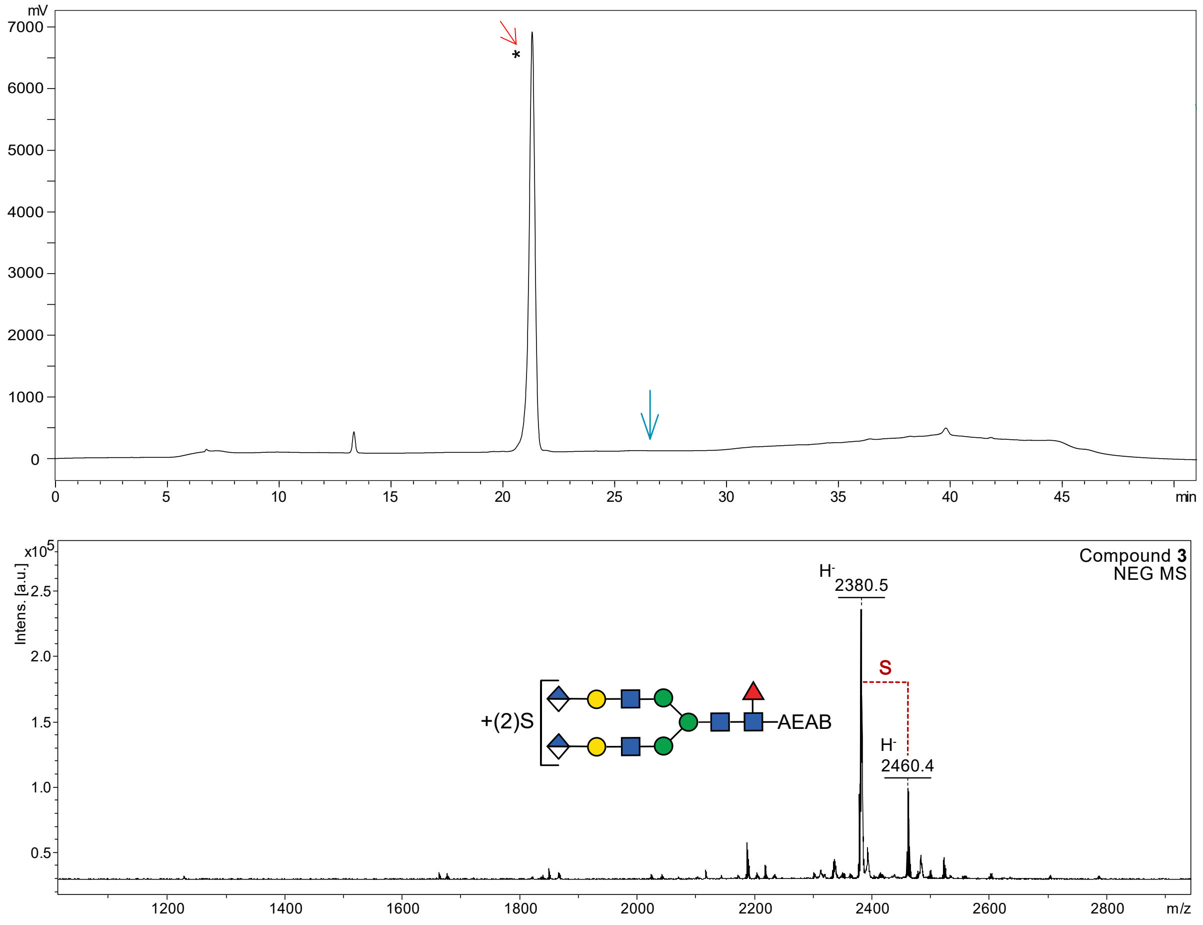

Compound 4: Biantennary core-fucosylated GalGal, modified with terminal α-6 linked Neu5Ac residues on both antennae (+PdST6 sialyltransferase). The *m/z* Δ55 (sometimes Δ53 in negative ion mode) was frequently observed for sialic acids and is considered a sample variable loss.

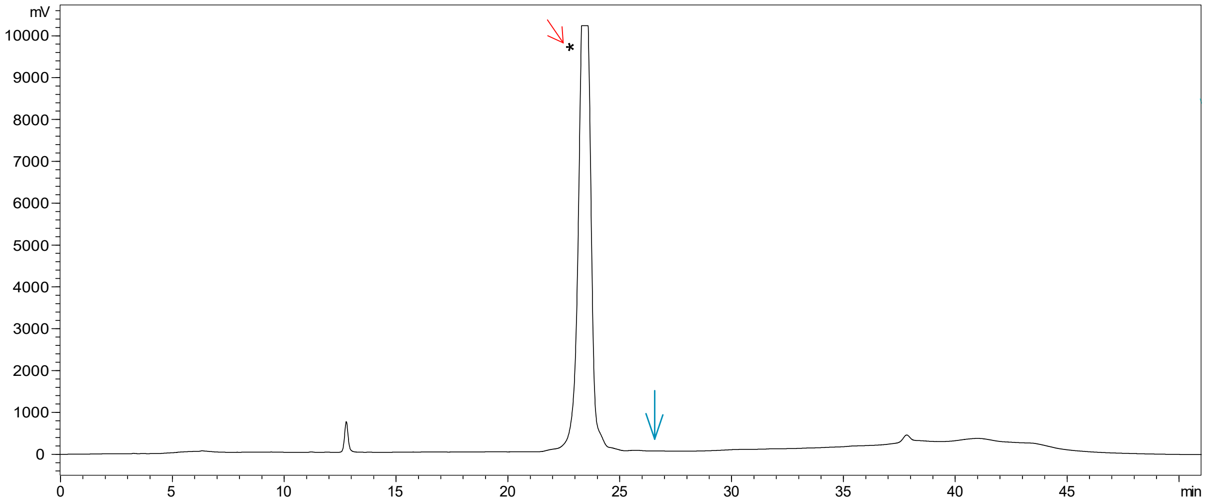

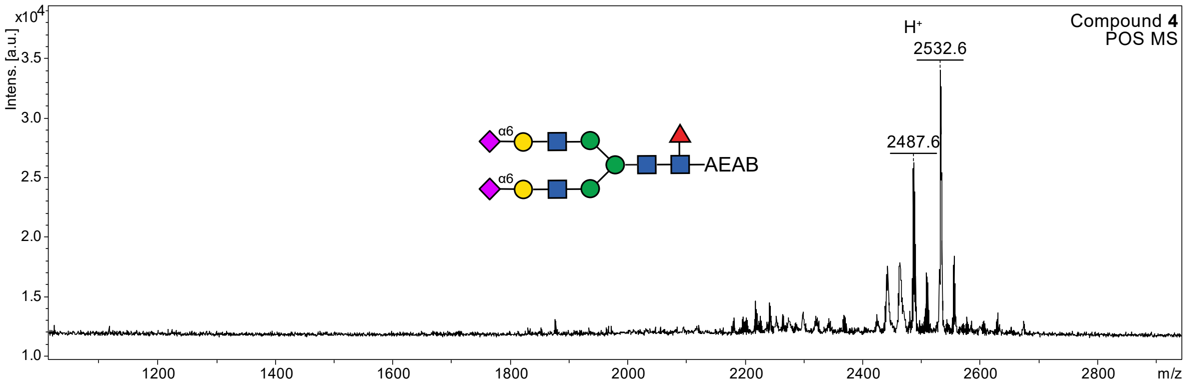

Compound 5: Biantennary core-fucosylated GalGal, modified with one terminal α-6 linked Neu5Ac residue (+PdST6) on the lower antenna. Position of the Neu5Ac was determined by digestion using a combination of β4-galactosidase, JBHex and JBM, which preferentially cleaves α-3 linkage of Man. The compound was resistant to JBM (the loss of one N-acetylhexosamine and one hexose is indicated as -NH). Comparing chromatograms, transfer of Neu5Ac to the lower antenna was more efficient (compound **6** is a mixture of mono-sialylated and non-sialylated substrate), supporting previous reports, that 2,6 sialyltransferases may prefer α-3 linkage of Man (Joziasse *et al.*, 1985, *J. Biol. Chem.*, 260, 714-719).

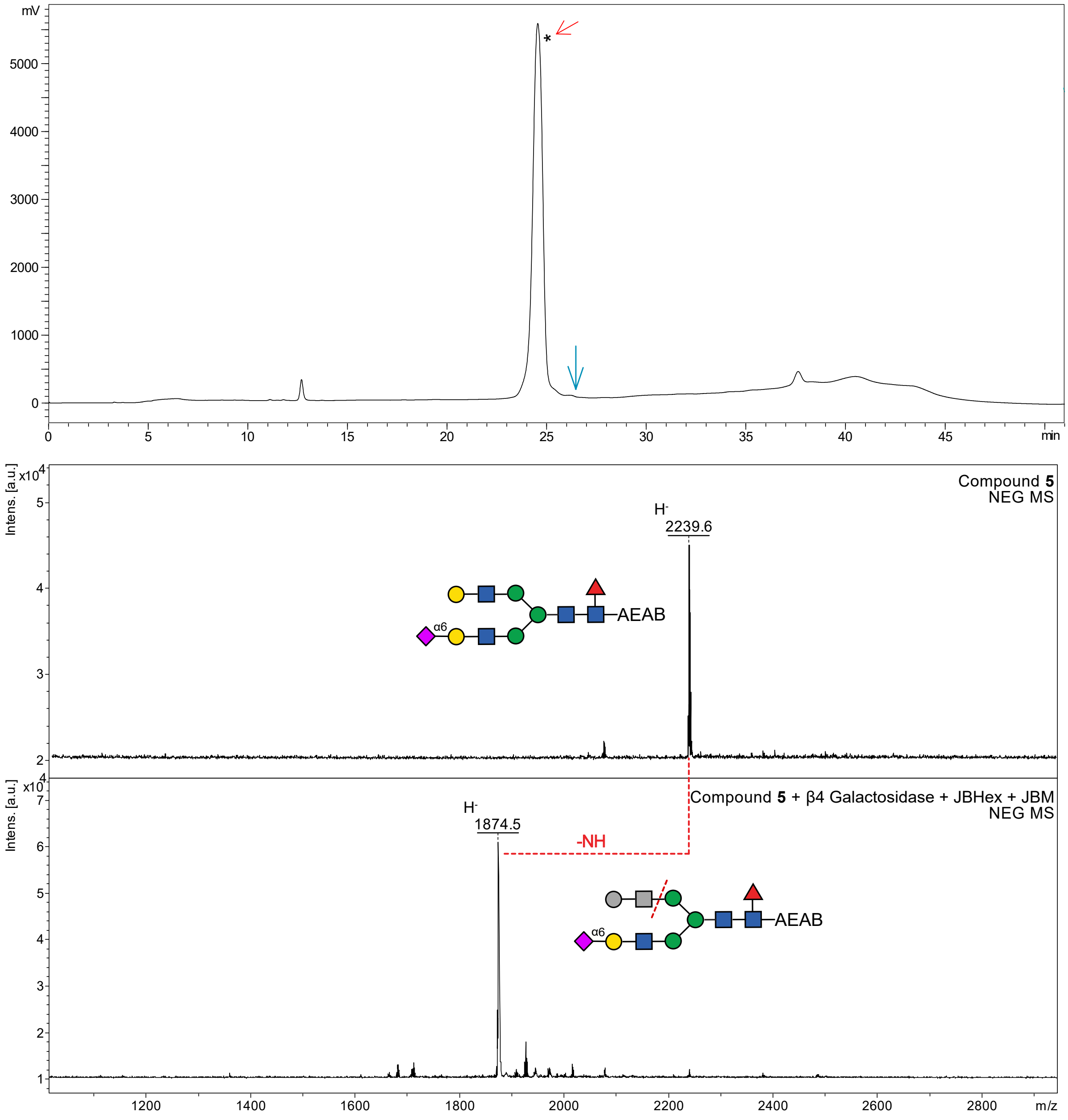

Compound 6: Biantennary core-fucosylated GalGal, partly modified with one terminal α-6 linked Neu5Ac residue (+PdST6) on the upper antenna. The structures were inseparable by RP-HPLC. Position of the Neu5Ac was determined by digestion using a combination of β4-galactosidase, JBHex and JBM, which preferentially cleaves α-3 linkage of Man. The compound was sensitive to all applied glycosidases.

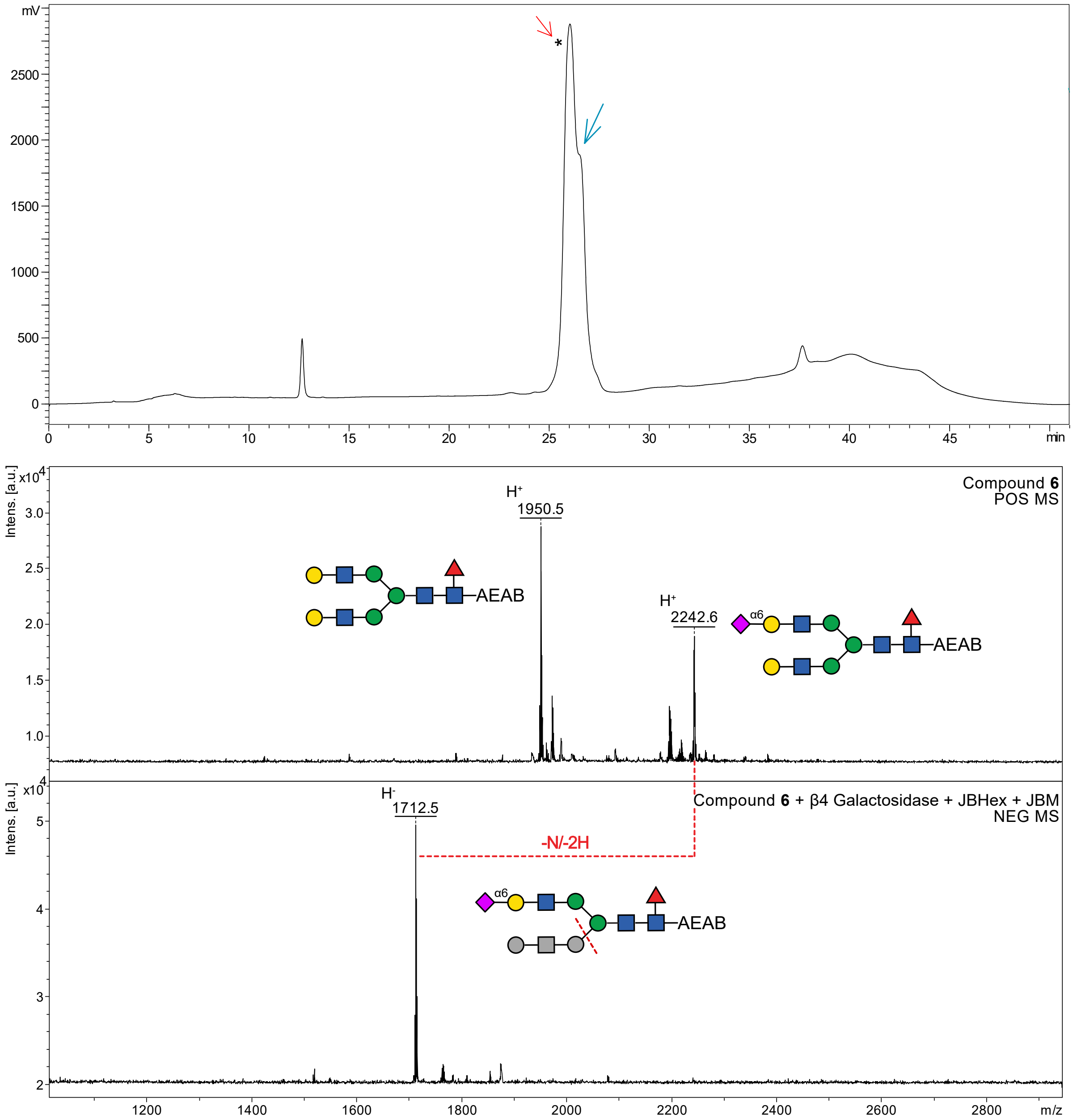

Compound 7: Biantennary core-fucosylated GalGal, modified with one terminal α-3 linked Neu5Ac residue (+NmST3 sialyltransferase) on the upper antenna. Position of the Neu5Ac was determined by glycosidase digestion. Lower arm α-3 linked Neu5Ac modification by NmST3 was highly inefficient, therefore not enough (double-sialylated) product for printing could be generated. Although the Manα1-6Man arm is preferred by recombinant rat sialyltransferase, no branch specificity for NmST3 was observed in a previous study (Fukae *et al.*, 2004, *Glycoconj. J.*, 21, 243-250).

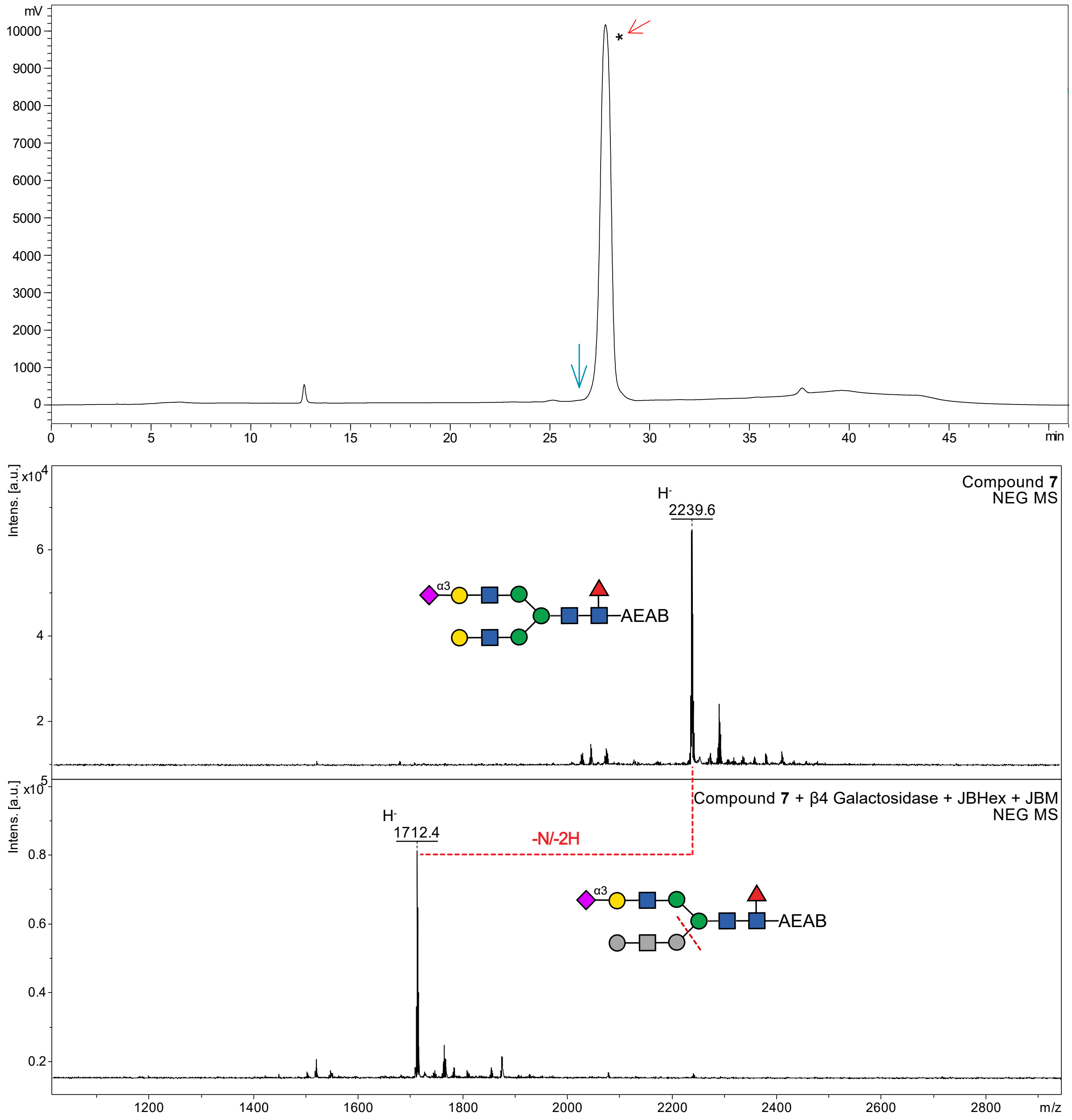

Compound 8 (9+10): Biantennary core-fucosylated GalGal, modified with terminal α-6 linked Neu5Gc residues on both antennae (+PdST6). Since sufficient amounts of products could be generated with the first assay and mass spectra indicated good separation and fair compound purity, a second round of HPLC was not performed. The α-3 Man-linkage preference was not noted unlike with CMP-Neu5Ac as donor, rather equal amounts of all three compounds were produced.

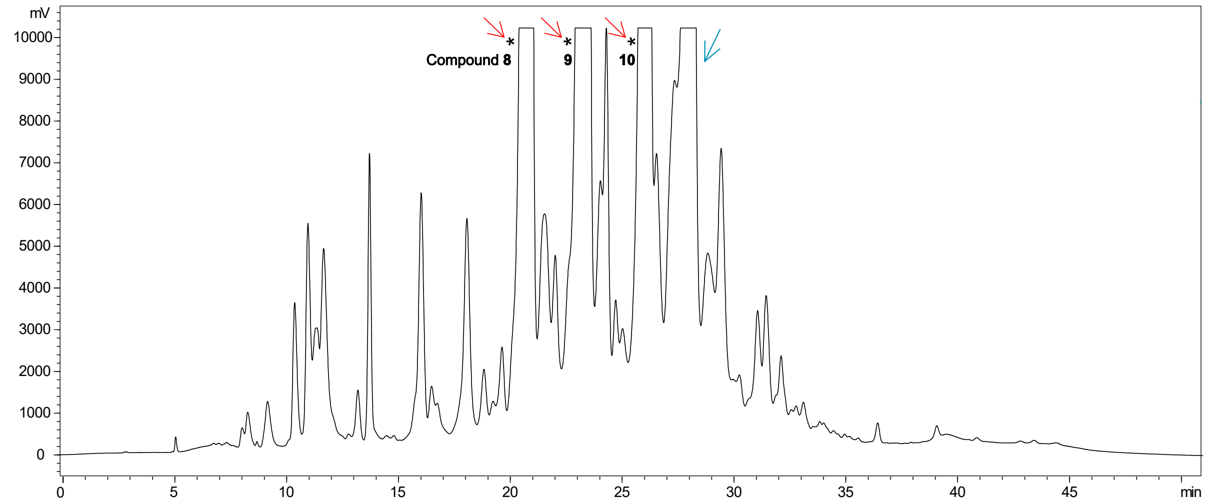

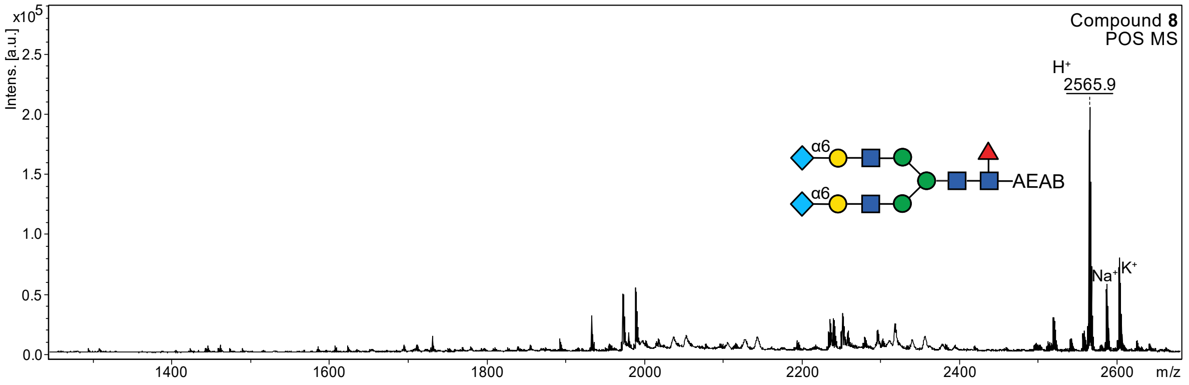

Compound 9 and 10: Biantennary core-fucosylated GalGal, modified with one terminal α-6 linked Neu5Gc residue (+PdST6) on either antenna. Position of the Neu5Gc was determined by digestion using a combination of β4-galactosidase, JBHex and JBM, which preferentially cleaves α-3 linkage of Man. Sensitivities are indicated.

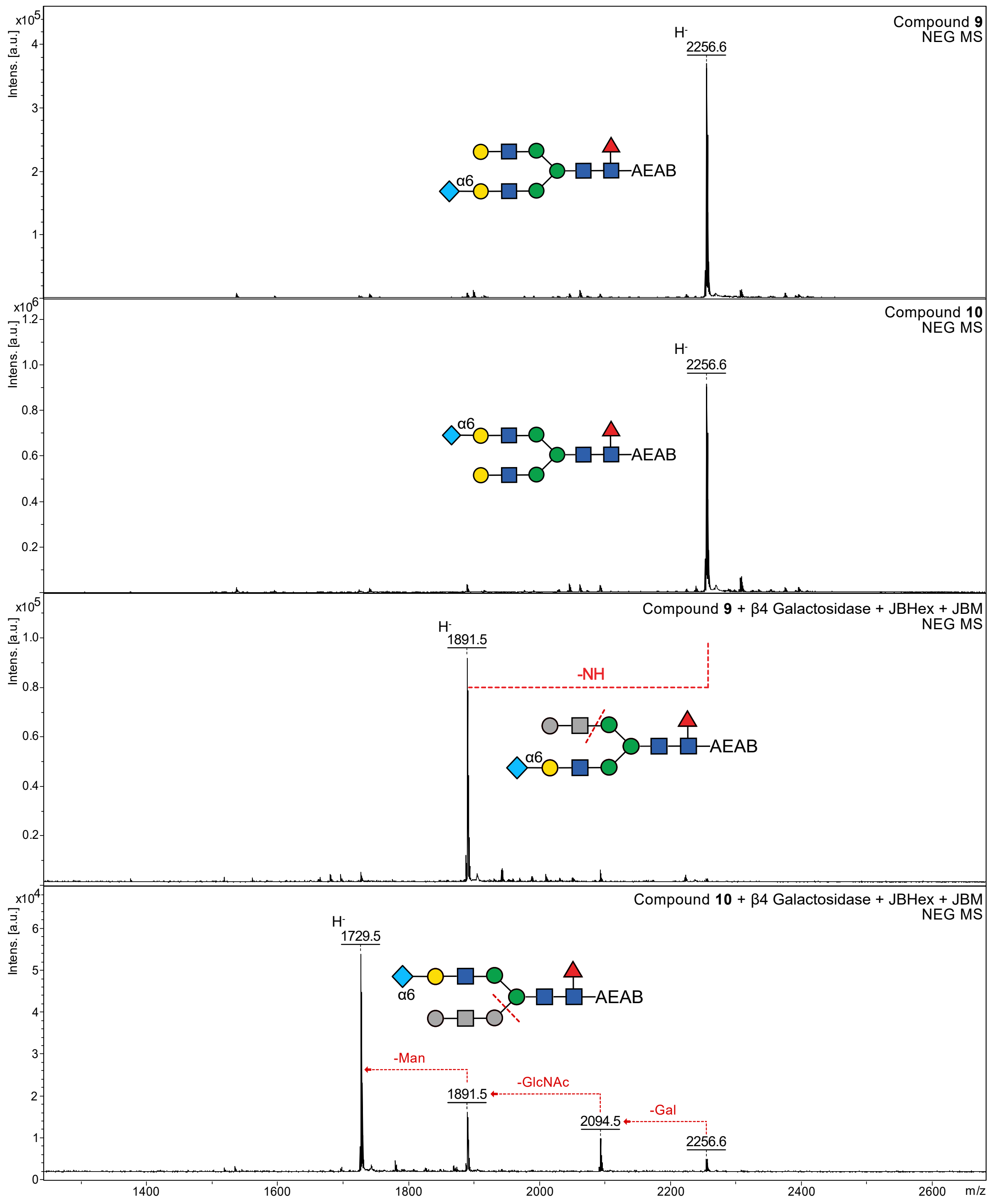

Compound 11 (12+13): Biantennary core-fucosylated GalGal, modified with terminal α-3 linked Neu5Gc residues mainly on both antennae (+ST3GAL4 sialyltransferase). As for compounds **8-10**, re-HPLC was dispensed. Since mono-sialylated product was also detected in the MS-spectrum, a combination of β4-galactosidase, JBHex and JBM was applied. The compound was insensitive to the mannosidase.

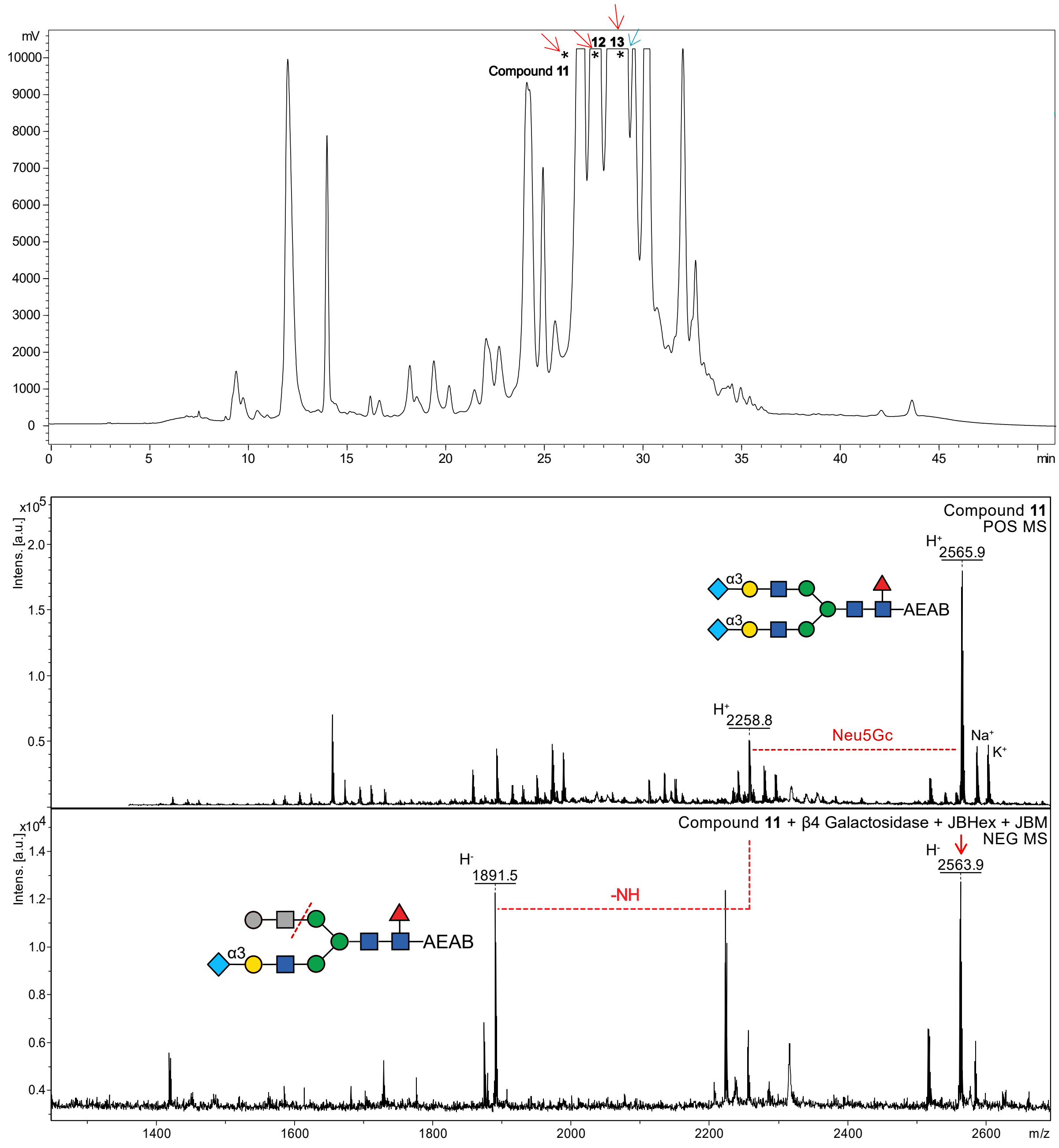

Compound 12 and 13: Biantennary core-fucosylated GalGal, modified with one terminal α-3 linked Neu5Gc residue (+ST3GAL4) on either antenna. Position of the Neu5Gc was determined by glycosidase digestion. Sensitivities are shown. Compound **13** is a mixture with unmodified substrate (indicated by the MS negative ion mode adduct mass peak). ST3GAL4, in contrast to NmST3, could transfer to both antennae although a slight tendency for the α-3 linked Man arm was noticed.

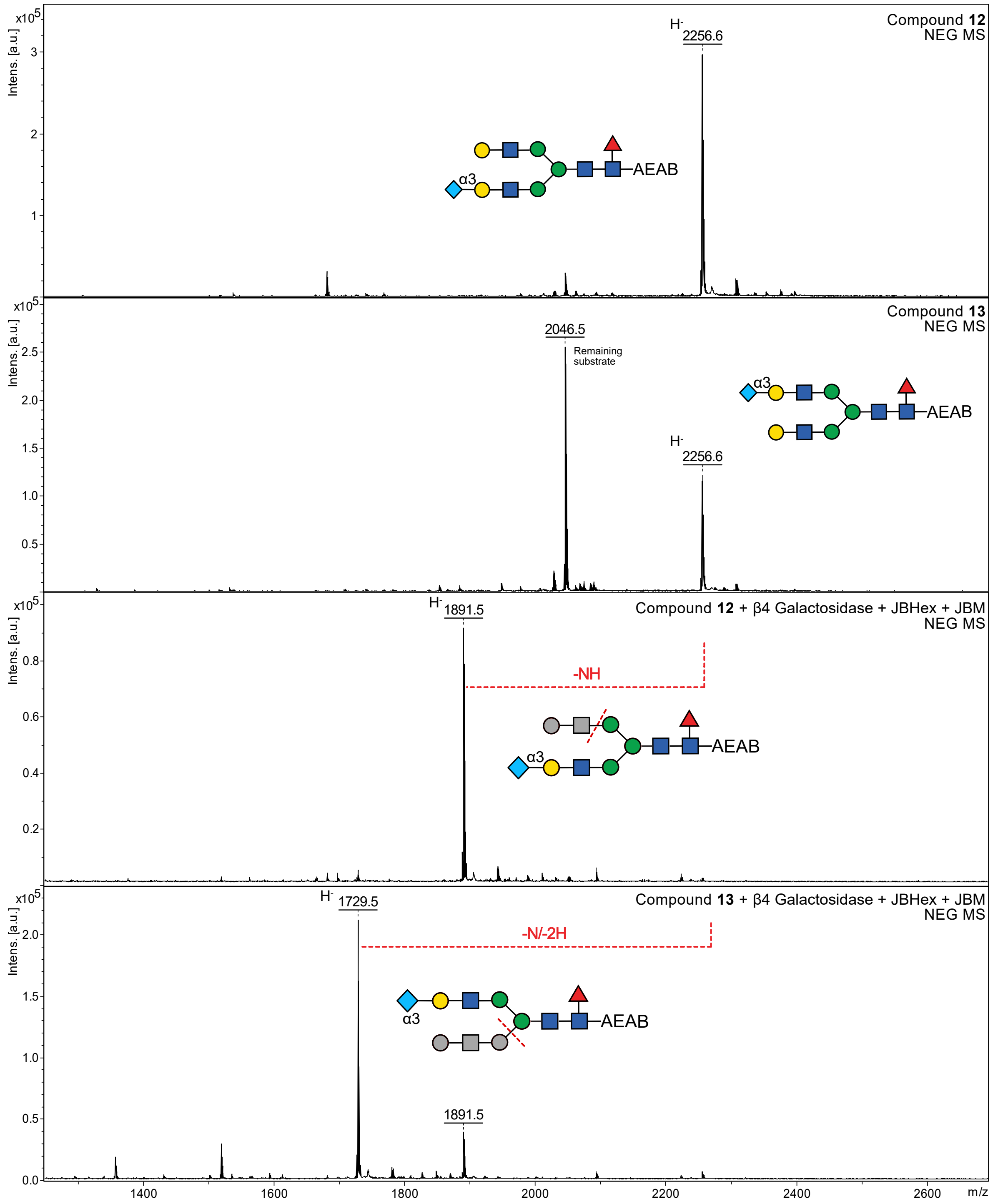

Compound 14: Biantennary core-fucosylated GalGal, with either one or both (*m/z* 2130.4) terminal Gal residues carrying 3-sulphate (+GAL3ST2 sulphotransferase). Main sulphate position was determined by digestion using a combination of β4-galactosidase, JBHex and JBM, which preferentially cleaves α-3 linkage of Man. The compound was mostly resistant to JBM, indicating the predominance of 3-sulphation of Gal residue on the lower antenna. The unmodified substrate could be separated by HPLC, however the fraction containing sulphated isomers was inseparable. While others claim high antennary preference of GAL3ST2 for the Manα1-6Man arm (Huang *et al.*, 2024, *JACS Au*, 4, 2966-2978), here it is rather inconclusive, although transfer to both antennae was incomplete.

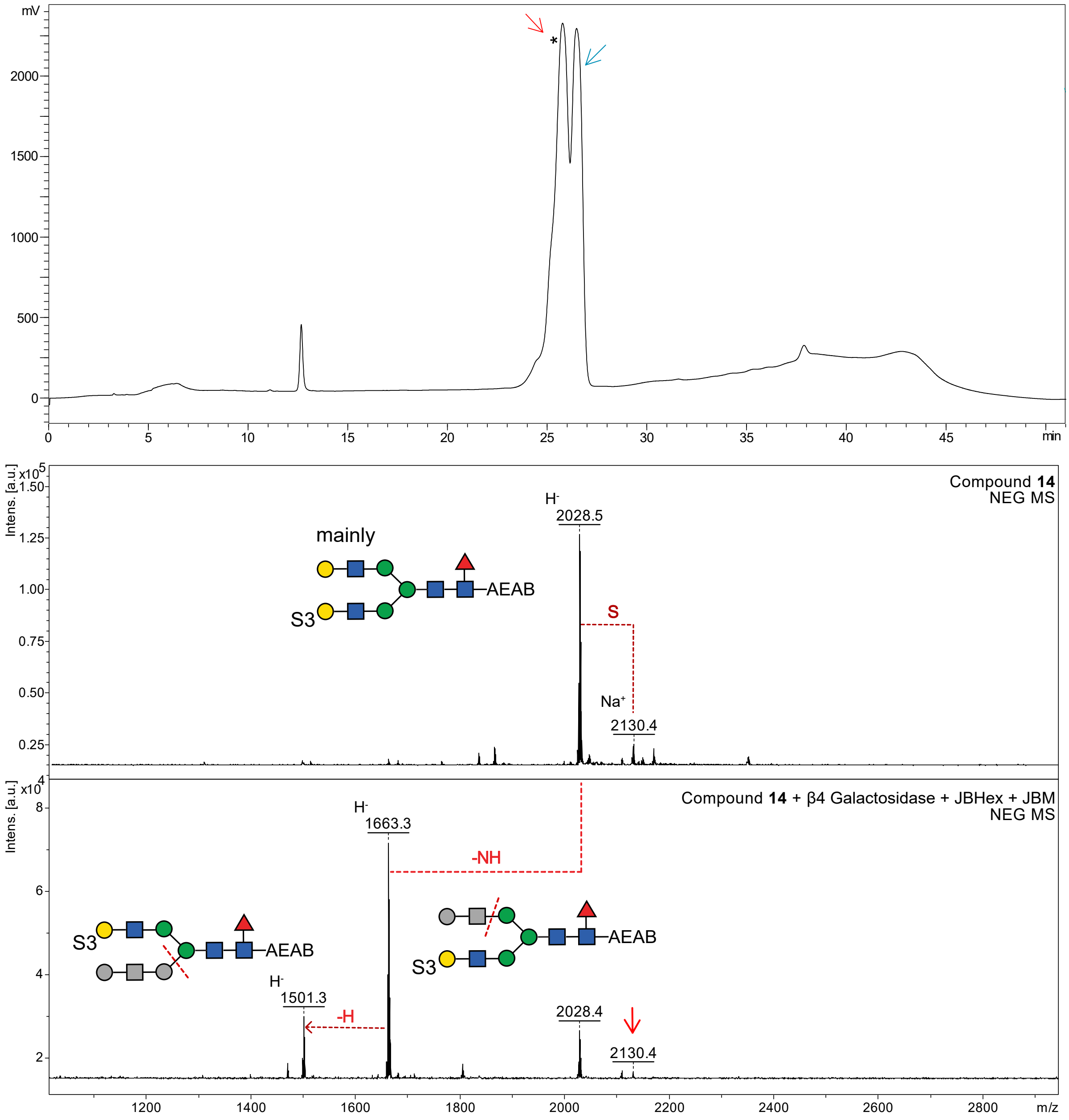

Compound 15: Biantennary core-fucosylated GalGal, partly with one 3-sulphated Gal residue (+GAL3ST2) on the upper arm (substrate indicated by the MS negative ion mode adduct mass peak). Position of the 3-sulphate, if present was determined by glycosidase digestion. The compound was mostly sensitive to JBM, confirming the predominance of 3-sulphation of Gal residue on the upper antenna. The mixture of substances was inseparable by HPLC.

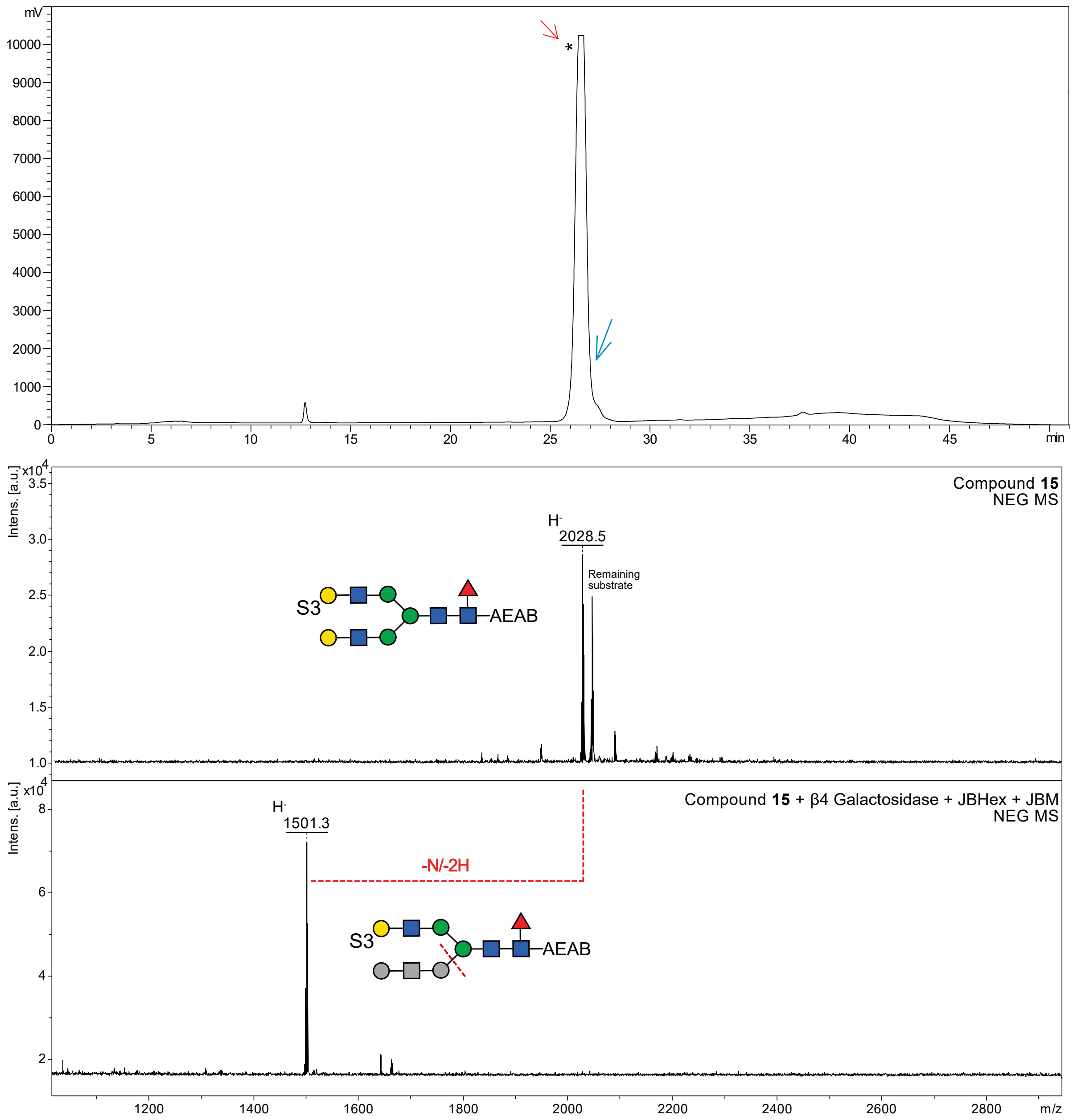

Compound 16: Biantennary core-fucosylated GalGal, modified with a 6-sulphate on the Gal residue (+CHST1 sulphotransferase) of the lower antenna. Position of the 6-sulphate was determined by digestion using a combination of β4-galactosidase, JBHex and JBM, which preferentially cleaves α-3 linkage of Man. The compound was resistant to JBM and the sulphate position on the lower antenna was also indicated by earlier elution time. Huang and colleagues report upper antennary preference of CHST1 (Huang *et al*), whereas in this study equal amounts of single-sulphated products were observed (**16** and **17**) but not sufficient double sulphated compound.

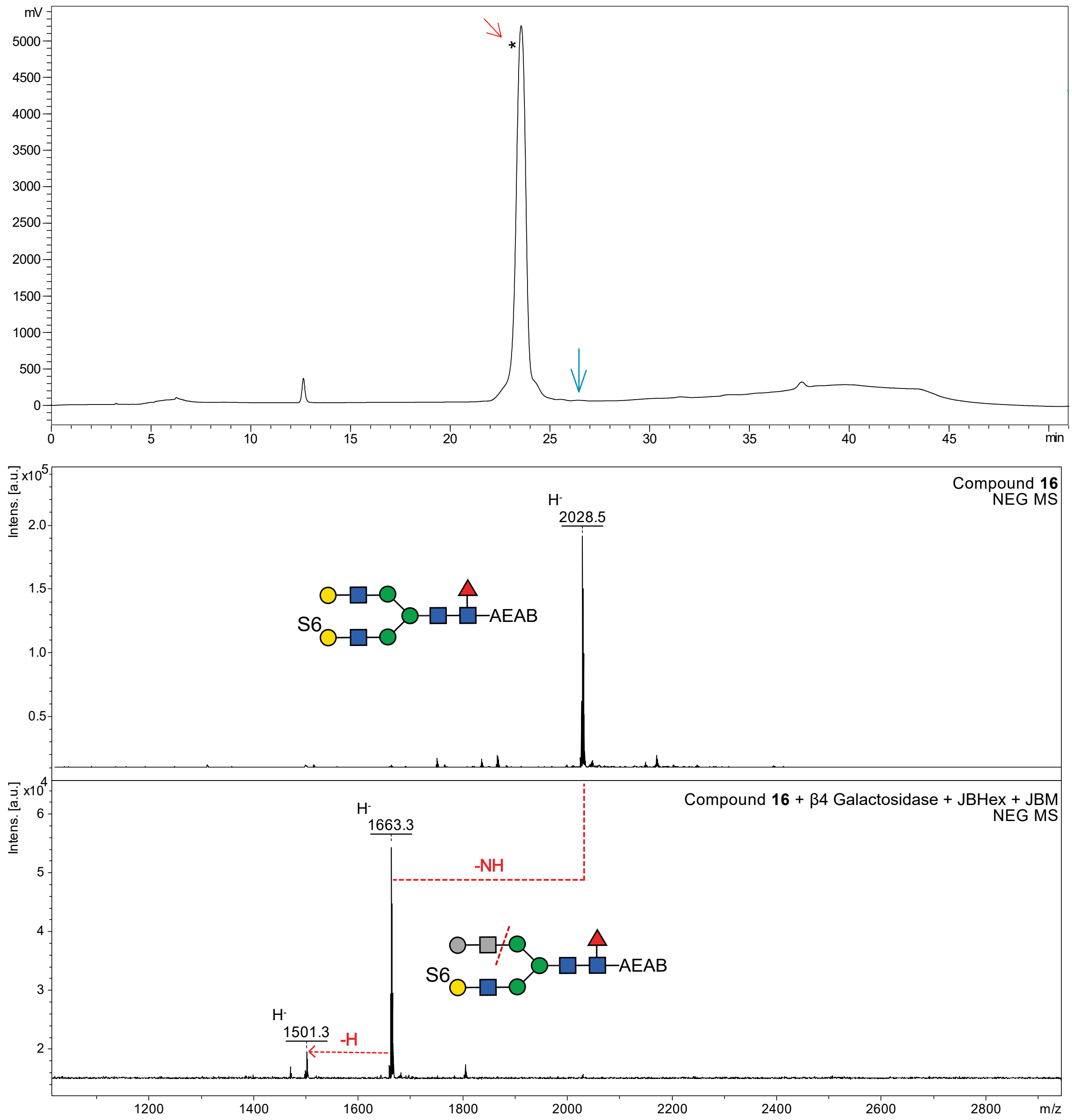

Compound 17: Biantennary core-fucosylated GalGal, modified with a 6-sulphate on the Gal residue (+CHST1) of the upper antenna. The compound was sensitive to all applied glycosidases.

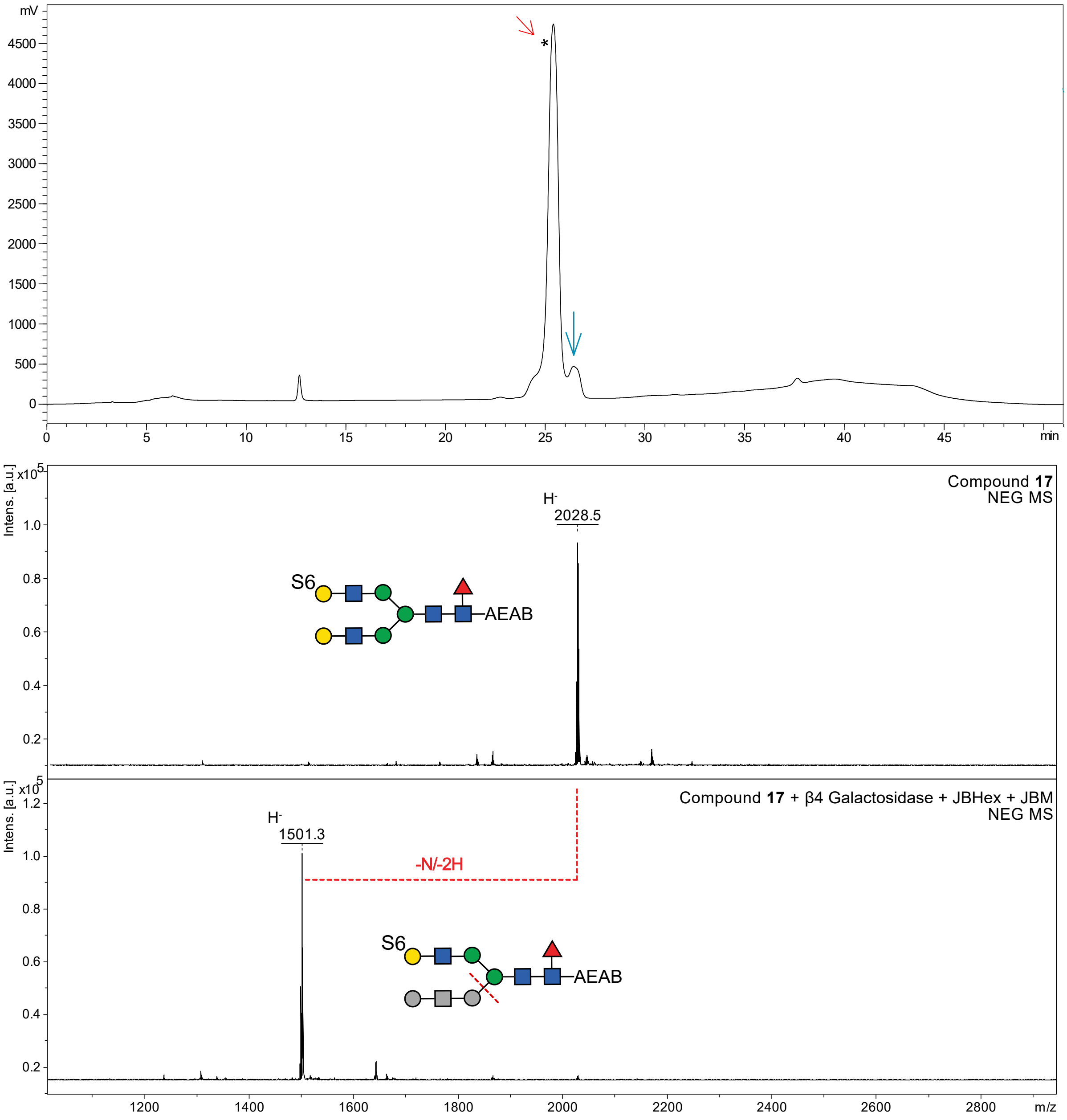

Compound 18: Biantennary core-fucosylated GnGn (commercial compound), purified by RP-HPLC.

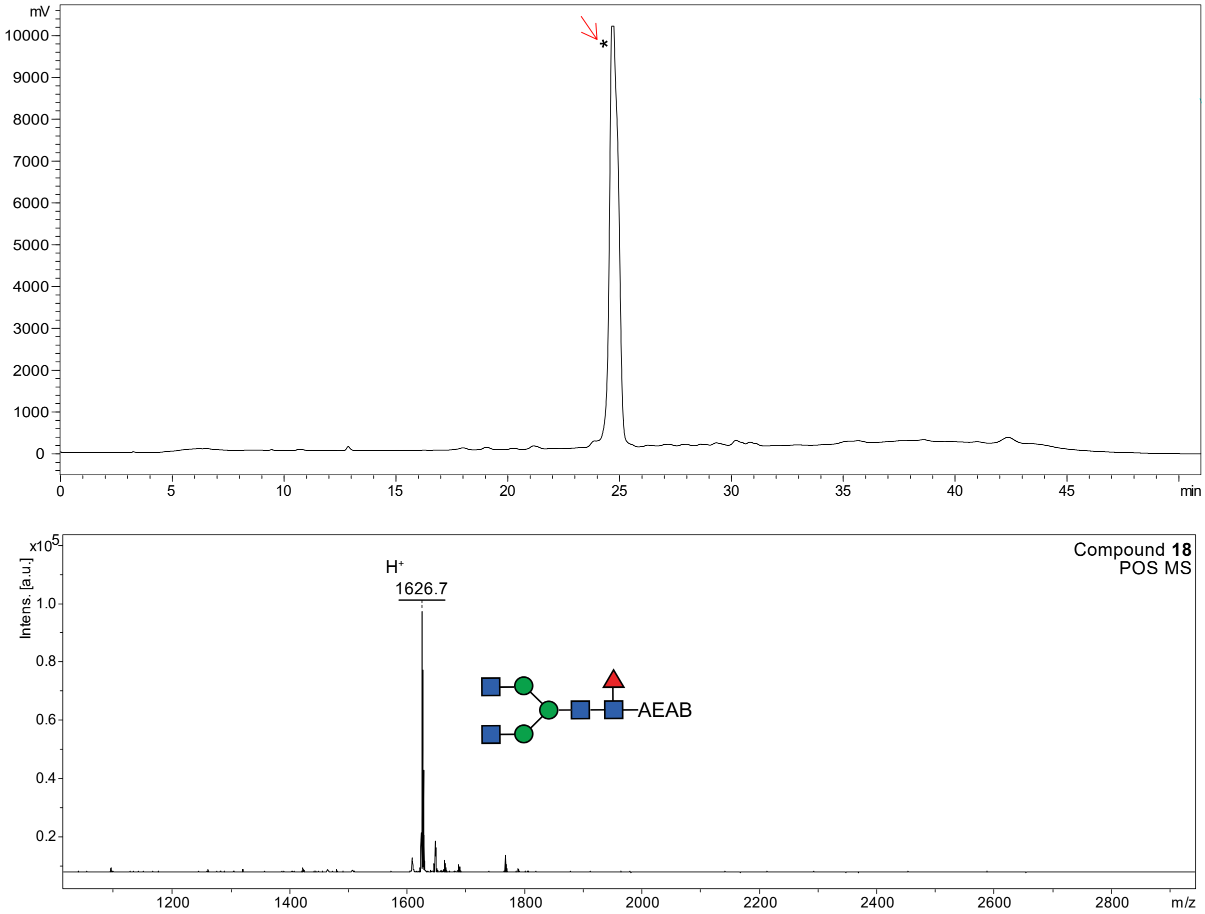

Compound 20: LNnT (lacto-*N*-neotetraose) modified with terminal GlcA (+B3GAT1). MS/MS spectrum is shown for demonstration and key fragments annotated. Nearly quantitative conversion was obtained.

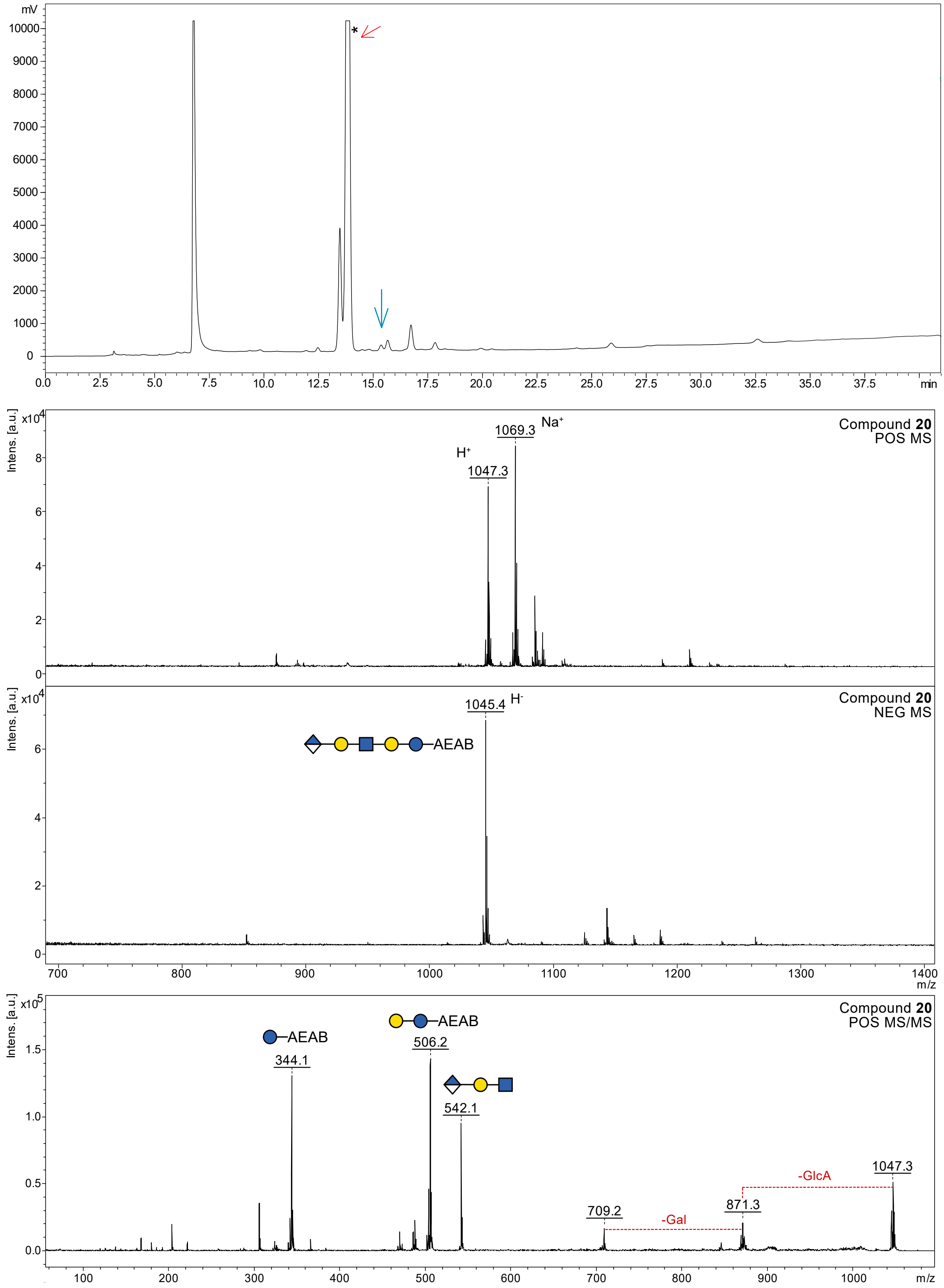

Compounds 21 and 22: Terminal β4-Gal was removed from LNnT. Remodelled with terminal β3-Gal (+B3GalT5 galactosyltransferase) and addition of GlcA (+B3GAT1), respectively. Both compounds were purified in a joint HPLC run.

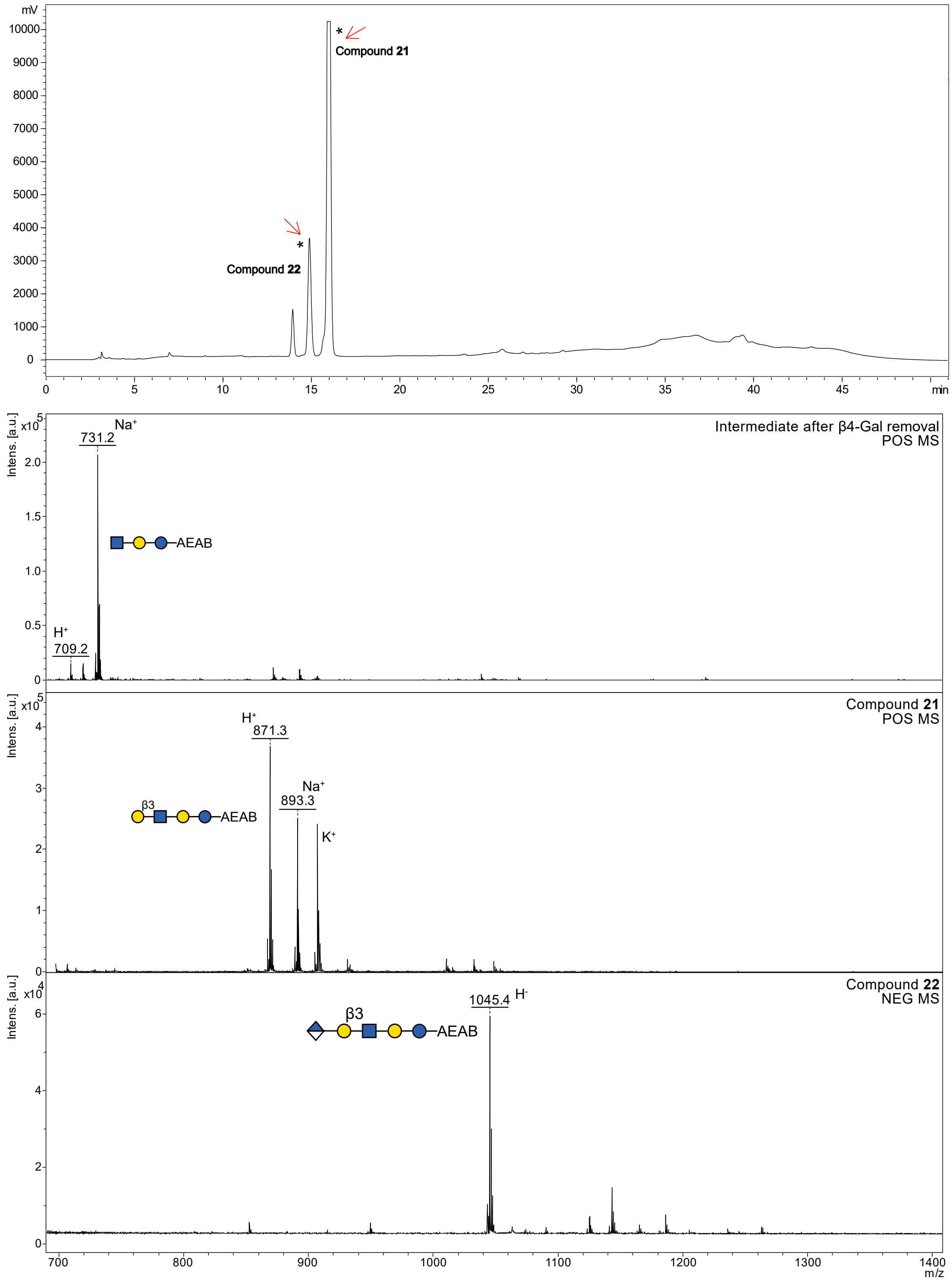

Compound 23: Terminal β4-Gal was removed from LNnT; modified with terminal GalNAc (+Gal-T1 (Y289L)). Nearly quantitative conversion was obtained.

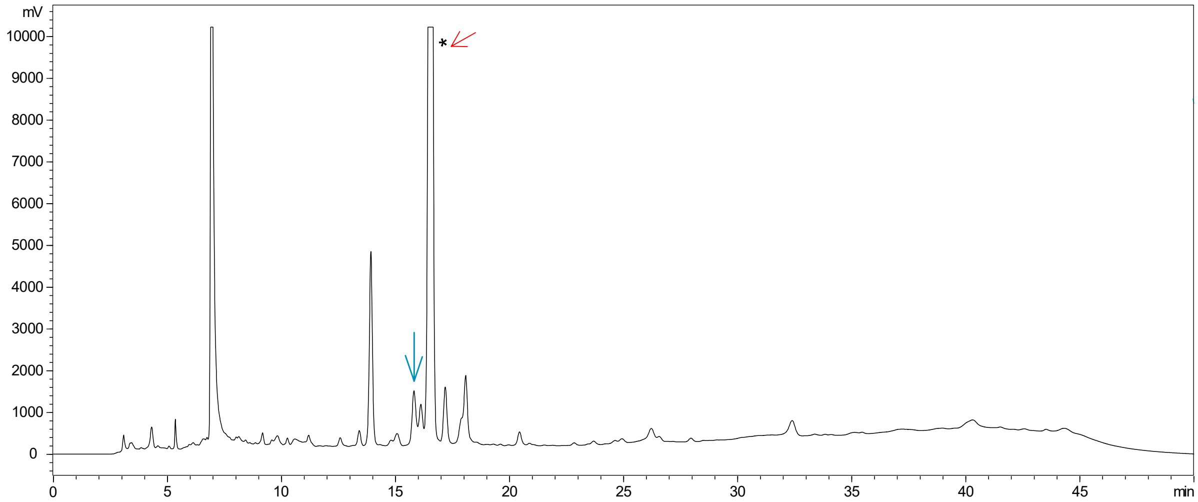

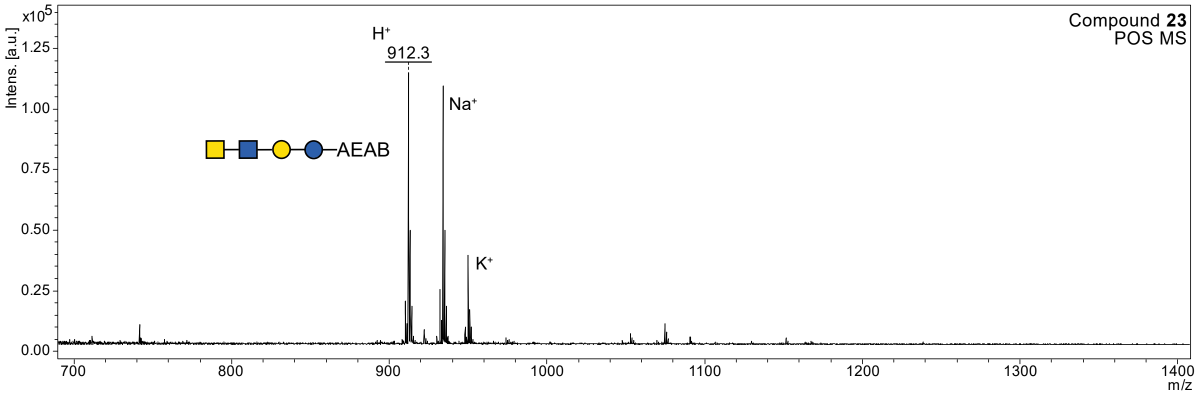

Compound 24: LNnT modified with terminal sulphated GlcA (+CHST10). Negative mode MS/MS spectrum is shown for demonstration and key fragments annotated. Nearly quantitative conversion was obtained.

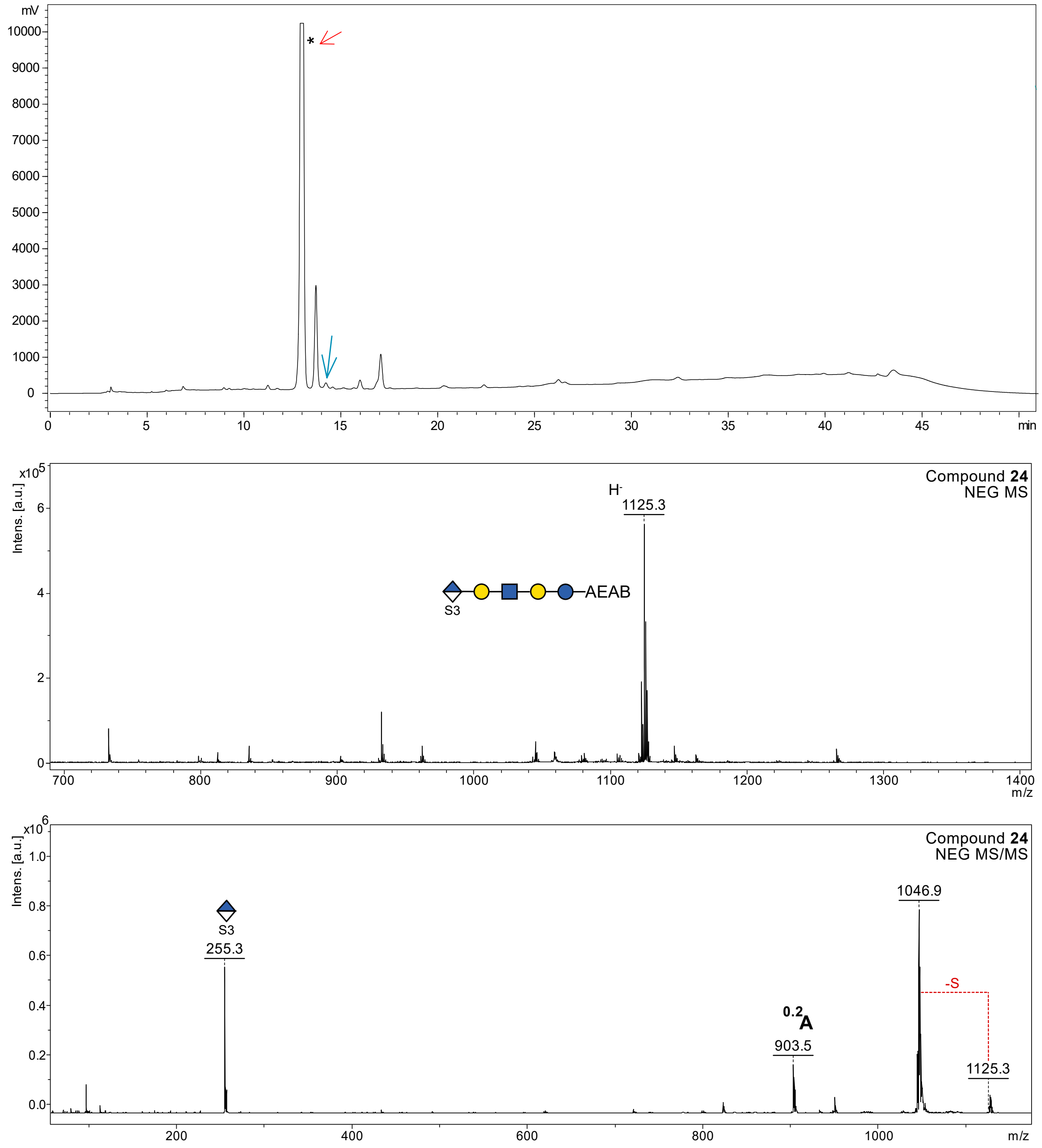

Compound 25: Terminal β4-Gal was removed from LNnT; modified with 6-sulphate on GlcNAc (+CHST2). Presence of sulphate was confirmed by resistance to enzymatic digest by JBHex (data not shown). To increase product amounts for printing (only approximately 10% conversion was achieved), the reaction was repeated twice, the compound purified and the respective fractions of all runs pooled.

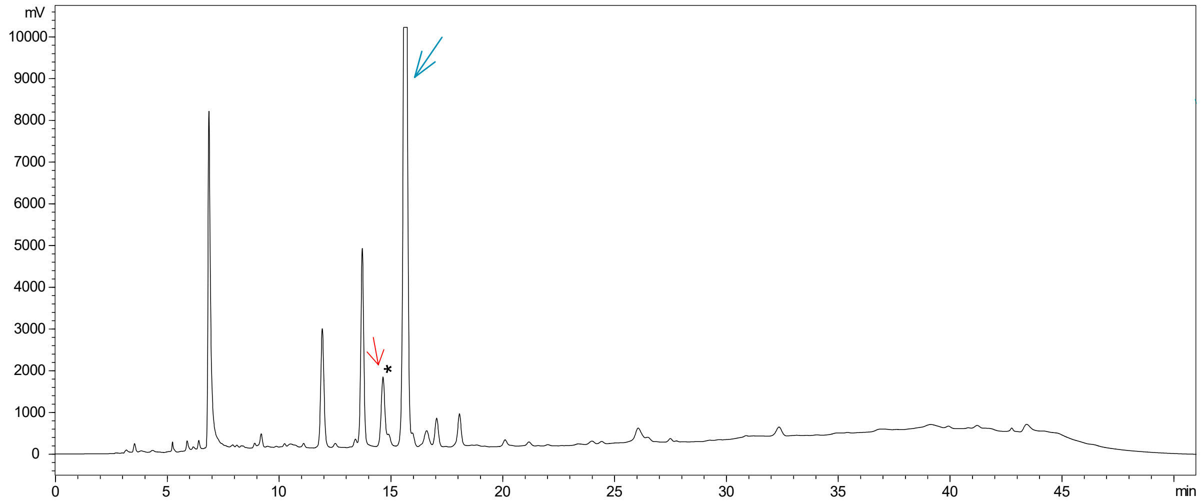

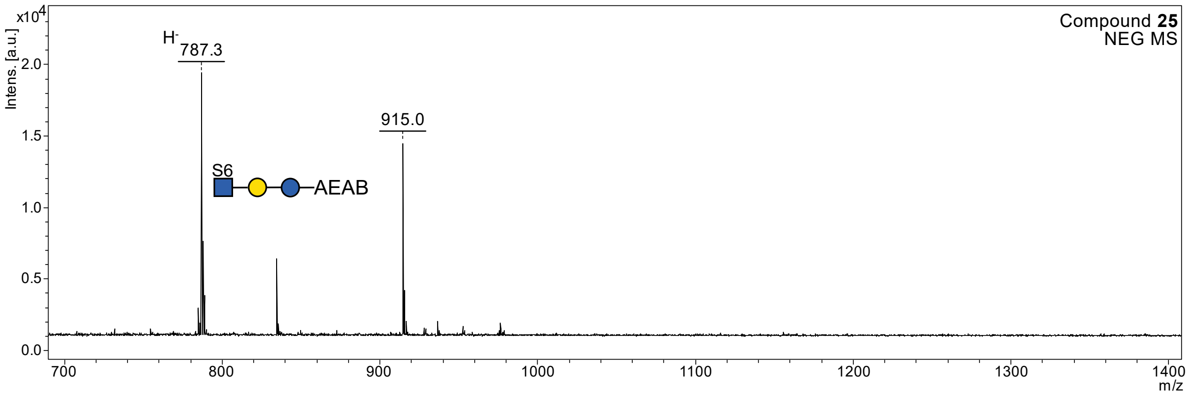

Compounds 26 and 27: LNnT modified with 6-sulphate on Gal (+CHST1). RP-HPLC revealed two peaks with *m/z* 949, therefore *Asp. nidulans* β4-galactosidase was applied to distinguish the positions of the sulphate on the galactose. Resistance to β4-galactosidase was observed for the terminal sulphate-modified Gal (**27**).

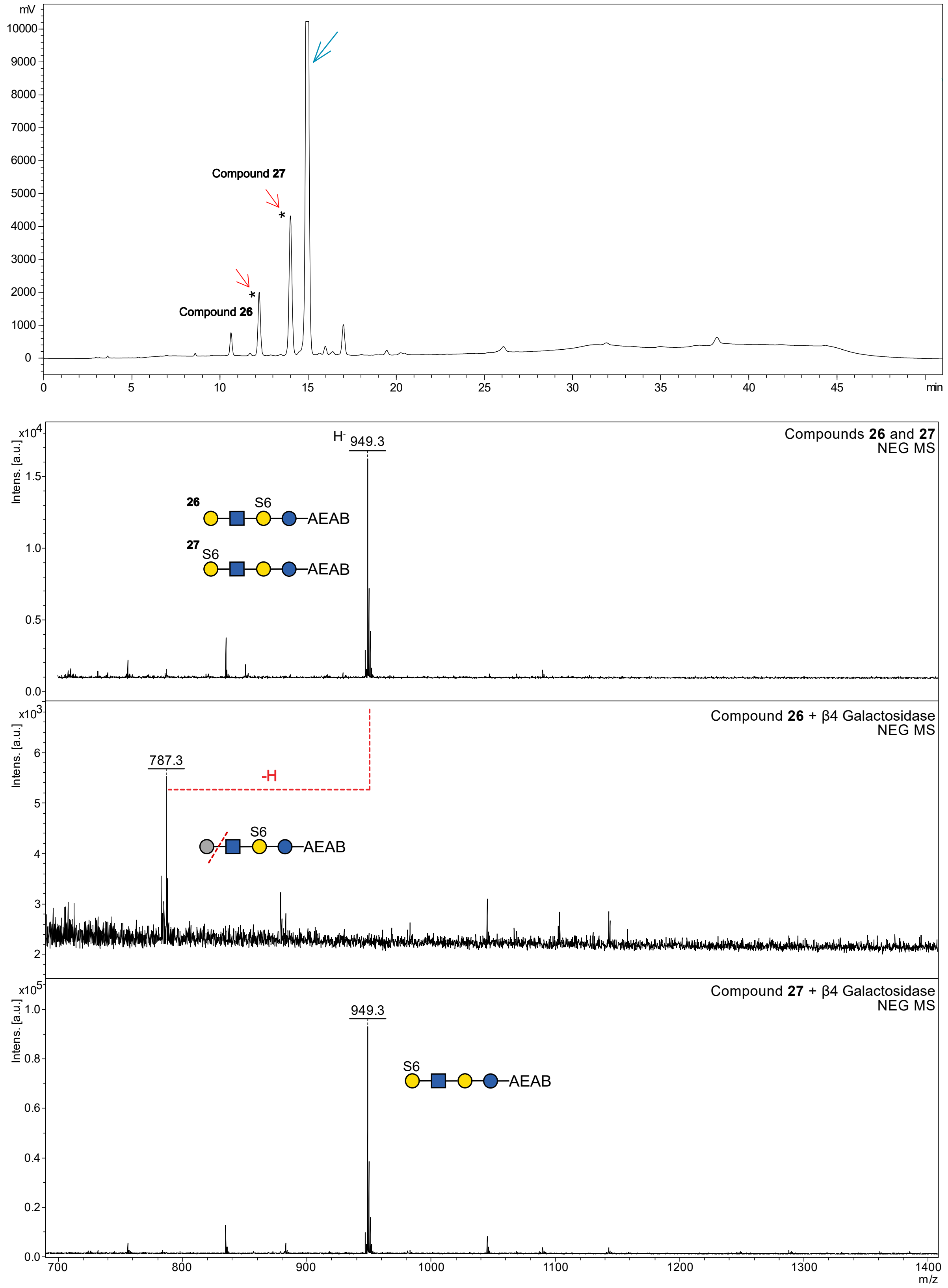

Compounds 26 and 27 (continued): Negative mode MS/MS spectra are shown and key fragments annotated to confirm the different sulphate position.

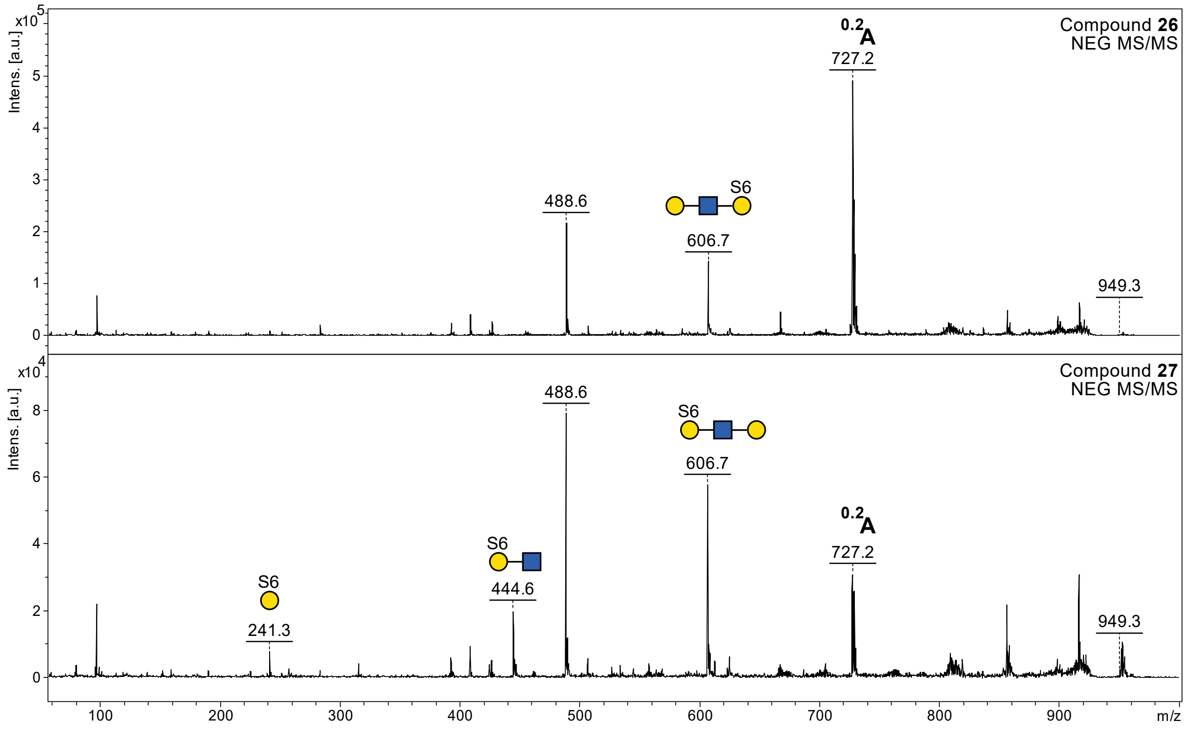

Compound 28: LNnT modified with 3-sulphate on Gal (+GAL3ST2). Position of the sulphate on terminal Gal was verified by *Asp. nidulans* β4-galactosidase resistance (data not shown).

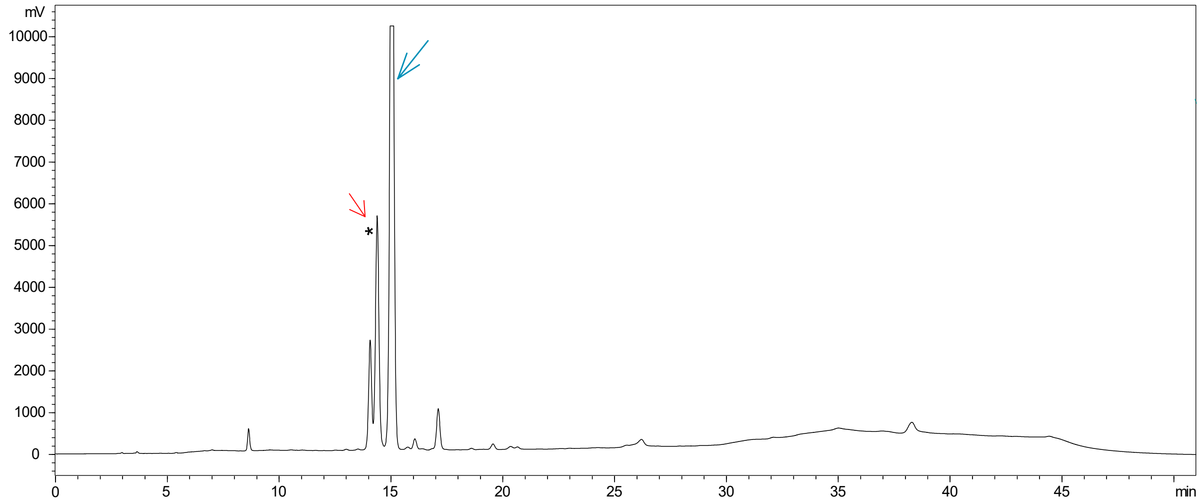

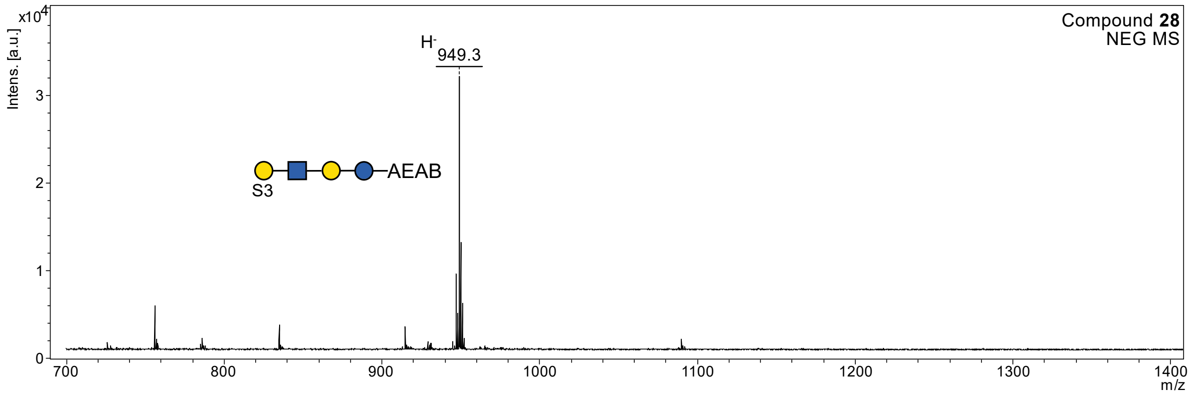

Compound 29: LNnT modified with terminal α-2,3 Neu5Ac (+NmST3).

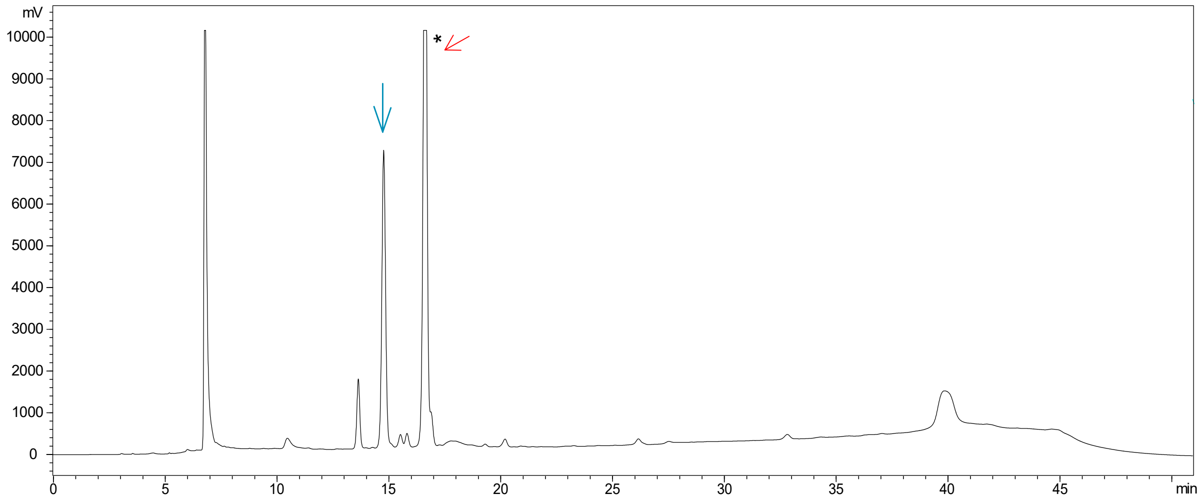

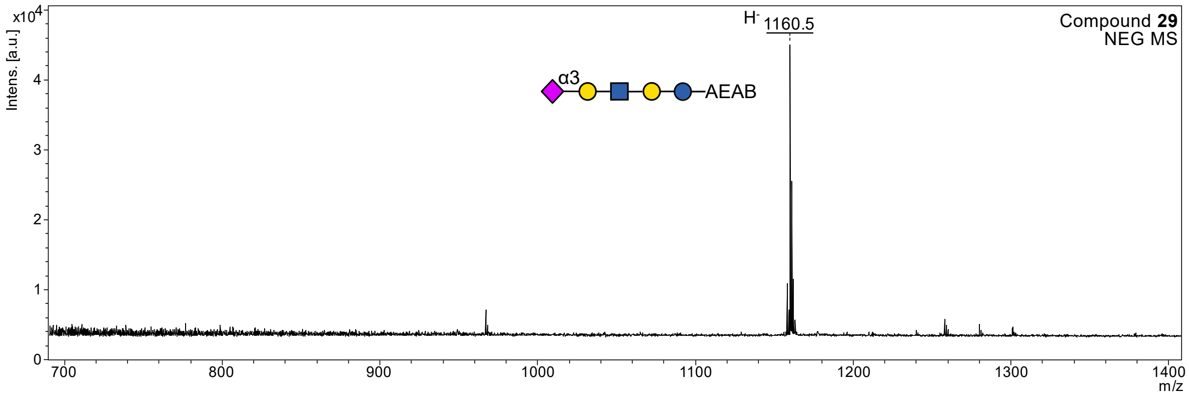

Compound 30: LNnT modified with terminal α-2,6 Neu5Ac (+PdST6).

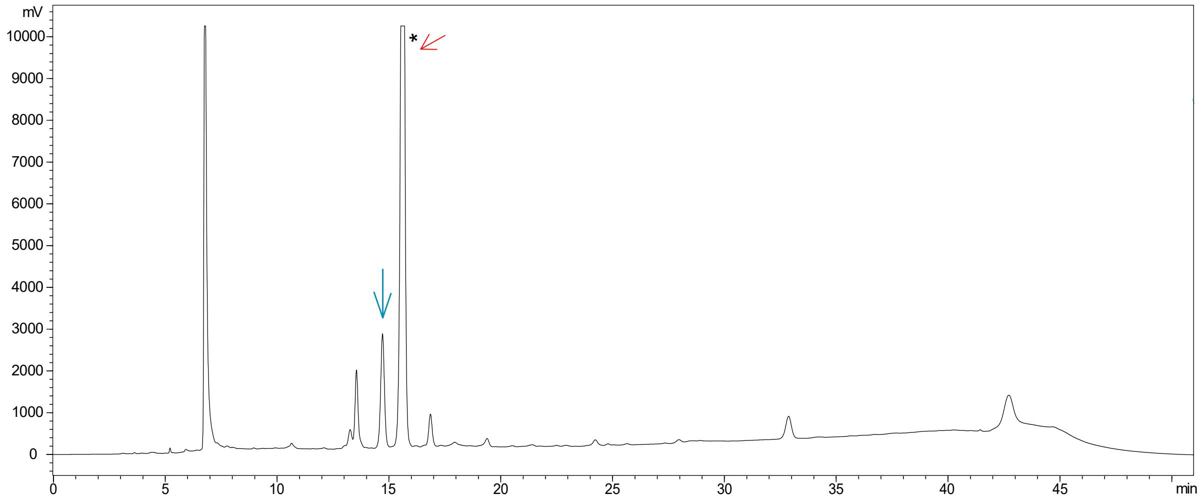

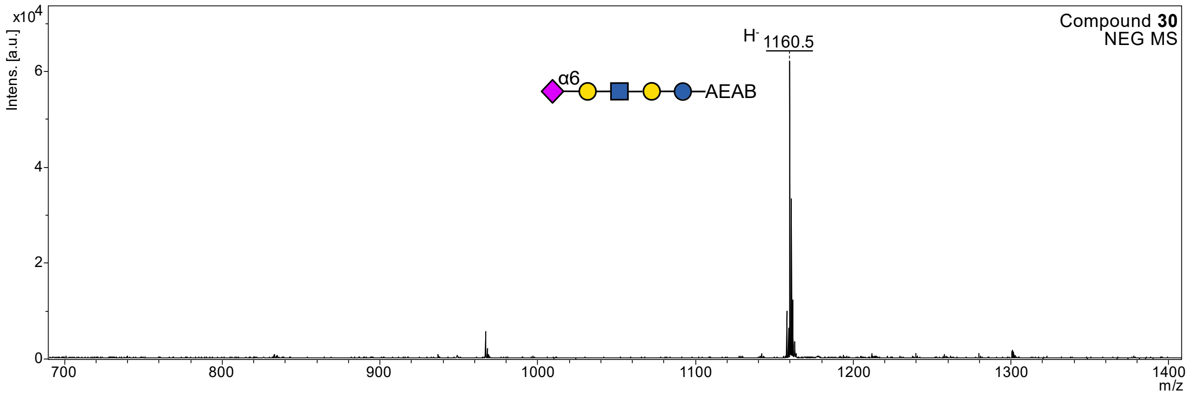

Compound 31: LNnT modified with terminal α-2,3 Neu5Gc (+ST3GAL4). Due to co-elution with unmodified substrate, the fraction from the first run was digested with *Asp. nidulans* β4-galactosidase and JBHex and re-applied to the HPLC, so the sialylated product could be separated from the trimmed substrate.

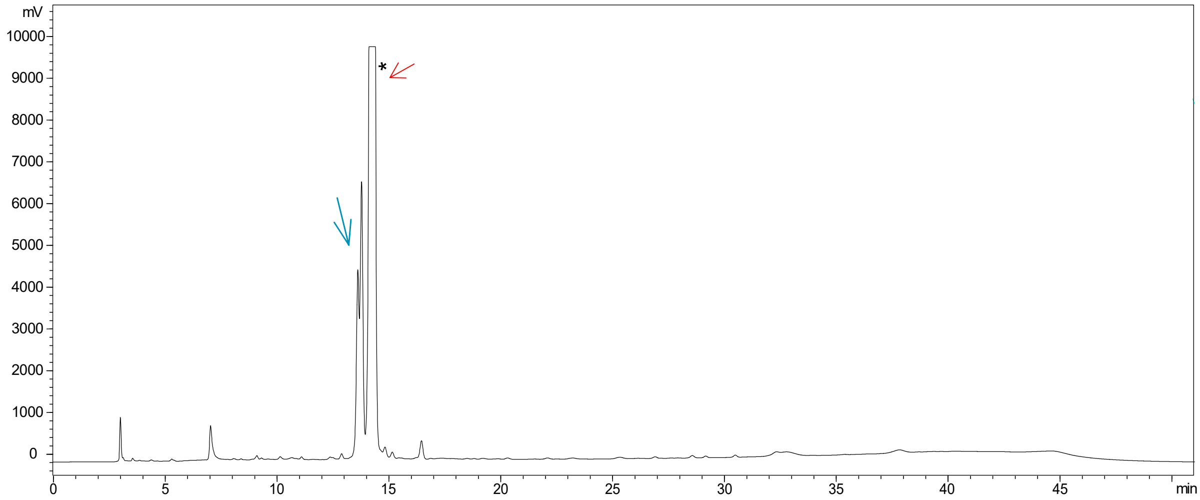

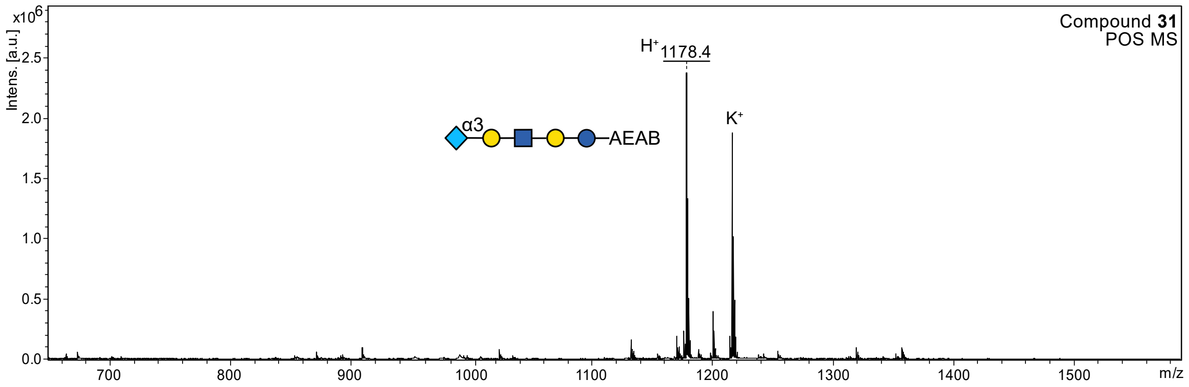

Compound 32: LNnT modified with terminal α-2,6 Neu5Gc (+PdST6).

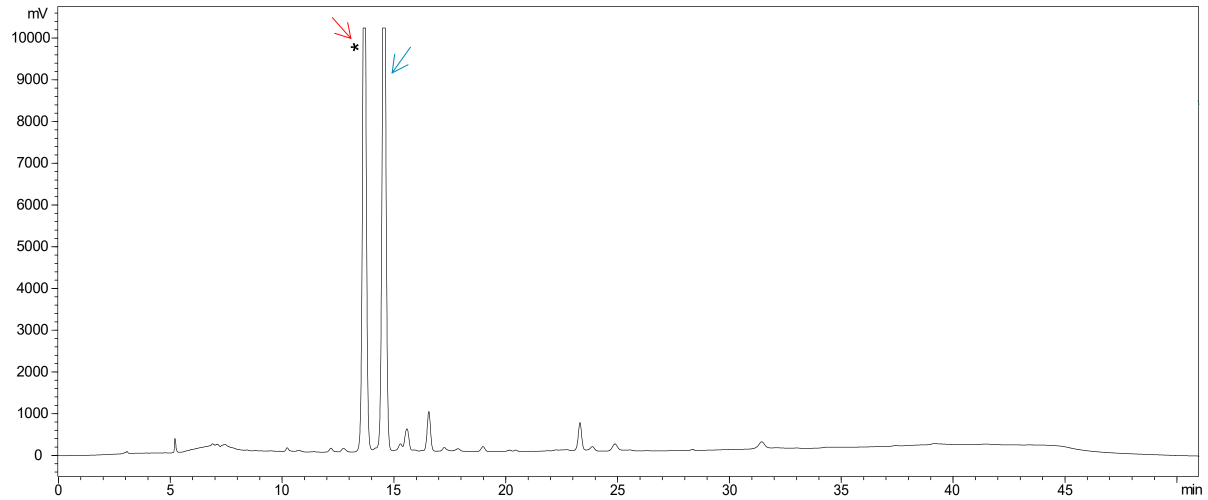

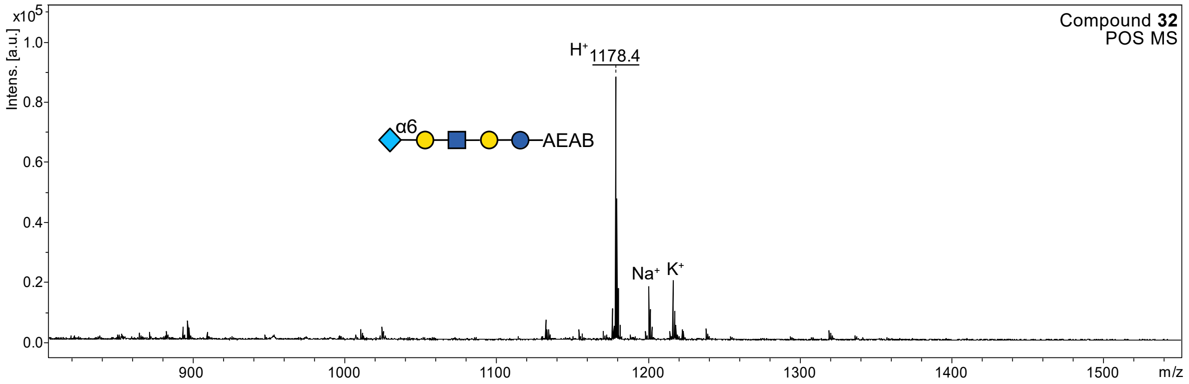

Compound 33: LNnT modified with terminal α-2,3 Neu5Ac (+ST3GAL4) and α3 fucosylated GlcNAc (sLe^X epitope, +FUT3 fucosyltransferase). Interestingly, FUT3 could fucosylate not only GlcNAc but also Glc, therefore fucose position was necessary to be verified by MS/MS.

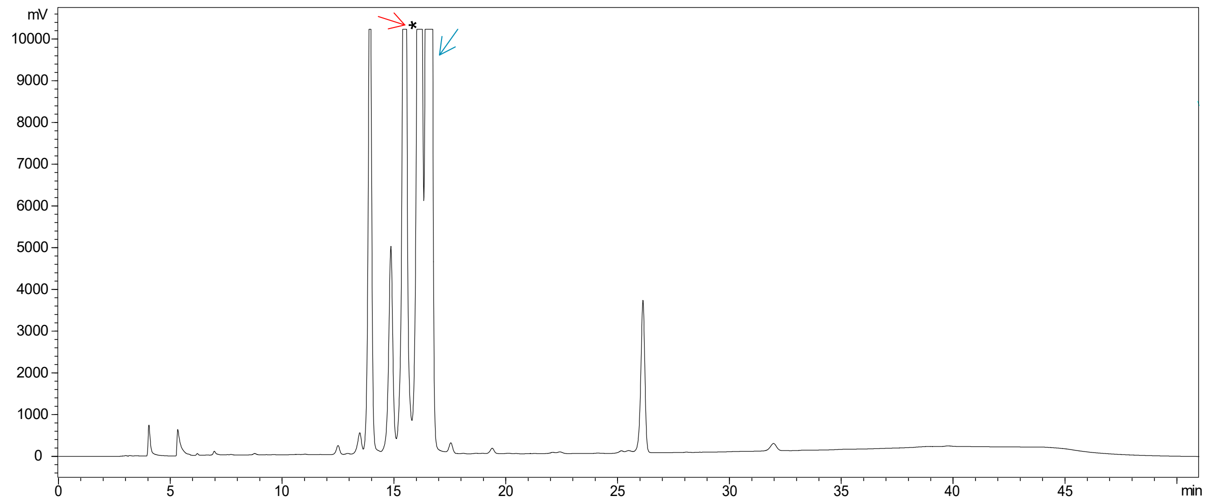

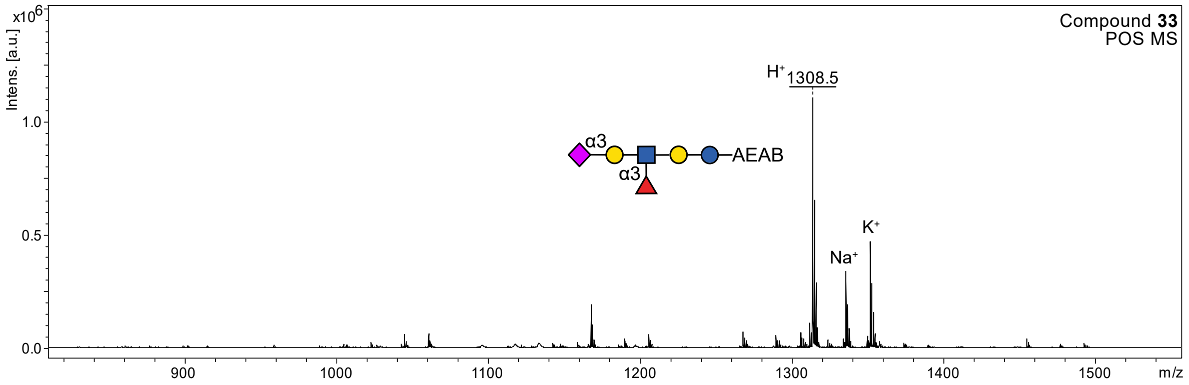

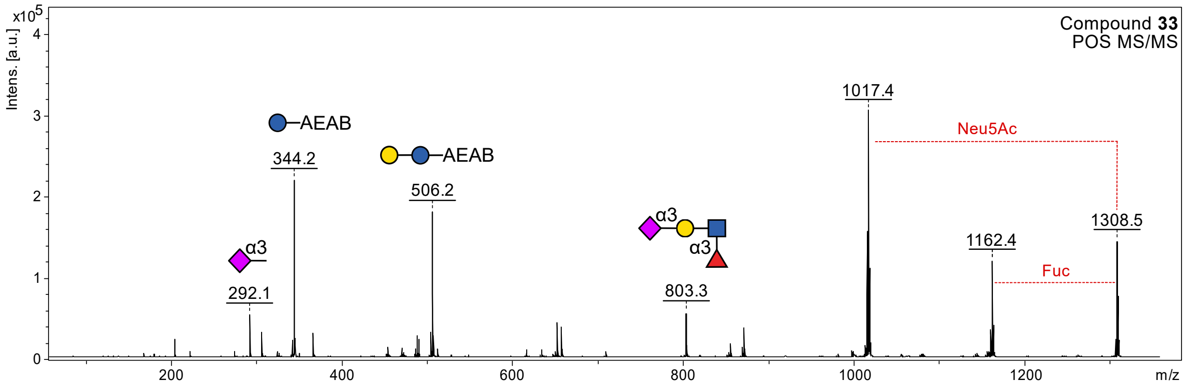

Compounds 34 and 37: LNnT modified with terminal α-2,3 Neu5Gc (+ST3GAL4) and α3 fucosylated GlcNAc (sLe^{X}-like epitope with Neu5Gc, +FUT3). Combined sialylated and unmodified fraction was used in the FUT3 assay and fucose position was verified by MS/MS. Compound **37** was only used later as positive control for the anti-LewisX antibody.

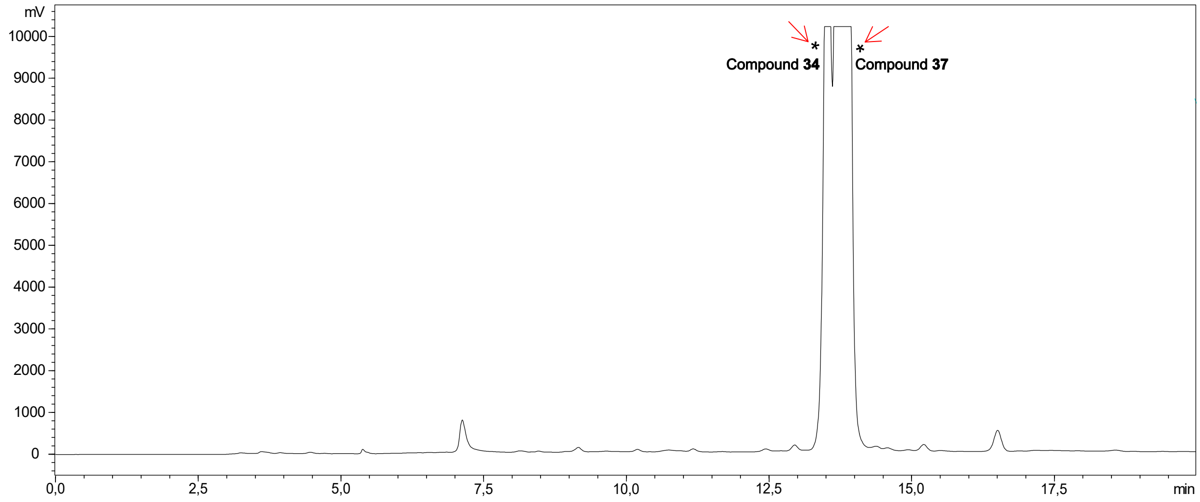

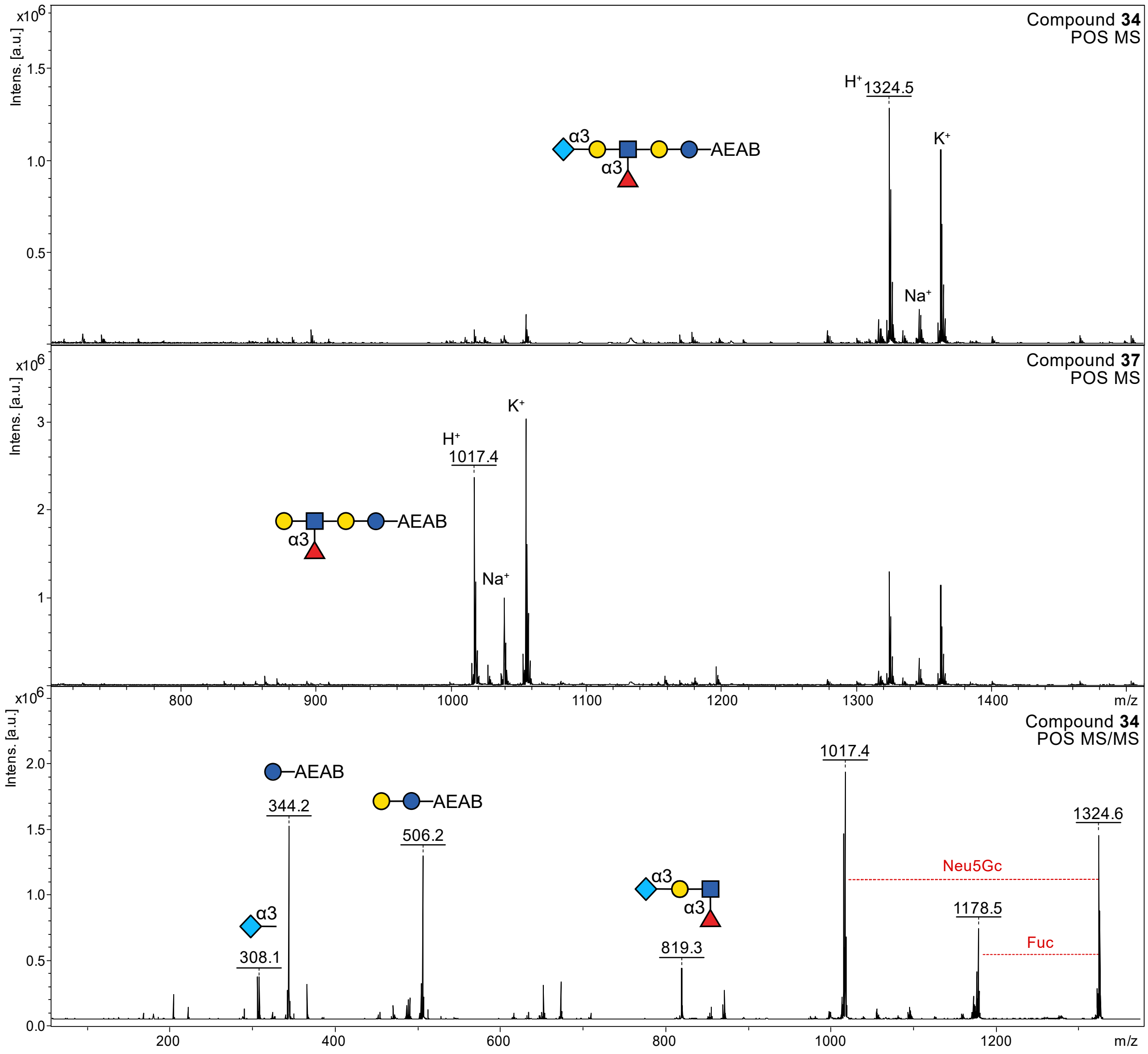

Compound 35: LNT modified with terminal α-2,3 Neu5Ac (+ST3GAL4) and α4 fucosylated GlcNAc (sLe^{A} epitope, +FUT3). Interestingly, FUT3 could fucosylate not only GlcNAc but also Glc, therefore fucose position was necessary to be verified by MS/MS.

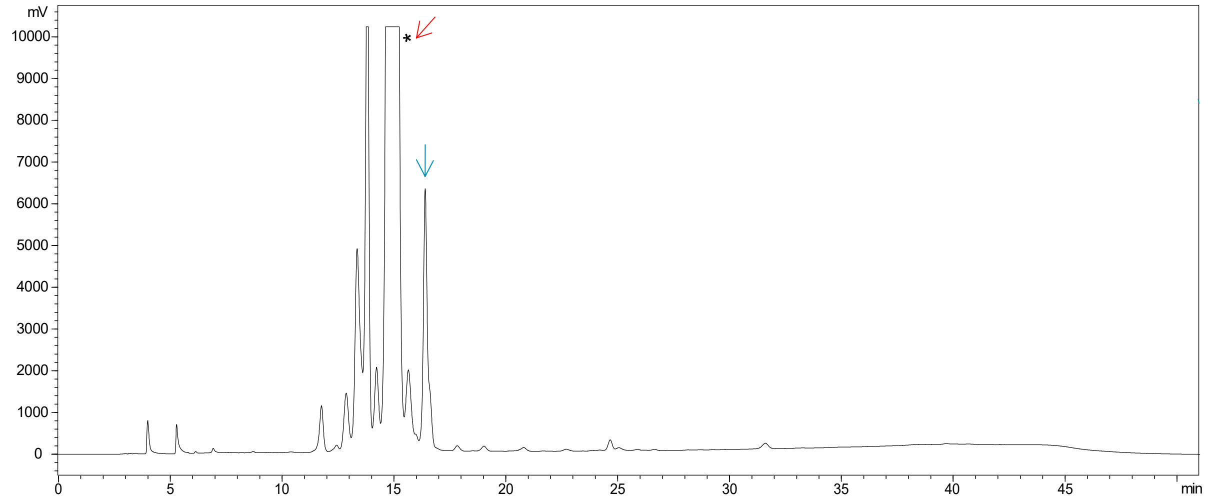

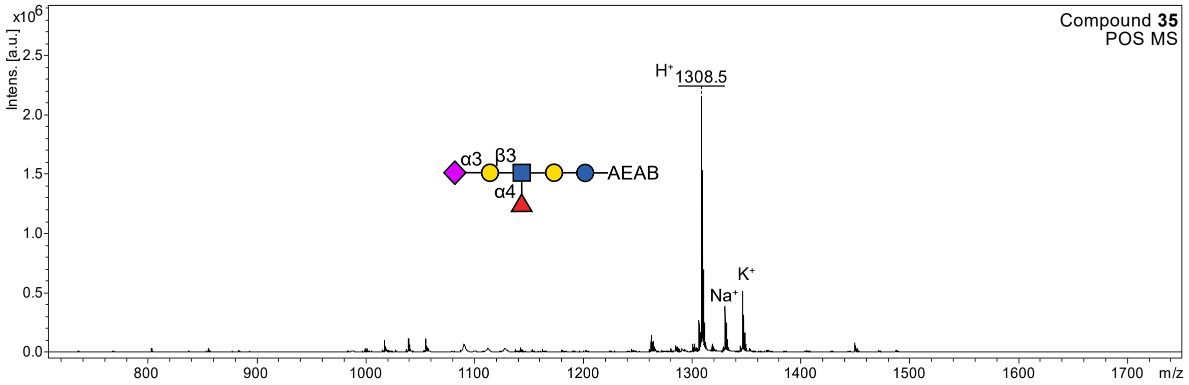

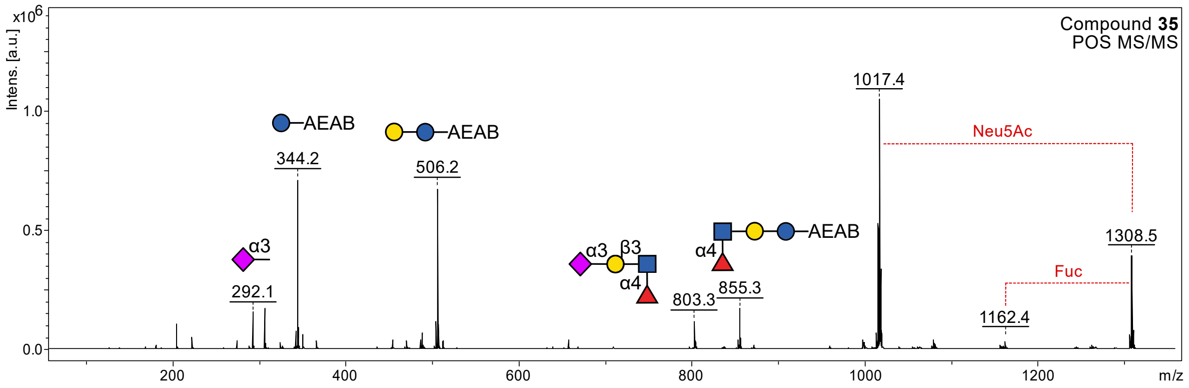

Compound 36: LNT modified with terminal α-2,3 Neu5Gc (+ST3GAL4) and α4 fucosylated GlcNAc (sLe^{A}-like epitope with Neu5Gc, +FUT3). Fucose position was verified by MS/MS.

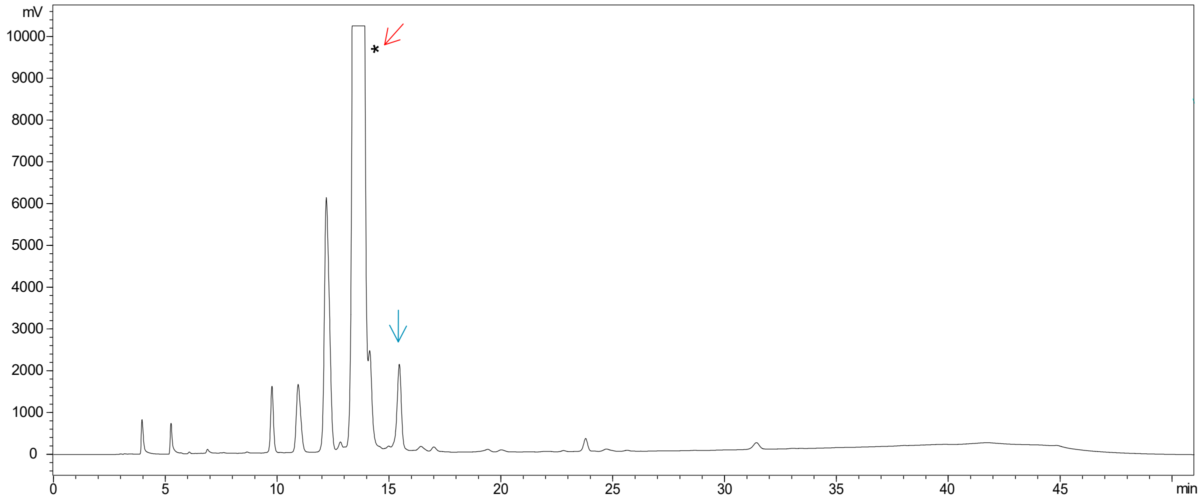

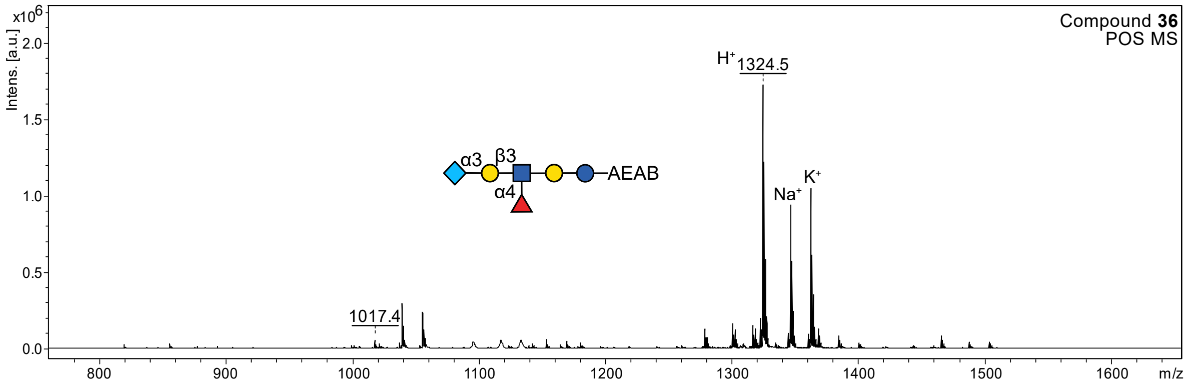

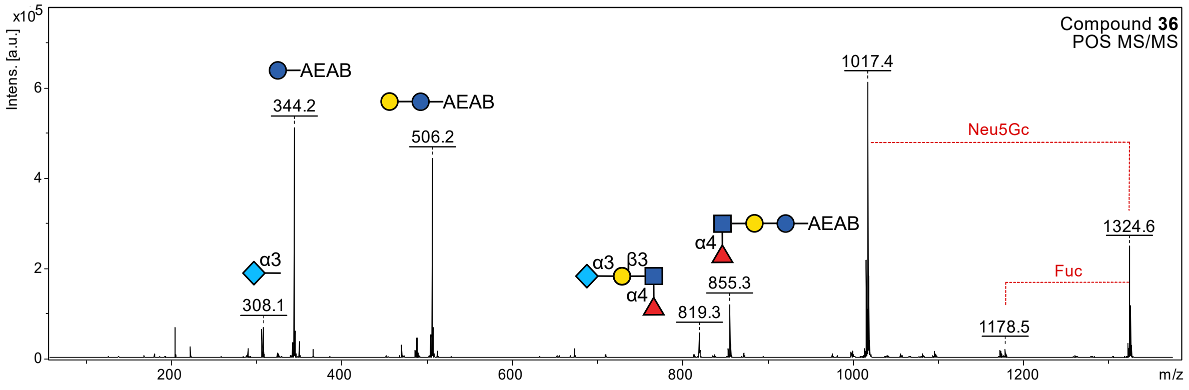

Compound 38: LNT modified with α4 fucosylated GlcNAc (Le^{A} epitope, +FUT3). Fucose position was verified by MS/MS.

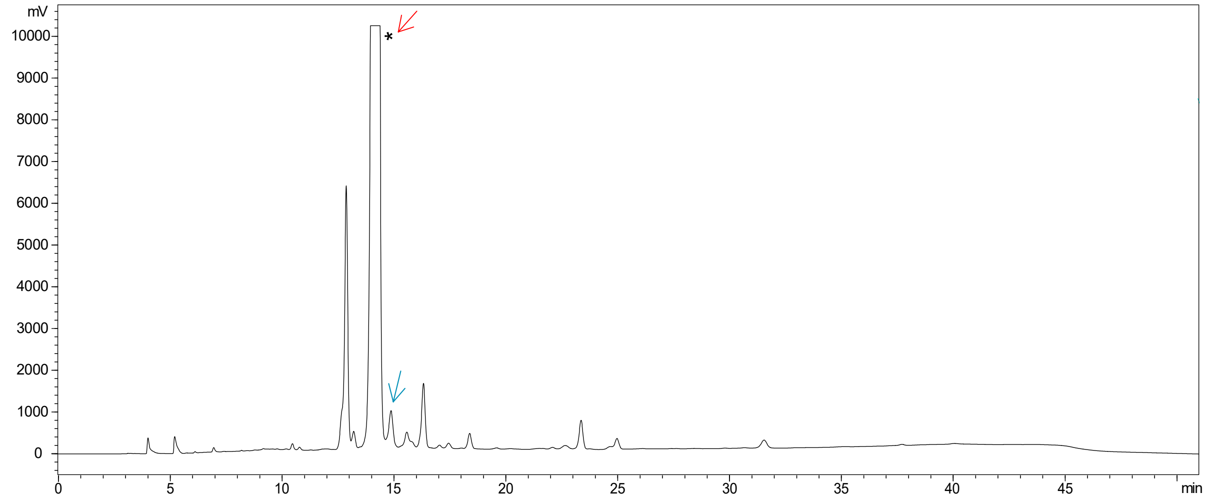

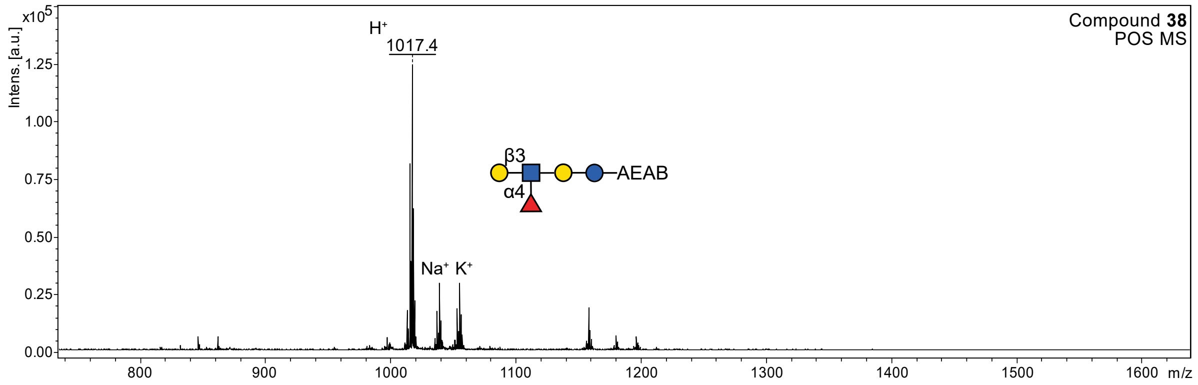

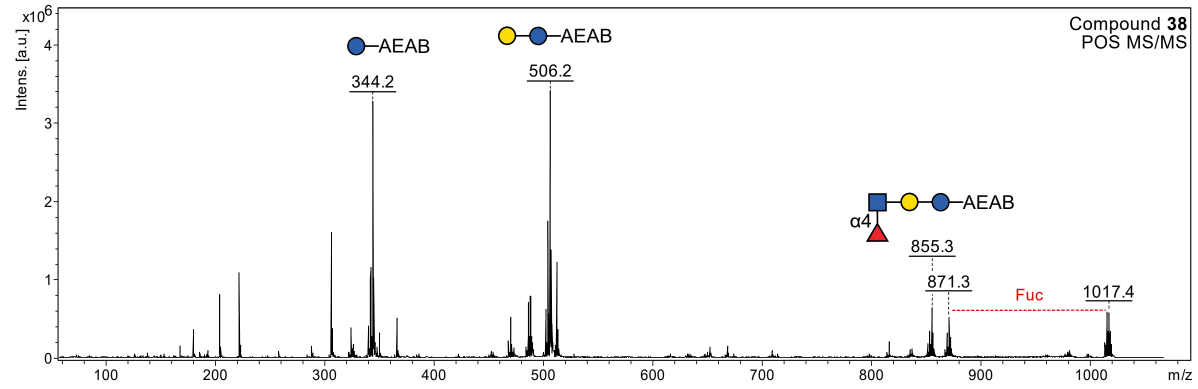

Compound 39: LNT modified with terminal α-2,3 Neu5Ac (+ST3Gal4).

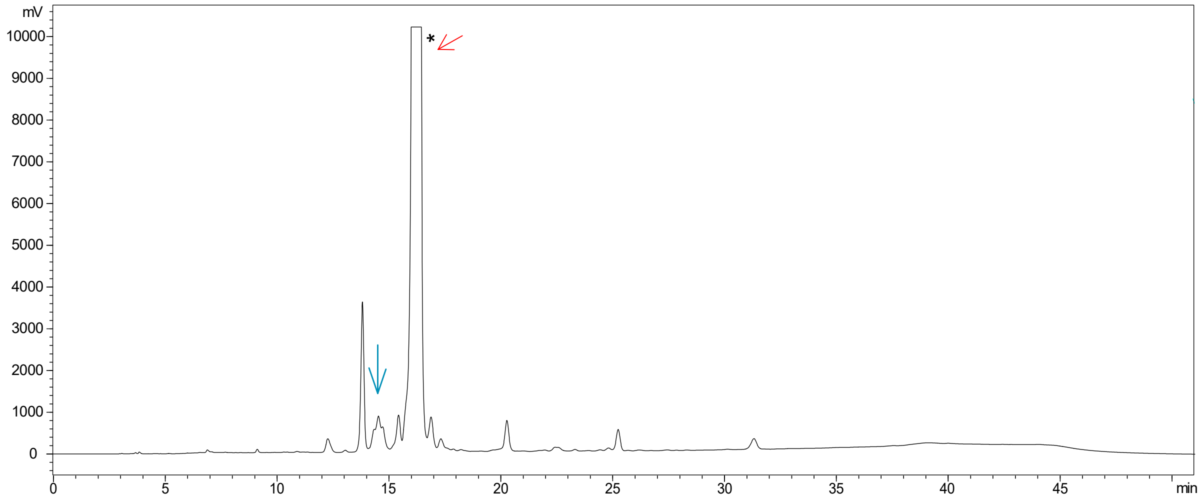

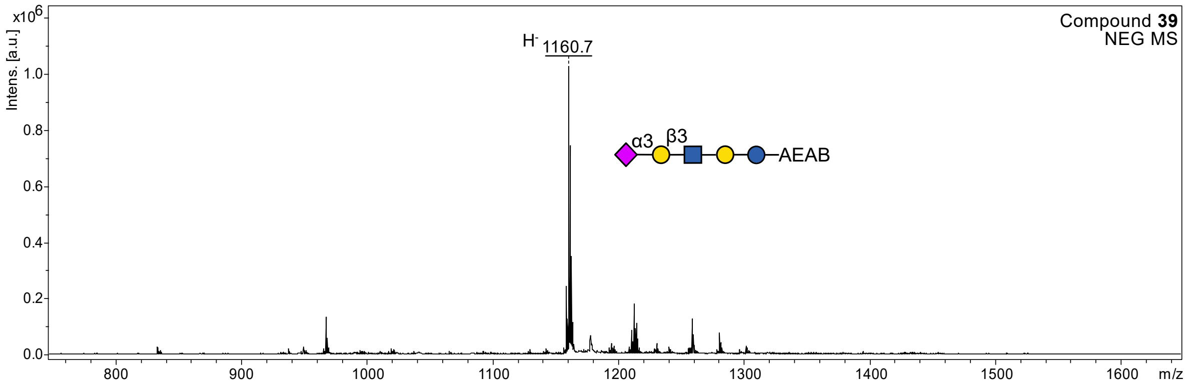

Compound 40: LNT modified with terminal α-2,3 Neu5Gc (+ST3Gal4).

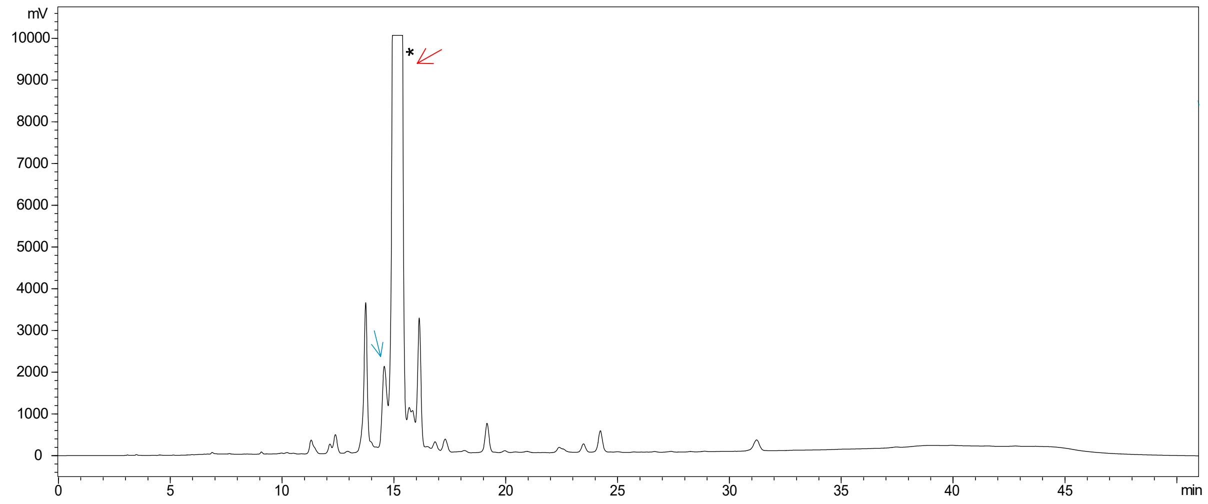

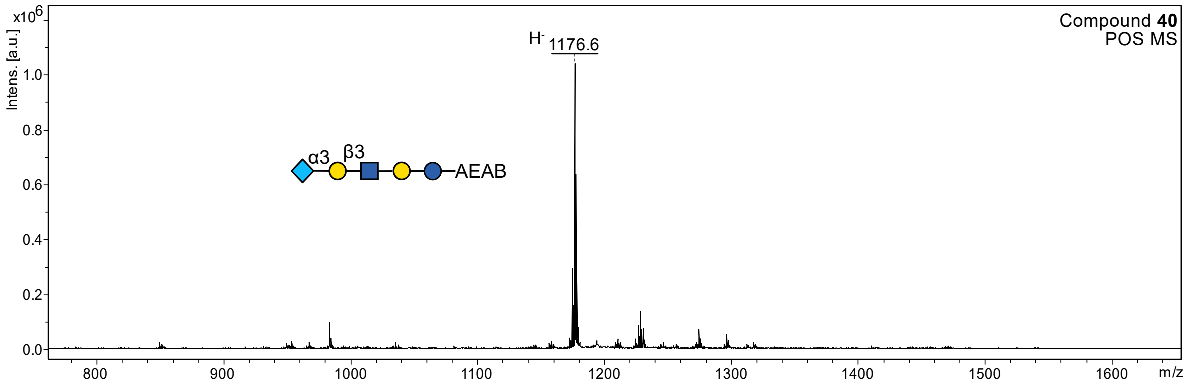

Compounds 41 and 42: LNnT modified with terminal α-2,3 Neu5Ac (+ST3GAL4), 6-sulphated galactose (+CHST1) and α3 fucosylated GlcNAc (+FUT3). Fucose position was determined by MS/MS (precursor ion *m/z* 1308). Spectra indicate a mixture of either fucosylated GlcNAc or Glc. The *m/z* Δ46 (similar to Δ55) was occasionally observed for sialic acids as well and is considered a sample variable artefact. Furthermore, the presence of the non-sialylated compounds (*m/z* 1095 or 949) is rather a result of in-source sialic acid loss as there is no indication of the respective non-sulphated ions (*m/z* 1017 or 871) in positive mode MS spectra.

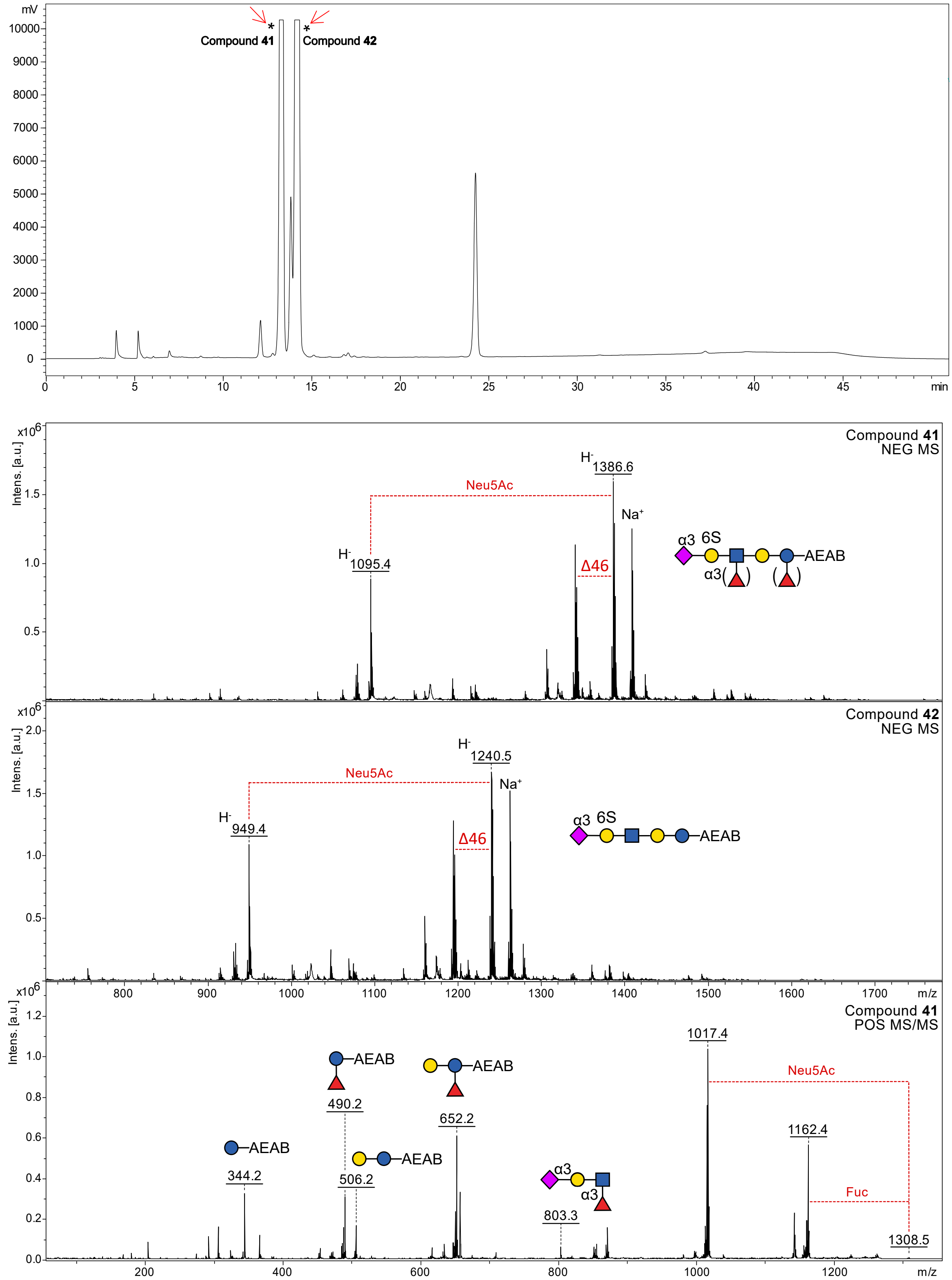

Compounds 43 and 44: LNnT modified with α2,3 Neu5Ac (+ST3GAL4) and one or two additional α2,8 Neu5Ac residues (+CstII sialyltransferase).

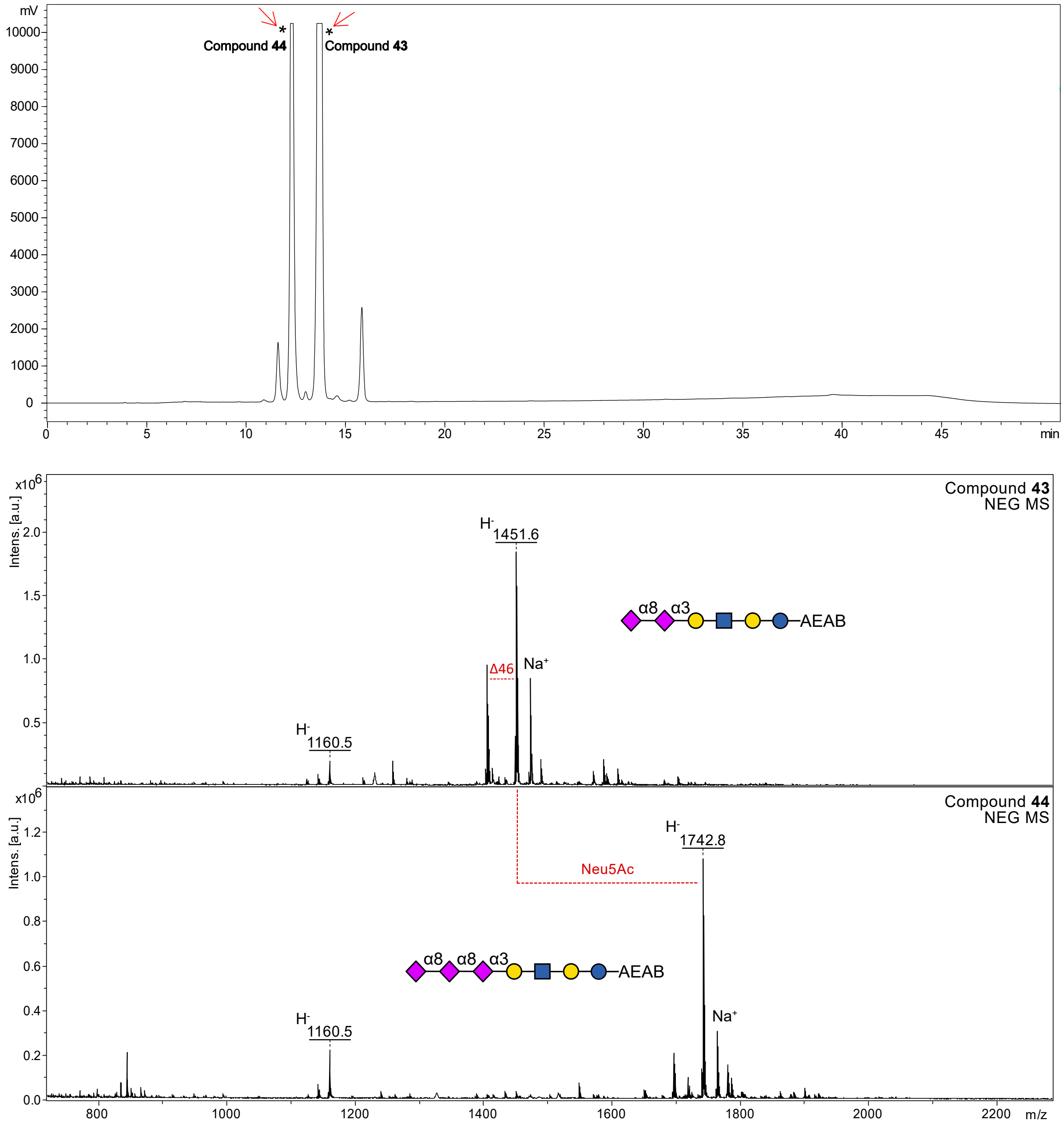

Compounds 45 and 46: AsialoGM1 (Elicityl) modified with α2,3 Neu5Ac (+ST3GAL4), resembling ganglioside GA1 and GM1b, respectively.

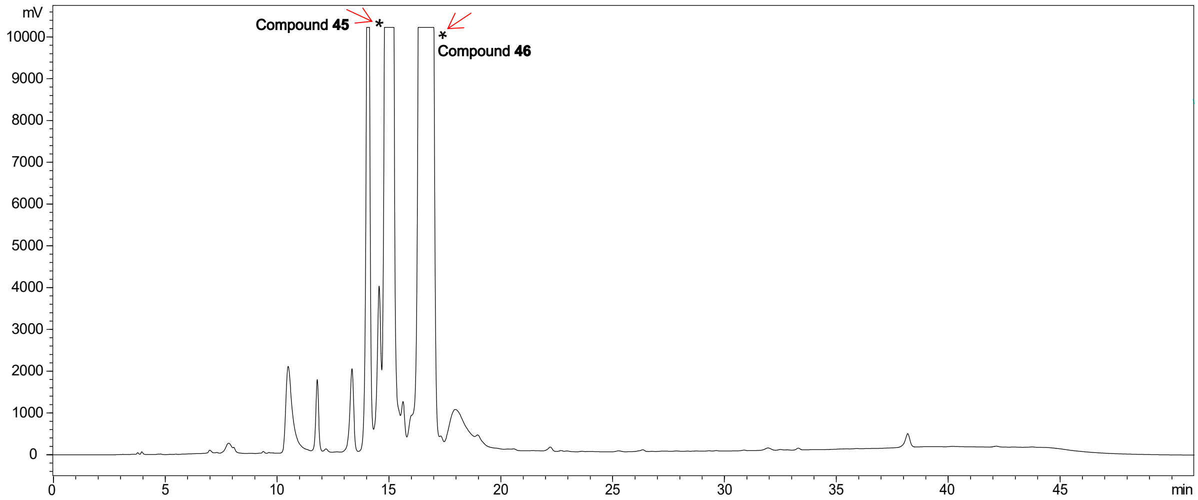

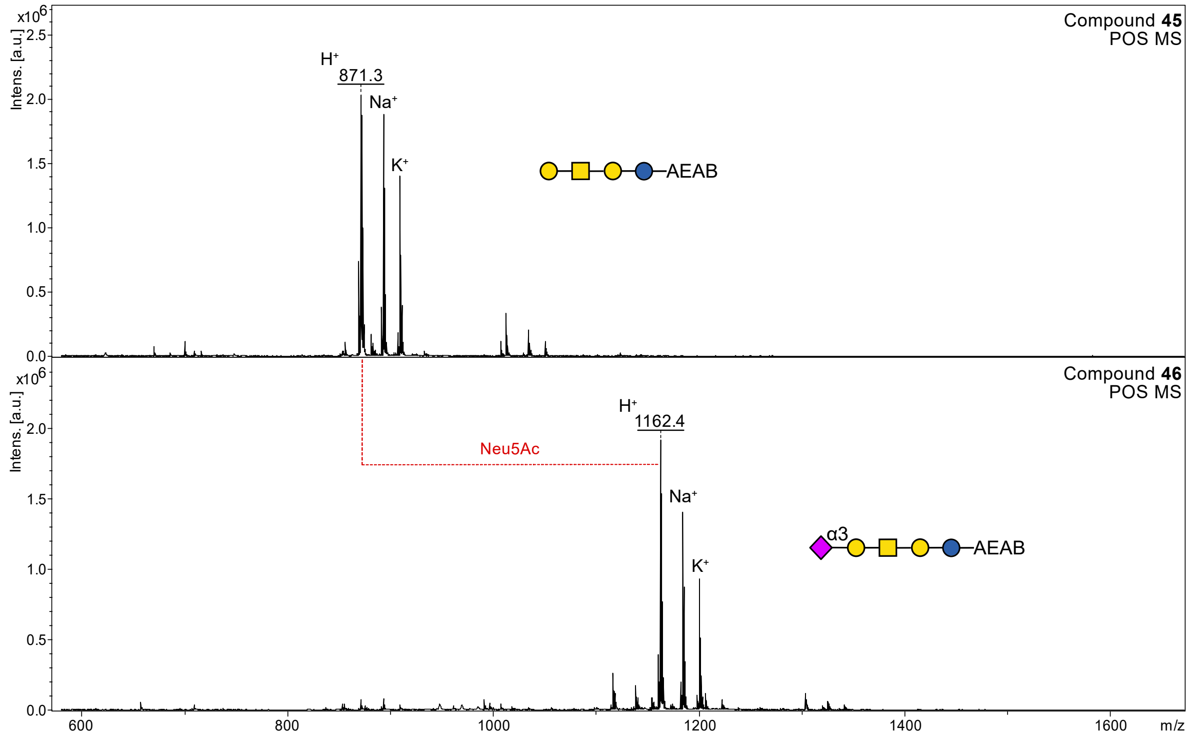

Compound 47: Compound 46 (ganglioside GM1b-type glycan) further modified with an additional α2,8 Neu5Ac residue (+CstII), resembling ganglioside GD1c.

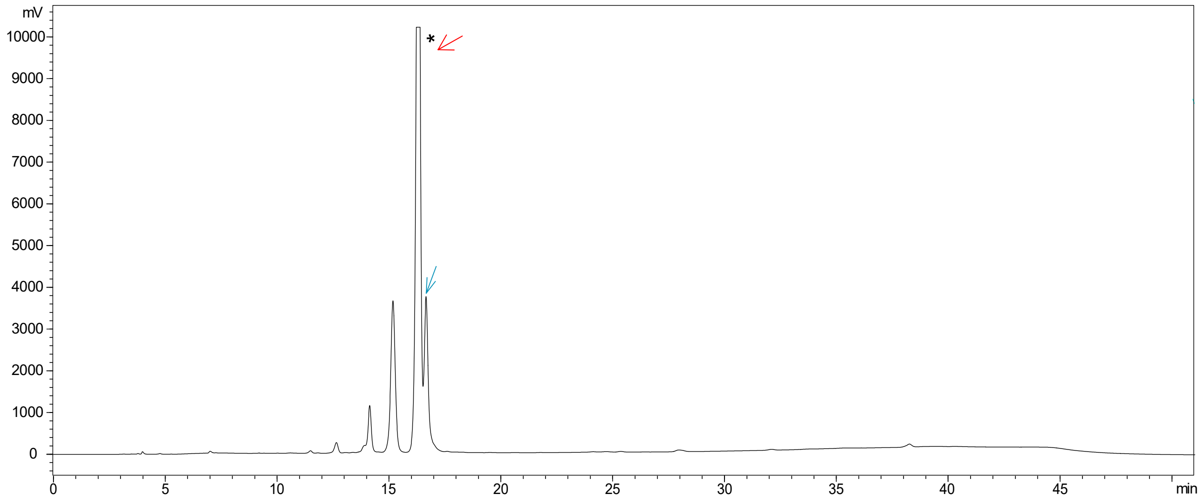

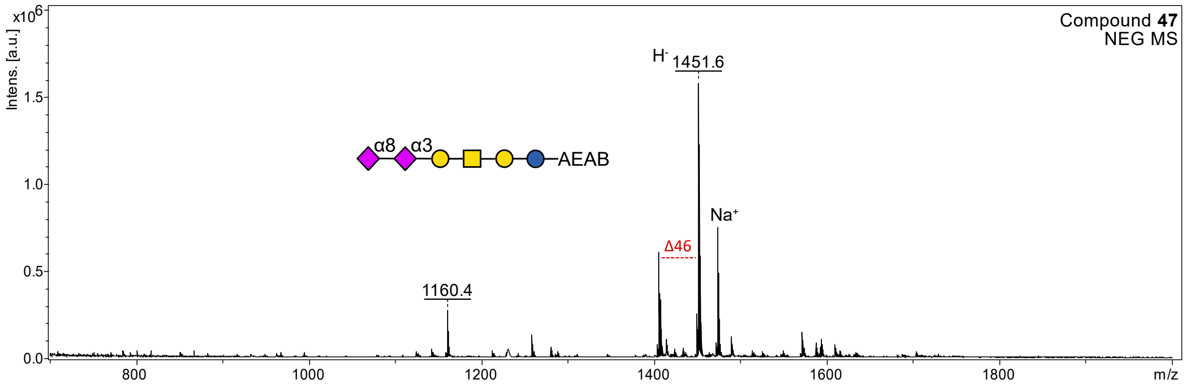

Compounds 48 and 49: Terminal β4-Gal and GlcNAc were removed from LNnT (+Asp. nidulans β4-galactosidase + JBHex), obtaining a substrate resembling the glycosphingolipid-type glycan lactosylceramide (LacCer). Remodelled with terminal α2,3 Neu5Ac (+PmST3), it resembles the simple ganglioside GM3. Both compounds were purified in a joint HPLC run.

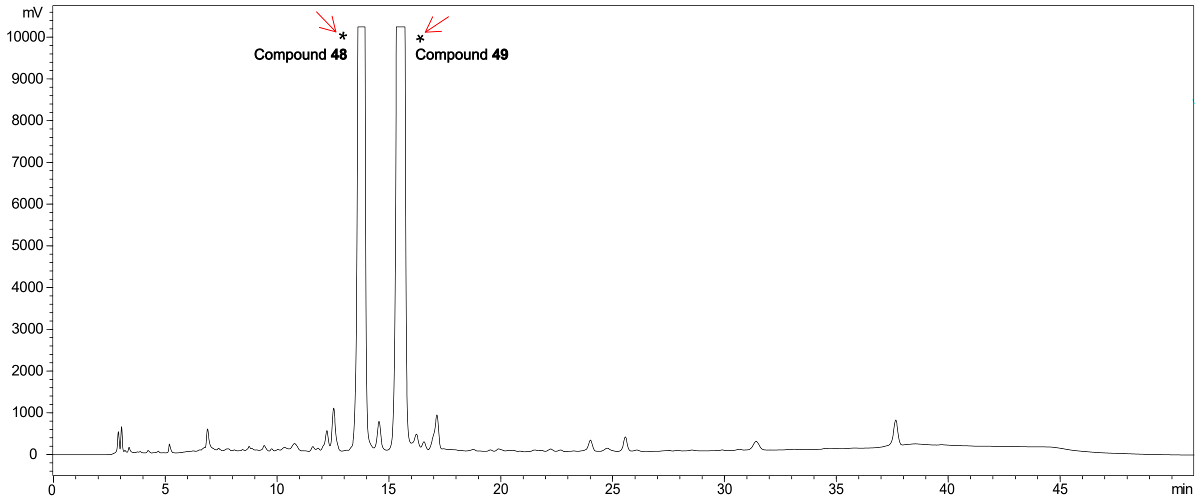

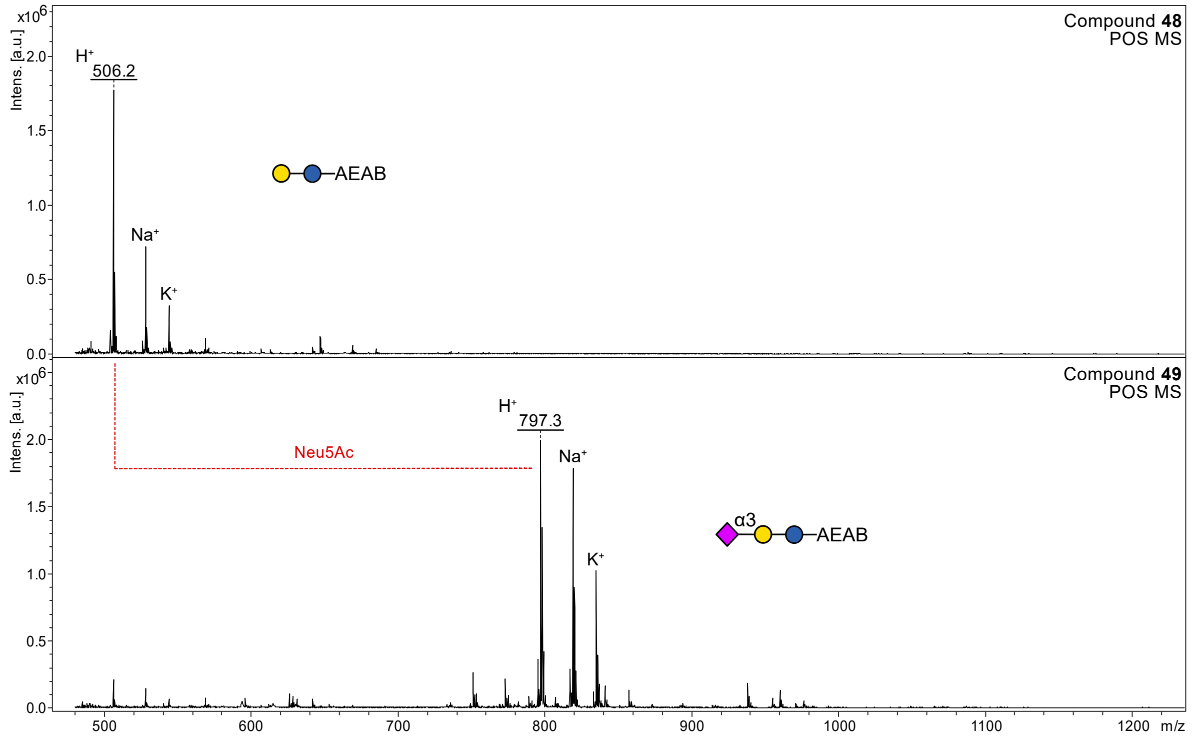

Compounds 50 and 51: Compound 49 (ganglioside GM3-type glycan) further modified with one or two additional α2,8 Neu5Ac residues (+CstII), resembling ganglioside GD3 (compound 50).

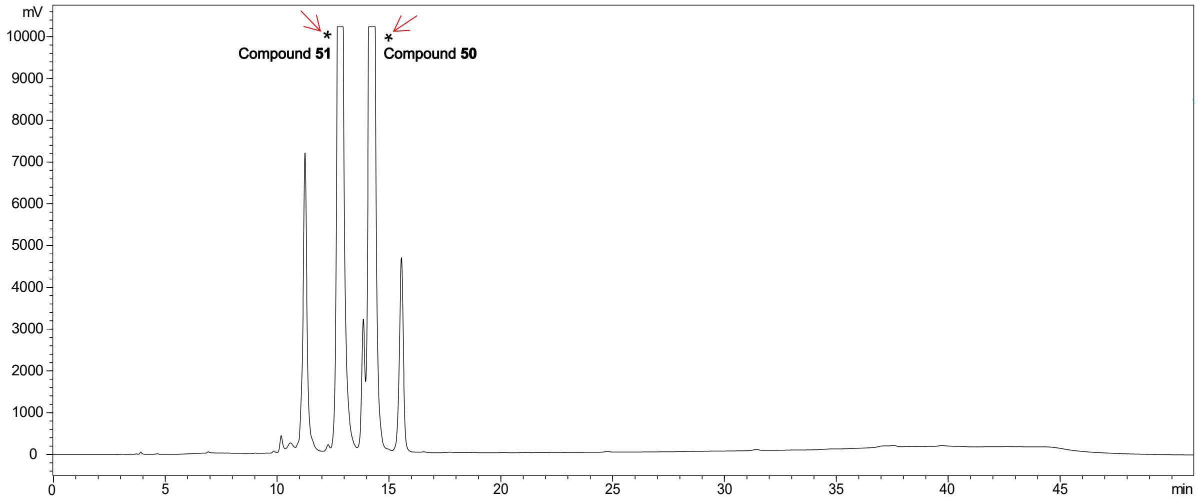

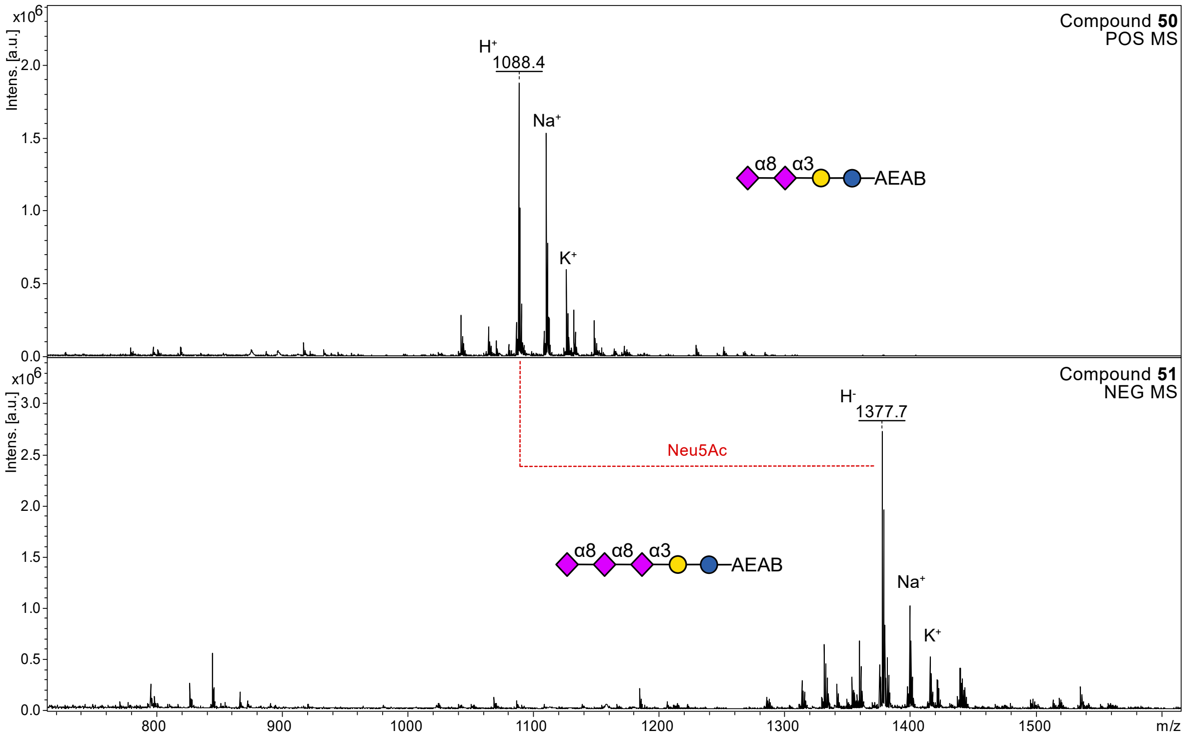

Heparan Sulphate: The GAG tetrasaccharide (Sussex Research Laboratories, Inc.) was labelled and RP-HPLC purified. MALDI-TOF NEG MS reveals two major signals, corresponding to the compound with one and two sulphates attached which were inseparable by HPLC. The presence of the monosulphated version of HS could therefore originate from an in-source fragmentation, sulphate loss during the labelling procedure or heterogeneity of the purchased native substrate which was indicated by MALDI-TOF analysis of the unlabelled sample.

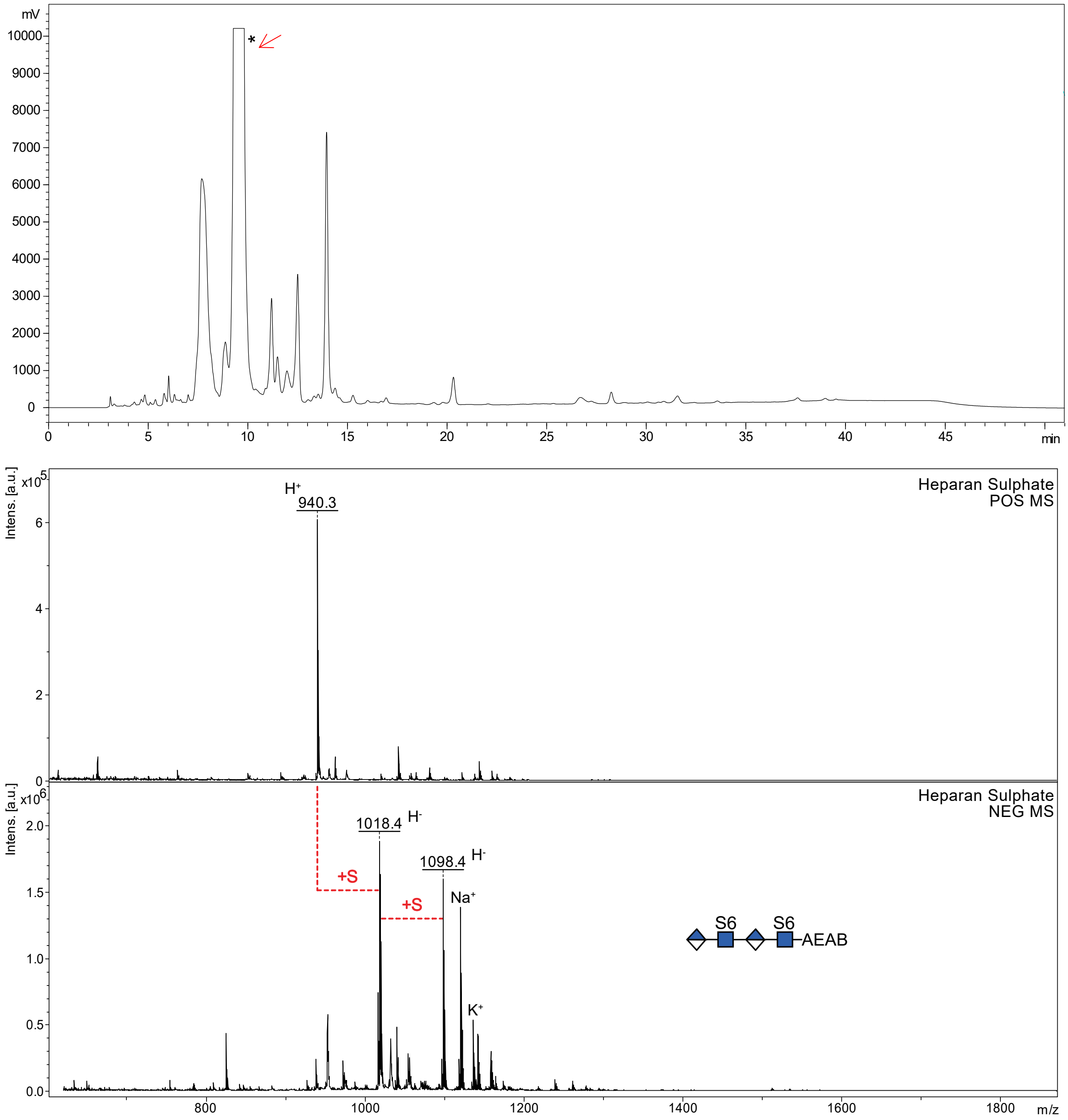

Chondroitin Sulphate (unsaturated): The GAG tetrasaccharide (Biosynth) was labelled and PGC purified. The mass difference to heparan sulphate originates from a double bond in the terminal GlcA (-18). MALDI-TOF NEG MS reveals two major signals, corresponding to the compound with one and two sulphates. The negative ion mode signals *m/z* 1000, 1102, 1124 and 1146, abbreviated as H^-, $+Na^+$, $+2Na^+$ and $+3Na^+$ can be assigned to $[M-H]^-$, $[M-2H+Na]^-$, $[M-3H+2Na]^-$ and $[M-4H+3Na]^-$, respectively.

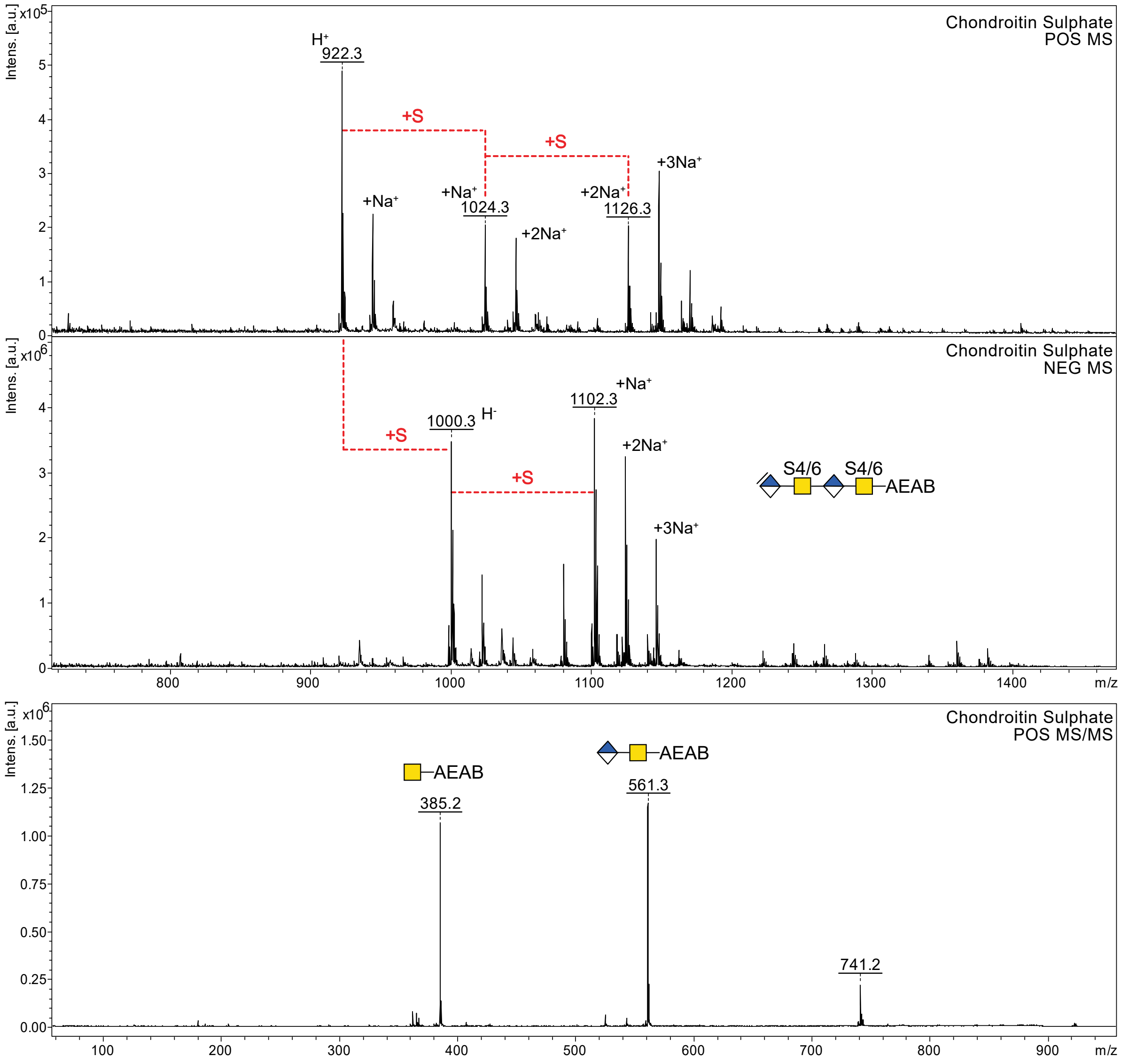

Further information regarding the glycan array analyses

**1. Glycan Binding Samples**	
Description of Sample	*Fungal/plant lectins:* Concanavalin A (ConA); *Erythrina cristagalli* lectin (ECL); *Griffonia simplicifolia* isolectin B4 and II (GSL-I and -II); *Lens culinaris* lectin (LCA); *Maackia amurensis* lectin I and II (MAA-I and -II); peanut lectin (PNA); *Ricinus communis* lectin I (RCA); *Sambucus nigra*/Elderberry Bark lectin (SNA); *Vicia villosa* lectin (VVA); wheat germ agglutinin (WGA); *Wisteria floribunda* agglutinin (WFA); soybean lectin (SBA) (Vector Laboratories); diluted 1:100.<br>*Viral proteins/human lectins and glycan specific proteins:* His-tagged Haemagglutinin (HA) of A/Puerto Rico/8/1934 (used at 200 µg/ml, provided by Prof. Reingard Grabherr (Klausberger *et al.*, 2020, *Vaccine*, 38, 859-867)); Fc-tagged human Siglec-2, Siglec-3, Siglec-7 and Siglec-8 (used at 50 µg/ml, ACROBiosystems) and Fc-tagged mouse Siglec-F and human Siglec-5 and -7 (used at 2.5 µg/ml, R&D Systems); biotinylated SiaFind α-2,3-specific, α-2,6-specific, Pan-specific and Pan-specific 2.0 (used at 5 µg/ml, except SiaFind α-2,6-specific at 0.5 µg/ml, Lectenz® Bio); His-tagged RPL-Gal1, RPL-Gal4 and RPL-Fuc1 (used at 20 µg/ml, GlycoSeLect Ltd); His-tagged human C-type lectin domain family 10 member A (CLEC-10A) (used at 10 µg/ml, R&D Systems); His-tagged C-type lectin domain family 14 member A (CLEC-14A) (used at 200 µg/ml, provided by Prof. Christoph Rademacher); human galectin-1 (used at 2 µg/ml, Biolegend, Inc); human BDNF (used at 10 µg/ml, R&D Systems).<br>*Anti-glycan antibodies:* Anti-L2/HNK-1 antibody (clone 412) (provided by Dr. Hans Bakker); diluted 1:1000, anti-Le^A antibody (Calbiochem); diluted 1:25, anti-Le^x antibody (clone L5) (provided by Prof. Melitta Schachner and Dr. Gabriele Loers); diluted 1:400; anti-Neu5Gc antibody, diluted 1:200; anti-human/mouse cutaneous lymphocyte antigen CLA (clone HECA-452), diluted 1:100 (both Biolegend, Inc).<br>All dilutions were in TSM (20 mM Tris-HCl, pH 7.4, 150 mM NaCl, 2 mM $CaCl_2$, 2 mM $MgCl_2$) supplemented with 0.05 % Tween-20, 1% bovine serum albumin, 5 mM $CaCl_2$ (hereafter TSMBB).

Assay protocol	The slides were incubated (all dilutions in TSMBB) with either:<br>(i) Biotinylated forms of recombinant or commercial lectins (diluted as indicated) followed by streptavidin AF-647 conjugated (Invitrogen, 2 µg/ml).<br>(ii) His-tagged proteins and lectins (diluted as indicated; RPL-Gal1, -Fuc1 and Fuc-4 preincubated with 1 mM $CaCl_2$, $MgCl_2$ and $MnCl_2$), followed by anti-His antibody IgG (clone HIS-1) (Sigma-Aldrich, 1:1000) and finally anti-mouse IgG AF-647 (Invitrogen, 2 µg/ml).<br>(iii) Fc-tagged Siglecs (ACROBiosystems, 50 µg/ml; R&D Systems, 2.5 µg/ml) followed by anti-human IgG (H+L) AF-647 (JacksonImmuno, 150 µg/ml)<br>(iv) Human galectin-1 (Biolegend, 2 µg/ml) followed by anti-human-galectin-1 antibody (Biolegend, 5 µg/ml) and finally anti-rat IgG AF-647 (Invitrogen, 2 µg/ml).<br>(v) Human BDNF (R&D Systems, 10 µg/ml) followed by anti-human-BDNF (R&D Systems 1:1000) and finally anti-mouse IgG AF-647 (Invitrogen 2 µg/ml)<br>(vi) Anti-L2/HNK-1 antibody (clone 412, diluted 1:1000) and anti-Le^A antibody (Calbiochem, 1:25) followed by anti-mouse IgG AF-647 (Invitrogen, 2 µg/ml); anti-Le^X antibody (clone L5, diluted 1:400) followed by anti-rat IgM AF-647 (Invitrogen, 2 µg/ml); anti-Neu5Gc antibody (Biolegend, 1:200) followed by anti-chicken IgY antibody (Sigma-Aldrich, 1:1000) and finally anti-rabbit IgG AF-647 (Invitrogen, 2 µg/ml).<br>(vii) Fluorescein labelled soybean lectin (SBA) (Vector Laboratories, 1:200) and anti-human/mouse cutaneous lymphocyte antigen CLA AF-647 (clone HECA-452) (Biolegend, 1:100)
Incubation and washing	Each incubation step was one hour; washing was by dipping ten times in TSMWB, then water, followed by drying.
Sample modifications	Fluorescent antibodies or lectins (conjugated with either AlexaFluor-647 or fluorescein) were used which detect the relevant primary antibodies or which were specific for biotin in order to detect binding of biotinylated lectins. SBA and anti-CLA were directly labelled.
**2. Glycan Library**	
Glycan description for defined glycans	Refer to Figure 2 and Figure S4A for SNFG-style structures of the library of remodelled glycans.
Glycan description for undefined glycans	Not applicable.
Glycan modifications	Substrates were derivatised with AEAB, HPLC purified to remove residual linker prior to remodelling and final RP-HPLC.
**3. Printing Surface**	
Description of Surface	NHS-ester activated glass slide
Manufacturer	SCHOTT (NEXTERION® SlideH, 1070936)

Covalent Immobilization	Glycans are terminated with a primary amine linker
**4. Arrayer (Printer)**	
Description of Arrayer	Scienion Flexarrayer S1
Dispensing mechanism	Non-contact printing with the supplier's nozzles (Type 4) and a pulse of 47 µs, ca. 109 V.
Glycan deposition	Five replicates of 0.8 nl each, five replicates of 1.6 nl each; printed as estimated by fluorescence area of HPLC peaks and normalized accordingly.
Printing conditions	Derivatised glycans and oligosaccharides were mixed 1:1 with spotting buffer (150 mM sodium phosphate pH 8.5); printing was at room temperature; arrays were left to hybridise overnight prior to blocking (50 mM ethanolamine in 50 mM sodium borate, pH 9.0) for 1 h at RT, washing (in TSM + Tween, followed by H_2O) and drying.
**5. Glycan Microarray**	
Array layout	14 subarrays per slide with 5 replicates per sample concentration.
Glycan identification and quality control	Glycans on the array were (enzymatically remodelled,) HPLC purified and verified by MALDI-TOF MS/MS and glycosidase digest where applicable.<br>Positive controls for lectin binding were included. Spotting buffer alone was used as one of the negative controls.
**6. Detector and Data Processing**	
Scanning hardware	GenePix 4400A Scanner
Scanner settings	Multiple photomultiplier tube (PMT) gain values from 800-1000 and laser power 100%, wavelength 635 nm or 532 nm where applicable
Image analysis software	GenePix Pro 7.3
Data processing	Raw .tif image files were imported to GenePix 7.3.0.0; fluorescent intensities of spots in each sub-array were analysed. Resulting data (F532 or F635 total intensity values) were exported into Excel. Mean values were calculated (spotting factor considered) and negative controls (background or spotting buffer) subtracted; standard deviation and error were calculated in Excel as well. Resulting intensities were normalized to 70000 in the GLAD tool prior to heat map generation.
**7. Glycan Microarray Data Presentation and Interpretation**	
Data presentation	Heat maps summarizing the data are shown in the main text as well as in the supplement. Bar charts with error bars where applicable shown in the Supporting Information. No software used for interpretation.
